# A GBS-based genome-wide association study reveals the genetic basis of salinity tolerance at the seedling stage in bread wheat (*Triticum aestivum* L.)

**DOI:** 10.3389/fgene.2022.997901

**Published:** 2022-09-27

**Authors:** Saba Akram, Maria Ghaffar, Ayesha Wadood, Sajid Shokat, Amjad Hameed, Muhammad Qandeel Waheed, Mian Abdur Rehman Arif

**Affiliations:** Nuclear Institute for Agriculture and Biology College, Pakistan Institute of Engineering and Applied Sciences (NIAB-C, PIEAS), Faisalabad, Pakistan

**Keywords:** GBS, abiotic stress, salt tolerance, association mapping, *Triticum aestivum* L., candidate genes, MGIDI, SNP

## Abstract

High salinity levels affect 20% of the cultivated area and 9%–34% of the irrigated agricultural land worldwide, ultimately leading to yield losses of crops. The current study evaluated seven salt tolerance-related traits at the seedling stage in a set of 138 pre-breeding lines (PBLs) and identified 63 highly significant marker-trait associations (MTAs) linked to salt tolerance. Different candidate genes were identified in *in silico* analysis, many of which were involved in various stress conditions in plants, including glycine-rich cell wall structural protein 1-like, metacaspase-1, glyceraldehyde-3-phosphate dehydrogenase GAPA1, and plastidial GAPA1. Some of these genes coded for structural protein and participated in cell wall structure, some were linked to programmed cell death, and others were reported to show abiotic stress response roles in wheat and other plants. In addition, using the Multi-Trait Genotype-Ideotype Distance Index (MGIDI) protocol, the best-performing lines under salt stress were identified. The SNPs identified in this study and the genotypes with favorable alleles provide an excellent source to impart salt tolerance in wheat.

## Introduction

Salt stress is caused by an abundance of sodium chloride (NaCl) from irrigation with brackish water and crop evaporation (Flowers and Flowers, 2005). A saline soil can be characterized as one with electric conductivity (EC) >4 dS m^−1^ at 25°C and 15% exchangeable sodium. As much as 20% of the cultivated area and 9%–34% of the irrigated agricultural land worldwide is affected by high salinity (Ghassemi et al., 1995), which ultimately leads to yield losses in crops (Jamil et al., 2011). Salinity affects >20% of modern agriculture (Li et al., 2020), making it a significant hurdle for sustainable agriculture production (Shokat and Großkinsky, 2020). Salinity affects plant growth by affecting two basic components of mineral transportation; initially, plants experience osmotic stress, which results in ion deposition and ensuing toxicity (Flowers and Flowers, 2005; Verslues et al., 2006; Munns and Tester, 2008).

While the wheat plant (*Triticum aestivum* L.) has moderate tolerance to salinity (Saddiq et al., 2019), significant yield losses still occur due to soil salinization (Oyiga et al., 2016). At 6–8 dS m^−1^ (Royo and Abió, 2003), wheat plants undergo metabolic changes that alter their life functions (Chen et al., 2016; Acosta-Motos et al., 2017). Furthermore, due to excess Na^+^ ion accumulation and low water potential of soil, hyperosmotic and hyper-ionic stress occur, in addition to primary stresses (Huang et al., 2010). These results manifest as decreased germination percentage, reduced growth, reduced yield, and changes in reproductive behavior (Hasanuzzaman et al., 2017). Among important field crops, salinity causes more damage to wheat throughout its life cycle starting from germination to maturity. Moreover, the flowering to grain filling period is the most affected stage under salt stress, which eventually leads to low grain production. In Pakistan, average wheat yield losses of up to 65% are reported in moderately saline soils (Shafi et al., 2010). Thus, it is necessary to convene all available tools of conventional and modern plant breeding tools as well as agronomic practices to hasten the development of salt-tolerant cultivars that can meet this increasing demand (Ashraf and Harris, 2004; El Sabagh et al., 2021).

Different genes are involved in the regulation of salt stress and play roles in improving plant performance under salt stress by regulating diverse mechanisms including the antioxidant defense system, Na^+^ exclusion, maintenance of Na^+^/K^+^ homeostasis, transpiration efficiency, and cytosolic K^+^ retention (Shabala and Munns, 2012; Rahman et al., 2016). Several strategies are used to increase yield under salt stress using conventional breeding tools (Hasanuzzaman et al., 2017). Several characteristics are used as indicators for wheat salinity tolerance (Colmer et al., 2006), including Na^+^ exclusion (the ability to minimize Na^+^ concentrations entering the xylem) (Munns, 2005). Salt tolerance is a quantitative trait for which numerous quantitative trait loci (QTL) have been reported in wheat at the germination, seedling, and maturity stages, as well as plant survival (Zhou et al., 2012). Previous studies suggested that the shoot Na^+^ exclusion phenotype is associated with two genes: *Nax1* (present at 2A) and *Nax2* (present at 5A) in durum wheat, a close relative of bread wheat (James et al., 2011). Many QTLs linked to salt stress tolerance in wheat have been studied previously; e.g., 65 QTLs linked to 13 different seedling traits of wheat have been identified (Masoudi et al., 2015). Similarly, QTLs associated with *NAX* were mapped to chromosome 2A and were responsible for a 10% increase in wheat biomass under salinity stress. Two QTLs—*qRNAX.7 A.3* and *qSNAX.7A.3*—mapped on chromosome 7A both showed 11% and 16% increases in salinity tolerance in wheat (Hussain et al., 2017). GWAS studies have also been conducted to identify QTLs and candidate genes linked to salinity stress in barley. Xue et al. (2009) identified 30 QTLs linked to ten different traits under salinity stress that accounted for 3%–30% the total phenotypic variation. Additionally, *HvNaX3* was mapped on the 7H chromosome of barley and was linked to salt stress tolerance (Sayed et al., 2021).

Due to the involvement of only two parents in the development of bi-parental populations, the QTL mapping approach fails to disclose the entire genetic architecture for salt tolerance (Shi et al., 2017). Therefore, genome-wide association studies (GWAS) were devised in which natural populations of hundreds of individuals with low genetic relationships are used to map desirable markers, known as marker-trait associations (MTAs) (Liu et al., 2017; Akram et al., 2021). The principle of GWAS is the linkage disequilibrium (LD), which is used to identify the relationship associations between a large number of DNA variants and traits in several genotypes from natural populations (Hu et al., 2011; Mwando et al., 2020). GWAS is a useful tool to genetically dissect biotic (Jighly et al., 2015; Arif et al., 2022; Dababat et al., 2021) and abiotic stress tolerance (Arif et al., 2012; Turki et al., 2015), physiological (Arif et al., 2021) adaptability traits (Akram et al., 2021), and nutrient uptake (Sharma et al., 2022) in wheat. While a plethora of indices has been devised to identify the best genotypes under a given environment/stress, many challenges still exist (Bizari et al., 2017). Owing to the limitations posed by previous indices (Céron-Rojas and Crossa, 2018), a new index was recently proposed based on genotype-ideotype distance and factorial analysis, which focused on the selection of superior genotypes based on multiple traits (Olivoto and Nardino, 2021).

The results of these investigations and identifications may allow the improvement of salt stress tolerance in wheat cultivars. The present study investigated a set of 138 wheat genotypes for salt-stress tolerance at the seedling stage. GWAS was applied to determine the extent of variation in response to salt stress, to identify molecular markers linked to salt tolerance, to search for candidate genes favorable for salt tolerance, and to identify salt-tolerant genotypes.

## Materials and methods

The study was conducted on a set of 138 diverse wheat pre-breeding lines (PBLs) developed at CIMMYT (Supplementary Table S1). These 138 lines were selected from a larger set of 312 PBLs previously reported (Akram et al., 2021). This germplasm was the product of a large project, the “SeeDs of Discovery”, which was implemented at CIMMYT, Mexico (Singh et al., 2018) where each line was the product of two elite (best/approved cultivars) and one exotic line (GenBank accession). The current investigation used seeds obtained from the 2018–2019 harvest.

### Experimental design and measured morphological traits

This investigation followed a completely randomized design (CRD). Initially, seeds from each line were treated with 10% NaOCl for 5 minutes followed by three washes with distilled water. In a growth chamber (Sanyo-Gallenkamp, United Kingdom) with controlled temperature of 28 ± 2°C and a 10-h photoperiod, 25 seeds of each genotype were grown on Whatman no. 1 filter paper moistened with a salt solution (Zafar et al., 2015). The experiment was conducted at three NaCl concentrations: 0, 150, and 250 mM NaCl corresponding to the control (S0), treatment 1 (S1), and treatment 2 (S2) groups, respectively.

On the eighth day of germination tests, the performance of the seedlings was assessed by recording the following morphological characteristics (Table 1). The mean value of each trait in each treatment was used for association analysis.

**TABLE 1:** Parameters measured to assess salinity tolerance.

Traits	Abbreviations	Description/formulas	References
Total germination percentage	TG	Total germination calculated by using the formula $Germination\;percentage=\frac{\;Number\;of\;seed\;germinated}{Total\;number\;of\;seed\;}\times 100$	Rajabi Dehnavi et al. (2020)
Numbers of roots	RN	Root numbers from each seedling/petro plate in control, S1 and S2	—
Coleoptile length	CL	Coleoptile length was calculated with the help of scale from each seedling/petri plate under control and all three replications of both treatments	—
Shoot length	SL	Shoot length was calculated with the help of scale from each seedling/petri plate under control and all three replications of both treatments	—
Roots length	RL	Roots length was calculated with the help of scale from each seedling/petri plate under control as well as S1 and S2	—
Root to shoot length ratio	R/S	Root to shoot length ratio was calculated with formula mentioned below $R/S=\frac{RL}{SL}$	—
Seedling vigor index	SVI	Seedling vigor index was calculated by following formula: seedling vigor=(Average root length+Average shoot length)×germination %	Kandil et al. (2012)
Relative total germination	RTG	Relative total germination was calculated by following formula: $RTG=\frac{TG\;of\;stressed\;plants}{TG\;of\;controlled\;plants}\times 100$	Kandil et al. (2012)
Relative numbers of roots	RRN	Relative numbers of roots was recorded by following formula: $RRN=\frac{RN\;of\;stressed\;plants}{RN\;of\;controlled\;plants}\times 100$	Fernandez (1993)
Relative coleoptile length	RCL	Relative coleoptile length was estimated by following formula: $RCL=\frac{CL\;of\;stressed\;plants}{CL\;of\;controlled\;plants}\times 100$	Fernandez (1993)
Relative shoot length	RSL	Relative shoot length was estimated by following formula: $RSL=\frac{Shoot\;length\;of\;stressed\;plants}{Shoot\;length\;of\;controlled\;plants}\times 100$	(Berger et al., 2012; Takahashi et al., 2015)
Relative root length	RRL	Relative root length was estimated by following formula: $RRL=\frac{RL\;of\;stressed\;plants}{RL\;of\;controlled\;plants}\times 100$	Zafar et al. (2015)
Relative root to shoot ratio	RR/S	Relative root to shoot ratio was calculated by following formula: $RR/S=\frac{R/S\;of\;stressed\;plants}{R/S\;of\;controlled\;plants}\times 100$	Berger et al. (2012)
Relative seedling vigor index	RSVI	Relative seedling vigor was estimated by following formula: $RSVI=\frac{SVI\;of\;stressed\;plants}{SVI\;of\;controlled\;plants}\times 100$	Fernandez (1993)

### DNA extraction and genotyping

The genotyping used the flag leaves at the booting stage of TC1F_5_ plants. DNA was extracted using the cetrylmethylammonium bromide (CTAB) method and quantified on a Nano-Drop instrument (http://www.diversityarrays.com/dart-application-dartseq), as described by Akram et al. (2021). A total of 58,378 high-quality SNP markers were generated, which were condensed to 6,887 SNPs by applying various stringent criteria including call rate (quality of genotyping) and reproducibility (marker consistency over replicated assays). the chromosomes, orders, and genetic distances of the mapped SNPs were obtained from the 100K-marker DArT-seq consensus map (http://www.diversityarrays.com/sequence-maps).

### Statistical analysis

SPSS 16.0 was used to generate the descriptive statistics. All other analyses, including three way ANOVA, phenotypic histograms (using the “ggplot2” package) (Wickham, 2016), circular Manhattan plot (“CMplot”) (Yin et al., 2021), and correlation (“qgraph”) (Epskamp et al., 2012) were performed in RStudio version1.0.153. To assess the association of the genotypes with the expressed phenotypes, principal component analysis (PCA) was performed using the “factoextra” package in R version 4.1.3 to reduce the dimensionality of the data (Kassambara and Mundt, 2017).

### Genetic analysis

We used STRUCTURE version 2.3.4 to analyze population structure (Pritchard et al., 2003) where the K values ranged from 1 to 9 according to Akram et al. (2021). The online Structure Harvester software was used (Earl, 2012) to obtain the result files from STRUCTURE. The bar charts of population structure were plotted using STRUCTURE PLOT (Ramasamy et al., 2014).

TASSEL V5.2.43 software was used to perform marker-trait associations using the mean data for each treatment (Bradbury et al., 2007). The current study employed an MLM model that used population structure (Q-matrix generated by the structure) and kinship (K-matrix generated by TASSEL v 5.0) matrix as covariates to avoid false positives. Markers with *p*-values <10^−3^ were defined as significant, whereas markers *p*-values less than the reciprocal of the number of markers (<1.45 × 10^−4^) were defined as highly significant associations (after Bonferroni correction) (Holm, 1979; Arif and Börner, 2020; Akram et al., 2021).

### Identification of candidate genes

Sixty-nine base-pair-length sequences for each highly significant marker including 48 bp flanking regions of SNP marker were subjected to BLAST (Basic Local Alignment Search Tool) using the NCBI (National Center for Biotechnology Information) database. The BLAST search was conducted using the genome assembly IWGSC RefSeq v2.1 (Zhu et al., 2021). Hits with 100% identity and *e*-values < 10^−4^ were selected.

### Selection of tolerant wheat genotypes

The “metan” (Olivoto and Lúcio, 2020) package in R was used to differentiate the lines according to the MGIDI, where each trait (*rX*_*ij*_) was standardized initially. This was followed by factor analysis to characterize the ideotype matrices. In the final step, an MGIDI index was computed by measuring the Euclidean distance between genotypes and ideotype scores using the following equation:

$$MGIDI=\sum_{j=1}^{f}{\left[\left(\gamma ij-\gamma j\right)2\right]}^{0.5}$$

where *γ*_*ij*_ represents the score represents of *i*th genotype (*i* = 1, 2,… ,*t*) in the *j*th factor (*j* = 1,2,… , *f*) and *t* and *f* are the number of genotypes and factors, respectively. The score of the ideal genotype was represented by *γ*_*j*_. The lower the MGIDI value of a genotype, the closer it is to the ideal genotype (Olivoto and Nardino, 2021). A ∼10% selection intensity (SI) was set to select the genotypes. Based on the ideotype concept, the traits were rescaled by assigning 0–100 values for all traits, in which 0 corresponded to the least valuable trait, and 100 to the most valuable/desired trait, to define the ideotype. In the present investigation, all traits were assigned with increasing values defining the quantitative morphological traits, which directly or indirectly affected the wheat response towards salt stress.

## Results

### Phenotypic variations

**FIGURE 1:**
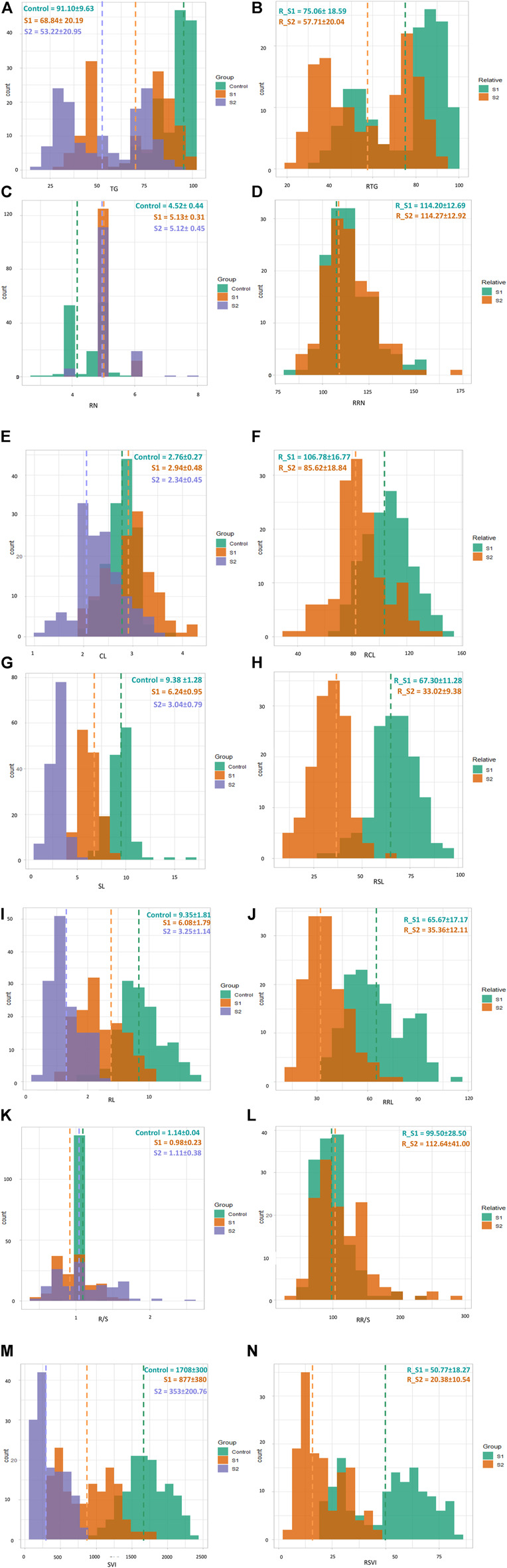
Overlaid histograms showing frequency distributions of TG **(A)**, RTG (relative) **(B)**, RN **(C)**, RRN (relative) **(D)**, CL **(E)** RCL (relative) **(F)**, SL **(G)**, RSL (relative) **(H)**, RL **(I)**, RRL (relative) **(J)**, R/S **(K)**, RR/S (relative) **(L)**, SVI **(M)**, and RSVI (relative) **(N)** across control (green), S1 (brown), and S2 (purple, for relative traits S1 (green) and S2 (brown). The vertical dotted lines indicate the mean values of each trait.

Salt stress significantly affected all traits. For example, TG decreased from 91.10 to 68.84 and 53.22 in S1 and S2, respectively, while the RTG after S1 and S2 were 75.06 and 57.71, respectively. In contrast, RN increased from 4.52 to 5.12 in S1 and 2.12 in S2, while the corresponding RRN was 114 in both treatments (S1 and S2). The CL in S1 was higher (2.94) than those in S0 and S2 (2.76 and 2.34, respectively). The RCL in S1 was also higher (106.78) than that in S2 (85.62). Among all traits, the highest decrease was observed in SL, which decreased from 9.38 (S0) to 6.08 (S1) and 3.25 (S2). The values for RSL_S1 and RSL_S2 were 67.30 and 33.02, respectively. A decreasing trend was also observed in RL, from 9.35 (S0) to 6.08 (S1) and 3.25 (S2). In contrast, the RRL in S1 was 65.67 and 35.36 in S2. The mean R/S in the control group was higher (1.14) than those for the S1 (0.98) and S2 (1.11) treatments. The relative R/S was higher in S2 (112.64) compared to that in S1 (99.50). The highest SVI was observed in the control group, with a mean value of 1708, followed by S1 (877, a decrease of 48% from the control) and S2 (353, a 91% decrease from the control). The mean RSVI in S1 (50.77) decreased to 20.38 in S2 (Figure 1; Supplementary Table S2).

Genotypes (G), treatments (T), and GxT showed significant differences in TG, RN, CL, SL, RL, R/S, SVI, RCL, RSL, RRL, RR/S, and RSVI. Among G and T, RTG showed highly significant differences, whereas no significant differences were observed for G×T. Only genotypes differed significantly in RRN, as compared to treatment plus G×T (Figure 1; Supplementary Table S2).

### Correlations

**FIGURE 2:**
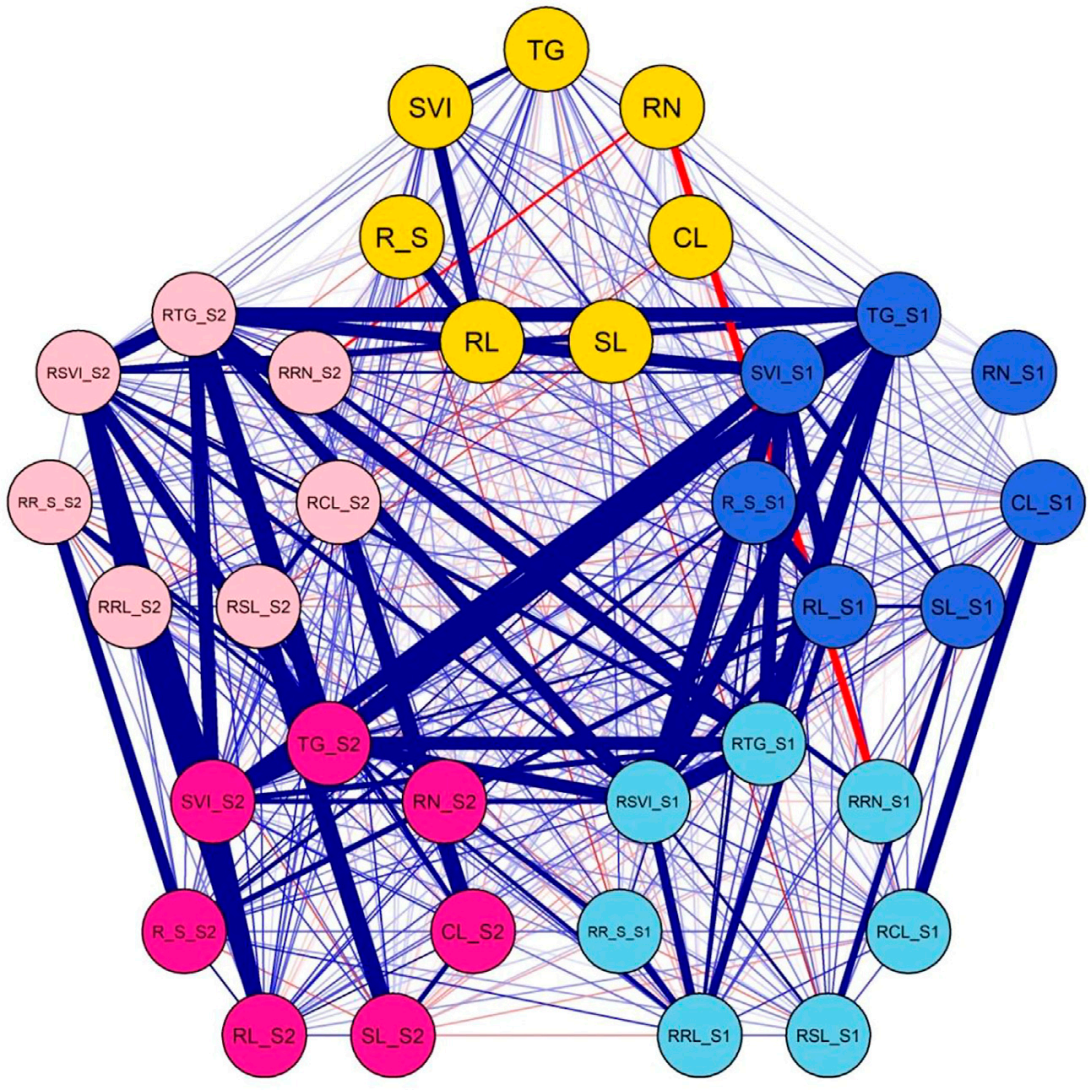
Correlations among matrixes of various traits under normal and salt-stress treatments. Yellow circle: traits under control. Blue circle: traits under S1. Sky blue circle: relative traits under S1. Bright pink circle: traits under S2. Light pink circle: relative traits under S2. Thick blue lines: highly significant correlations. Thin blue lines: significant correlations. Thick red lines: highly significant negative correlations. Thin red lines: significant negative correlations.

Most traits were positively correlated in the untreated control, although SL_S0 was negatively correlated with TG_S0 and R/S_S0 (Figure 2; Supplementary Table S4). Likewise, R/S_S0 was also negatively correlated with SVI_S0. In contrast, all traits in S1 were positively correlated except for RN_S1 which did not show any correlation with any trait. Similar trends were observed in S2, except for RN_S2, which did not show any correlation with any other trait. In addition, SL_S2 was negatively correlated with R/S_S2. The relative traits were also positively correlated in most instances with their corresponding traits under salt stress.

### Structure analysis

To correctly estimate the numbers of sub-groups in our germplasm, we plotted ΔK with a constant number of K sub-groups on the *x*-axis (Evanno et al., 2005), which showed a maximum ΔK value for K = 2 (Supplementary Figure S1). This value rose again at 5 and remained stable afterward. Therefore, we concluded that our germplasm carried five sub-populations (Supplementary Figure S2). The highest numbers of PBLs were observed in the second sub-group (Q2) followed by Q1, Q4, Q3, and Q5, with 27, 25, 23, and 18 PBLs, respectively. Supplementary Table S1 also provides information on the accessions regarding the pedigree and Q groups as identified from the STRUCTURE analysis, where the STRUCTURE results were consistent with the pedigrees of the collection. According to the pedigree, accessions with a last-crossed parent of Baj#1 were grouped in Q1 whereas most accessions with a last-crossed parent of KIRITATI were grouped in Q2. Accessions with last-crossed parents of Baj1, KACHU, and KIRITATI were grouped in Q3. The fourth subgroup included accessions in which the last-cross parent included VILLA JUARE2 F2009, while all other accessions with SUP152 as a parent in the last cross were grouped in Q5 (Supplementary Table S1).

### Association mapping

**FIGURE 3:**
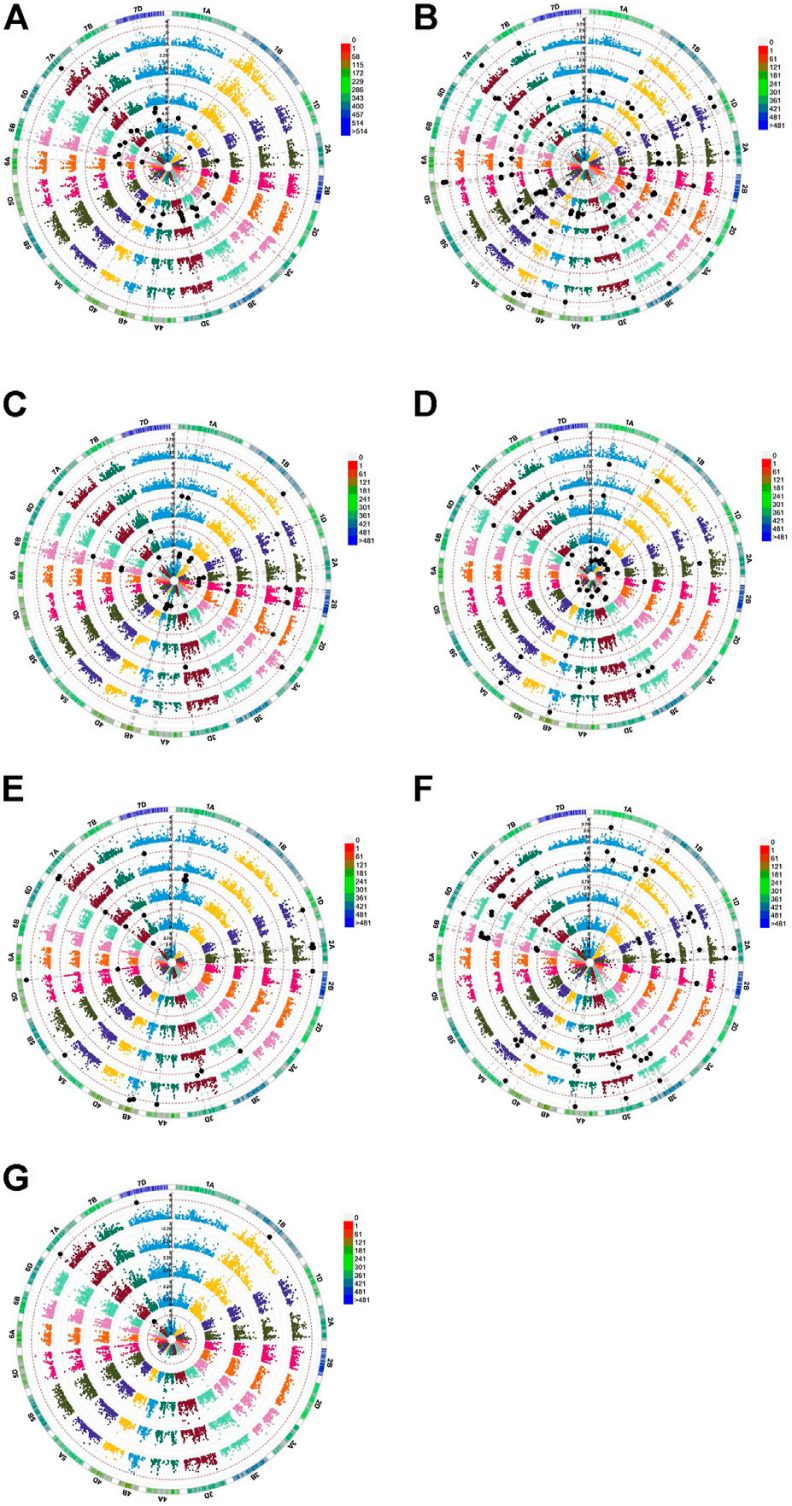
Genome-wide scan (GWAS analysis) of **(A)** TG, **(B)** RN, **(C)** CL, **(D)** SL, **(E)** RL, **(F)** R/S, and **(G)** SVI. S0 (inner circle), S1 (first outer circle), S2 (second outer circle), R_S1 (third outer circle), and R_S2 (fourth outer circle) are circular Manhattan plots in which the chromosomes are plotted at the outermost circle. The thin dotted red line indicates significance at *p* < 0.001 (−log10 = 3 or more) beyond which an association is counted as a true association (highlighted black dots). The scale between chromosomes 7D and 1A indicates the LOD threshold. The colored boxes outside on the top right side indicate the SNP density across the genome where green to red indicates less to more dense.

A total of 195 MTAs showed an LOD of ≥3 (*p* < 10^−3^) for the different traits observed in this study (Figure 3; Table 2). After Bonferroni correction, the number of associations decreased to 63 for all traits except for TG, CL, and SVI which did not show any association with any marker. Associations with *p* <1 × 10^−3^ were considered significant, while those with *p* <1.452 × 10^−4^ were considered highly significant.

**TABLE 2:** Chromosome-wide distributions of markers associated with various traits at *p* < 1 × 10^−3^ (normal text) and *p* < 1.452 × 10^−4^ (bold text).

Trait	Marker	Chr	Pos	F	*p*	*R* ^2^
CL_S2	M5289	1A	74.16	8.053285	5.25E-04	0.1227
RL_S2	M10801	1A	139.53	7.804446	6.61E-04	0.1289
SL_S2	M10801	1A	139.53	7.364397	9.75E-04	0.1176
RL_S2	M3085	1A	152.73	7.385107	9.39E-04	0.1129
**RL_S2**	**M406**	**1A**	**155.92**	**9.66784**	**1.30E-04**	**0.1529**
CL_S2	M5640	1A	159.59	7.605521	8.12E-04	0.1267
RL_S2	M11222	1A	170.55	7.735749	7.38E-04	0.1368
RR/S_S1	M8532	1A	224.72	8.297159	4.23E-04	0.1220
SL_S0	M6548	1A	235.18	9.168947	2.08E-04	0.1463
RR/S_S1	M8919	1A	480.78	8.353943	4.32E-04	0.1443
SL_S0	M7237	1A	490.67	9.146991	2.02E-04	0.1530
RR/S_S1	M7237	1A	490.67	7.462517	8.85E-04	0.1241
**RR/S_S1**	**M1426**	**1B**	**37.65**	**10.8378**	**4.98E-05**	**0.1626**
RRN_S1	M1426	1B	37.65	7.634138	7.80E-04	0.1225
**SL_S0**	**M11993**	**1B**	**61.58**	**11.5367**	**2.88E-05**	**0.1849**
RR/S_S1	M11993	1B	61.58	7.675678	7.64E-04	0.1367
SL_S0	M9230	1B	93.83	7.759434	6.77E-04	0.1218
SL_S0	M9015	1B	95	9.261137	1.83E-04	0.1447
SL_S0	M6072	1B	95	9.183687	1.96E-04	0.1498
RN_S2	M3534	1B	104	9.287996	1.76E-04	0.1384
RN_S2	M541	1B	194.87	7.783304	6.56E-04	0.1185
RN_S2	M5680	1B	205	7.901704	5.91E-04	0.1177
RSVI_S2	M7489	1B	285.98	8.025234	5.46E-04	0.1258
**SL_S0**	**M11428**	**1B**	**384.13**	**9.90536**	**1.07E-04**	**0.1584**
TG_S0	M11428	1B	384.13	9.222794	1.93E-04	0.1503
RN_S2	M11428	1B	384.13	8.169008	4.82E-04	0.1248
RCL_S2	M1718	1B	442.09	8.215706	4.73E-04	0.1363
**RN_S1**	**M9978**	**1B**	**492.15**	**11.2766**	**3.40E-05**	**0.1705**
**RR/S_S1**	**M10566**	**1D**	**93.71**	**10.6262**	**6.08E-05**	**0.1651**
**RRN_S2**	**M10810**	**1D**	**22.56**	**9.80467**	**1.29E-04**	**0.1718**
RRN_S1	M4912	1D	83.22	7.693458	7.52E-04	0.1276
**SL_S0**	**M10295**	**1D**	**90.1**	**9.63159**	**1.33E-04**	**0.1552**
RRN_S2	M8081	1D	100.36	9.542085	1.47E-04	0.1615
RCL_S1	M3676	1D	103.92	7.543196	8.35E-04	0.0709
RN_S2	M6138	1D	136.12	7.98738	5.52E-04	0.1191
RRL_S2	M10955	1D	151.27	7.340744	9.80E-04	0.1220
RN_S2	M10317	1D	164.21	9.49205	1.46E-04	0.1413
**SL_S0**	**M8113**	**1D**	**167.7**	**9.67561**	**1.36E-04**	**0.1614**
RN_S2	M7514	2A	76.95	8.021436	5.37E-04	0.1242
**SL_S0**	**M4431**	**2A**	**125.28**	**17.4073**	**3.07E-07**	**0.3133**
**RR/S_S1**	**M4431**	**2A**	**125.28**	**11.7778**	**2.48E-05**	**0.1837**
RSL_S1	M4431	2A	125.28	8.738744	3.12E-04	0.1495
**RRN_S2**	**M10796**	**2A**	**159.56**	**9.90685**	**1.09E-04**	**0.1538**
CL_S0	M1289	2A	168.8	7.575586	7.97E-04	0.1220
RN_S2	M5579	2A	214.32	7.451526	9.34E-04	0.1189
**R/S_S2**	**M765**	**2A**	**221.1**	**10.4296**	**7.22E-05**	**0.1892**
RN_S2	M765	2A	221.1	8.42717	3.97E-04	0.1543
RR/S_S2	M765	2A	221.1	7.845271	6.59E-04	0.1605
R/S_S2	M8894	2A	224.61	7.562287	8.18E-04	0.1106
**RN_S2**	**M9176**	**2A**	**231.79**	**16.8382**	**4.39E-07**	**0.3004**
**RN_S2**	**M662**	**2A**	**231.79**	**9.80564**	**1.22E-04**	**0.1573**
R/S_S2	M3109	2A	231.79	7.847723	6.22E-04	0.1085
RN_S2	M11432	2A	231.79	7.603874	7.75E-04	0.1158
R/S_S2	M3296	2A	231.79	7.386294	9.50E-04	0.1051
CL_S1	M3355	2B	7.26	7.579461	7.92E-04	0.0887
RCL_S1	M3355	2B	7.26	7.31986	9.98E-04	0.0663
RCL_S1	M2801	2B	24.73	8.93737	2.52E-04	0.0885
SL_S1	M592	2B	51.74	7.474094	8.70E-04	0.1109
RRN_S1	M2717	2B	147.53	8.397012	3.85E-04	0.1290
**RR/S_S1**	**M2717**	**2B**	**147.53**	**10.6548**	**5.47E-05**	**0.1545**
RCL_S1	M1480	2B	152.1	8.473657	3.74E-04	0.0868
RCL_S1	M8928	2B	155.58	8.837134	2.96E-04	0.0956
CL_S1	M8928	2B	155.58	7.526448	9.10E-04	0.1045
CL_S0	M6019	2B	156.75	7.508633	9.00E-04	0.1379
**RR/S_S1**	**M5489**	**2B**	**157.36**	**10.6002**	**6.12E-05**	**0.1611**
RN_S1	M5756	2B	162.47	8.266946	4.54E-04	0.1369
RN_S1	M1181	2B	198.95	9.488483	1.53E-04	0.1433
RN_S2	M7686	2D	99.87	8.163531	4.88E-04	0.1468
RN_S2	M8295	2D	113.22	9.128514	2.10E-04	0.1622
RN_S2	M10939	2D	134.09	7.609537	7.82E-04	0.1161
RN_S1	M6213	2D	153.45	8.50066	3.75E-04	0.1290
RRN_S1	M6643	2D	169.13	7.692638	7.19E-04	0.1132
SL_S0	M10941	2D	214.69	7.76266	6.78E-04	0.1241
RCL_S1	M754	2D	238.26	7.388606	9.42E-04	0.0697
**RRN_S2**	**M627**	**2D**	**289.68**	**10.8054**	**5.45E-05**	**0.1759**
**RN_S2**	**M1930**	**3A**	**75.97**	**15.3941**	**1.12E-06**	**0.2296**
RCL_S2	M1648	3A	88.71	7.598868	7.78E-04	0.1194
RN_S2	M11728	3A	229.22	8.641905	3.14E-04	0.1348
RN_S1	M4502	3B	58.82	8.172732	4.95E-04	0.1240
**RRN_S2**	**M5307**	**3B**	**68.2**	**11.2345**	**3.32E-05**	**0.1704**
**SL_S0**	**M7873**	**3B**	**103.59**	**17.5398**	**2.78E-07**	**0.3375**
**RR/S_S1**	**M7873**	**3B**	**103.59**	**11.097**	**4.32E-05**	**0.1783**
RSL_S1	M7873	3B	103.59	9.205768	2.10E-04	0.1616
R/S_S2	M1347	3B	116.53	7.685308	7.23E-04	0.1073
R/S_S2	M5248	3B	129.72	7.976771	5.93E-04	0.1179
RN_S1	M9174	3B	132.6	7.573937	8.01E-04	0.1143
RN_S1	M787	3B	159.94	7.836743	6.30E-04	0.1181
**SL_S0**	**M8515**	**3B**	**162.58**	**17.8368**	**1.55E-07**	**0.2757**
**RR/S_S1**	**M8515**	**3B**	**162.58**	**12.057**	**1.64E-05**	**0.1725**
**RSL_S1**	**M8515**	**3B**	**162.58**	**9.51185**	**1.43E-04**	**0.1382**
RR/S_S2	M8515	3B	162.58	8.461946	3.59E-04	0.1171
**RR/S_S1**	**M9138**	**3B**	**222.96**	**11.437**	**2.95E-05**	**0.1710**
**RN_S2**	**M11925**	**3B**	**227.41**	**16.3361**	**5.31E-07**	**0.2437**
CL_S0	M992	3B	294.83	8.731384	2.96E-04	0.1380
**RN_S2**	**M2025**	**3B**	**297.56**	**11.0286**	**4.09E-05**	**0.1748**
RN_S1	M4046	3B	297.56	9.507191	1.55E-04	0.1441
RRN_S2	M5581	3D	14.7	9.166748	2.15E-04	0.1532
**RR/S_S1**	**M1987**	**3D**	**107.75**	**10.5149**	**6.16E-05**	**0.1534**
RRL_S1	M7724	3D	108.86	7.690475	7.33E-04	0.1013
**RR/S_S2**	**M1019**	**3D**	**116.66**	**10.2482**	**8.18E-05**	**0.1494**
RN_S2	M2318	3D	184.67	7.958473	5.68E-04	0.1375
CL_S2	M4388	3D	265.96	8.999231	2.25E-04	0.1322
**SL_S0**	**M1398**	**4A**	**49.56**	**12.2238**	**1.43E-05**	**0.1889**
RSL_S1	M1398	4A	49.56	7.399761	9.21E-04	0.1075
RN_S1	M10965	4A	106.71	8.44769	4.01E-04	0.1404
**SL_S0**	**M11711**	**4A**	**180.05**	**20.4829**	**3.24E-08**	**0.3835**
**RR/S_S1**	**M11711**	**4A**	**180.05**	**10.8836**	**5.15E-05**	**0.1755**
**RSL_S1**	**M11711**	**4A**	**180.05**	**10.1833**	**9.21E-05**	**0.1798**
**RN_S1**	**M5589**	**4A**	**215.47**	**10.3647**	**7.31E-05**	**0.1705**
**RN_S1**	**M10678**	**4A**	**215.47**	**10.1663**	**8.65E-05**	**0.1679**
**RN_S1**	**M3948**	**4A**	**215.47**	**10.0451**	**1.02E-04**	**0.1527**
RN_S1	M1034	4A	215.47	8.555139	3.47E-04	0.1294
RN_S1	M2701	4A	215.47	7.851619	6.27E-04	0.1329
CL_S0	M8745	4A	215.47	7.818745	6.58E-04	0.1267
RR/S_S1	M10341	4A	219.65	7.883817	6.65E-04	0.1269
RR/S_S1	M2690	4A	221.42	8.133403	5.28E-04	0.1297
RRN_S2	M5221	4A	221.42	7.694232	7.42E-04	0.1237
CL_S0	M10528	4B	70.22	7.35443	9.94E-04	0.1170
RN_S1	M1225	4B	76.73	7.907482	5.99E-04	0.1193
RN_S1	M7413	4B	76.73	7.602701	8.52E-04	0.1753
CL_S0	M9224	4B	78.23	8.734255	3.03E-04	0.1489
RN_S1	M10770	4B	78.61	9.461426	1.62E-04	0.1567
CL_S0	M3043	4B	85.28	9.325857	1.71E-04	0.1503
RSL_S2	M10038	4B	90.17	8.880234	2.63E-04	0.1503
RRL_S2	M10038	4B	90.17	8.608923	3.33E-04	0.1499
SL_S0	M10038	4B	90.17	7.700859	7.35E-04	0.1255
SL_S2	M8833	4B	108.27	8.390578	4.21E-04	0.1387
RRN_S2	M4320	4D	1.14	9.312591	1.78E-04	0.1505
**RRN_S2**	**M4103**	**4D**	**34.78**	**9.93443**	**1.05E-04**	**0.1556**
**RRN_S2**	**M3343**	**4D**	**50.31**	**10.0131**	**9.62E-05**	**0.1525**
**RR/S_S1**	**M1974**	**4D**	**157.4**	**11.1122**	**4.19E-05**	**0.1728**
RN_S1	M11094	5A	85.73	9.569622	1.48E-04	0.1584
RSL_S2	M11935	5A	97	7.910871	6.14E-04	0.1332
**RR/S_S1**	**M8885**	**5A**	**113.15**	**11.209**	**3.87E-05**	**0.1720**
**SL_S0**	**M8885**	**5A**	**113.15**	**10.5227**	**6.84E-05**	**0.1954**
RSL_S1	M8885	5A	113.15	7.908211	6.32E-04	0.1305
RR/S_S1	M5275	5A	113.33	7.593138	8.15E-04	0.1151
**RRN_S1**	**M11486**	**5A**	**161.09**	**12.2524**	**1.43E-05**	**0.1818**
RN_S0	M10895	5A	161.09	7.412972	9.41E-04	0.1337
RR/S_S1	M56	5A	167.78	9.31266	1.78E-04	0.1370
RN_S2	M7106	5A	199.92	7.444092	9.25E-04	0.1140
**RN_S2**	**M4314**	**5A**	**209.83**	**13.8064**	**4.18E-06**	**0.2143**
**RN_S2**	**M3034**	**5A**	**229.39**	**16.1937**	**6.55E-07**	**0.2476**
CL_S0	M1688	5A	285.2	7.362705	9.64E-04	0.1131
**RN_S2**	**M4710**	**5B**	**56.97**	**16.4673**	**5.46E-07**	**0.2679**
**RN_S2**	**M2139**	**5B**	**56.97**	**9.73039**	**1.24E-04**	**0.1701**
RN_S1	M10042	5B	83.23	7.506657	8.63E-04	0.1135
**RN_S1**	**M1450**	**5B**	**125.96**	**10.6565**	**5.93E-05**	**0.1617**
RRN_S2	M5772	5B	145.97	7.384505	9.33E-04	0.1106
RN_S2	M8958	5B	169.96	8.360985	4.36E-04	0.1412
SL_S0	M5828	5B	256.53	8.074973	5.05E-04	0.1248
**RN_S2**	**M11102**	**5D**	**195.73**	**17.1946**	**2.72E-07**	**0.2852**
**RN_S2**	**M7775**	**5D**	**210.31**	**17.2919**	**2.72E-07**	**0.2875**
RN_S2	M8011	5D	223.19	7.825985	6.82E-04	0.1365
RN_S1	M7245	5D	228.94	8.147855	4.91E-04	0.1232
RL_S1	M4090	5D	228.94	7.589824	8.04E-04	0.1074
**RRN_S2**	**M337**	**5D**	**232.48**	**10.0153**	**9.55E-05**	**0.1504**
RRL_S2	M337	5D	232.48	9.507016	1.48E-04	0.1531
**RN_S2**	**M5347**	**6A**	**65.28**	**16.3246**	**5.36E-07**	**0.2435**
**RN_S2**	**M530**	**6A**	**73.17**	**16.3465**	**5.13E-07**	**0.2436**
RN_S2	M8998	6A	167.65	7.676664	7.57E-04	0.1279
CL_S1	M6385	6B	23.32	7.343408	9.77E-04	0.0848
RN_S2	M10569	6B	45.17	9.014767	2.31E-04	0.1536
CL_S0	M5384	6B	62.83	7.539925	8.44E-04	0.1175
**RR/S_S1**	**M4362**	**6B**	**69.05**	**10.4907**	**6.81E-05**	**0.1701**
CL_S2	M2278	6B	132.99	7.923197	5.93E-04	0.1185
RR/S_S2	M5749	6D	0	7.639943	7.99E-04	0.1119
RN_S2	M3987	6D	25.79	8.724641	3.24E-04	0.1565
**RR/S_S1**	**M11763**	**6D**	**90.8**	**10.7309**	**5.45E-05**	**0.1574**
**SL_S0**	**M1188**	**6D**	**121.22**	**17.9048**	**1.47E-07**	**0.2767**
**RR/S_S1**	**M1188**	**6D**	**121.22**	**11.4818**	**2.66E-05**	**0.1643**
RSL_S1	M1188	6D	121.22	9.290898	1.74E-04	0.1350
SL_S2	M10197	6D	181.69	8.790818	3.02E-04	0.1408
RL_S2	M10260	7A	38.12	7.674994	8.05E-04	0.1508
**RR/S_S1**	**M10555**	**7A**	**45.99**	**11.291**	**3.36E-05**	**0.1933**
**RR/S_S1**	**M10047**	**7A**	**45.99**	**10.9379**	**4.80E-05**	**0.1637**
**RR/S_S1**	**M9141**	**7A**	**45.99**	**10.6199**	**5.90E-05**	**0.1581**
RRN_S1	M9141	7A	45.99	7.803681	6.66E-04	0.1198
RL_S1	M8832	7A	55.34	9.453854	1.74E-04	0.1710
RTG_S2	M3854	7A	145.43	9.03094	2.34E-04	0.1356
TG_S2	M3854	7A	145.43	8.309385	4.36E-04	0.1223
**RRN_S2**	**M9550**	**7A**	**170.91**	**10.5026**	**6.22E-05**	**0.1581**
**RL_S0**	**M38**	**7A**	**214.7**	**10.7602**	**6.60E-05**	**0.2053**
SVI_S0	M38	7A	214.7	7.811965	7.53E-04	0.1426
**RN_S1**	**M9660**	**7A**	**276.04**	**11.7383**	**2.18E-05**	**0.1768**
RR/S_S1	M9581	7B	114.48	7.671001	7.70E-04	0.1140
RL_S1	M9431	7B	117.72	7.838873	6.23E-04	0.1061
SL_S2	M7039	7B	234.97	9.060685	2.34E-04	0.1565
RSL_S2	M3286	7D	113.89	8.872057	2.51E-04	0.1323
RN_S2	M7616	7D	205.95	8.168864	4.70E-04	0.1244
RSVI_S2	M3286	7D	113.89	7.843709	6.20E-04	0.1176
RRL_S1	M6322	7D	117.37	7.653348	7.58E-04	0.0988
SL_S0	M10389	7D	369.62	7.566639	8.88E-04	0.1370
RR/S_S1	M10389	7D	369.62	7.493323	9.46E-04	0.1260
SL_S2	M3286	7D	113.89	7.350426	9.62E-04	0.1068

**FIGURE 4:**
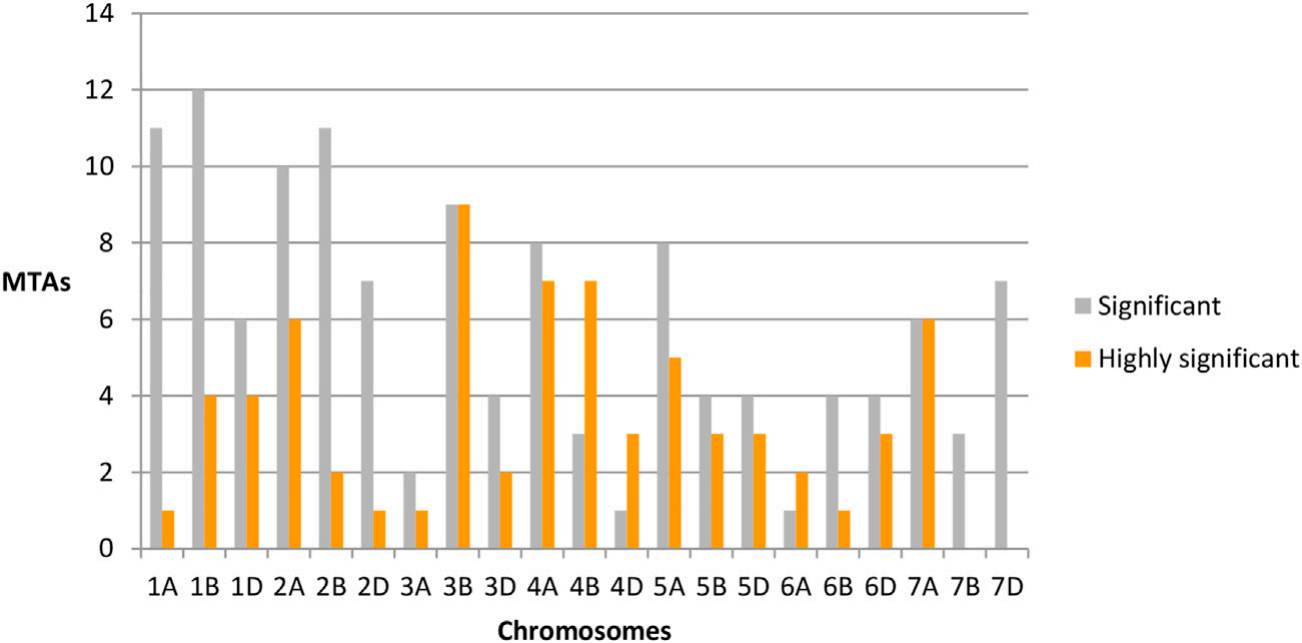
Chromosome-wide distribution of significant (gray) and highly significant (orange) marker-trait associations.

The distributions of these MTAs in the wheat genome varied considerably. For example, chromosome 3B exhibited the highest (18 including nine highly significant) number, whereas chromosomes 3A (three including one highly significant), 6A (three including two highly significant) and 7B (three significant) exhibited the lowest numbers of MTAs (Table 2). Sixteen MTAs each were detected on chromosomes 2A (including six highly significant associations) and 1B (including four highly significant associations). Chromosome 4A carried 15 MTAs, including seven that were highly significant. There were 13 MTAs on each of chromosomes 5A (including five highly significantly) MTAs. Chromosome 2B also carried 13 MTAs, including two that were highly significant. Likewise, chromosomes 7A and 1A had 12 MTAs each, with six and one highly significant MTA, respectively. Similarly, 10 MTAs were detected on chromosomes 1D (including four highly significant associations) and 4B (including seven highly significant associations), respectively. Chromosome 2D showed eight significant MTAs, including one that was highly significant. Chromosomes 5B, 5D, 6D, and 7D each showed seven MTAs; each chromosome contained three highly significant MTAs except for 7D, which showed no highly significant MTAs. In addition, six MTAs were detected on chromosomes 3D (including two highly significant MTAs), while 6B showed five (including one highly significant) MTAs. Finally, four (including three highly significant MTAs) MTAs were detected on chromosome 4D (Figure 4).

Regarding traits, RN_S2 showed the highest number of MTAs (35 including thirteen that were highly significant), whereas RL_S0, RN_S0, RTG_S2, SL_S1, SVI_S0, TG_S2, and TG_S0 showed the lowest numbers of MTAs (one) for each trait. Twenty-eight MTAs (including eighteen that were highly significant) and 22 MTAs (including six that were highly significant) were detected for RR/S_S1 and RN_S1 respectively. We also detected 20 MTAs associated with SL_S0, thirteen with RRN_S2, and seven with RSL_S1 including eleven, eight, and two that were highly significantly MTAs respectively. Nine MTAs associated with SL_S0 (with no highly significant association), while six MTAs were detected for R/S_S2, RCL_S1 (including one significant MTA), and RRN_S1. Five MTAs were linked with RL_S2 (one significantly MTA) and SL_S0 (with no highly significant association). RR/S_S2 and CL_S2 were linked to four markers. RR/S_S2 was associated with one highly significant MTA. We also detected three MTAs for CL_S1, RL_S1, RRL_S2, and RSL_S2 and two MTAs for RCL_S2, RRL_S1, and RSVI_S2, with no highly significant association (Table 2).

### Candidate genes

BLAST was performed for highly significant SNP markers. Hits with 100% identity and *e*-values < 10^−4^ were selected. Twelve candidate genes were identified through this analysis. These included putative disease resistance RPP13-like protein 1, disease resistance protein RGA2-like (involved in conferring disease resistance), glycine-rich cell wall structural protein 1-like (part of the cell wall that acts as a structural protein), two metacaspase-1-like proteins (play roles in programmed cell death), sphinganine C4 monooxygenase 1-like (plays a role in sphingolipid biosynthesis), two 60S ribosomal protein L22-like proteins (ribosomal proteins), glyceraldehyde-3-phosphate dehydrogenase GAPA1, subtilisin-like protease SBT1.7, mRNA-decapping enzyme-like protein, and calmodulin-binding protein 60 D-like (involved in different stress responses, including biotic and abiotic responses). These candidate genes along with their physical locations and functions are shown in Table 3.

**TABLE 3:** Identification of candidate genes involved in stress tolerance.

Sr. No.	Marker/trait/chr	Gene ID	Location	Length	Candidate genes
1	M11428/SL_S0/IB	LOC123145204	Chr1B: 648,595,158-648,602,293	4,385	*Triticum aestivum* putative disease resistance RPP13-like protein 1
2	M10810/RRN_S2/1D	LOC123179818	Chr1D: 6,607,035-6,641,306	3,071	*Triticum aestivum* disease resistance protein RGA2-like
3	M10796/RRN_S2/5D	LOC123121043	Chr5D: (507,153,271-507,153,732)	462	*Triticum aestivum* glycine-rich cell wall structural protein 1-like
4	M1930/RN_S2/3A	LOC123060422	Chr3A: 82,083,711-82,086,686	2,629	*Triticum aestivum* metacaspase-1-like (LOC123060422)
5	M5307/RRN_S2/3B	LOC123064730	Chr3B: 57,612,905-57,618,157	2,238	*Triticum aestivum* glyceraldehyde-3-phosphate dehydrogenase GAPA1, chloroplastic-like (LOC123064730)
6	M8515/RSL_S1.SL_S0.RR/S_S1/3B	LOC123072063	Chr3B: 734,929,010-734,930,868	1,478	*Triticum aestivum* metacaspase-1-like (LOC123072063)
7	M11711/RSL_S1.SL_S0.RR/S_S1/4A	LOC123082947	Chr4A: 659,640,114-659,640,987	738	*Triticum aestivum* sphinganine C4 monooxygenase 1-like (LOC123082947)
8	M3948/RN_S1/4A	LOC123088273	Chr4A: 709,761,323-709,762,376	1,054	*Triticum aestivum* 60S ribosomal protein L22-like (LOC123088273)
9	M10678/RN_S1/4A	LOC123088271	Chr4A: 709,405,204-709,406,325	1,122	*Triticum aestivum* 60S ribosomal protein L22-like (LOC123088271)
10	M4710/RN_S2/5A	LOC123124722	Chr5A: 301,269,821-301,272,263	2,443	*Triticum aestivum* subtilisin-like protease SBT1.7 (LOC123124722)
11	M7775/RN_S2/5D	LOC123123671	Chr5D: 542,615,453-542,621,186	2,031	*Triticum aestivum* mRNA-decapping enzyme-like protein (LOC123123671)
12	M11763/RR/S_S1/6D	LOC123144592	Chr6D: 260,335,200-260,341,291	2,089	*Triticum aestivum* calmodulin-binding protein 60 D-like (LOC123144592)

### Multivariate analysis

**FIGURE 5:**
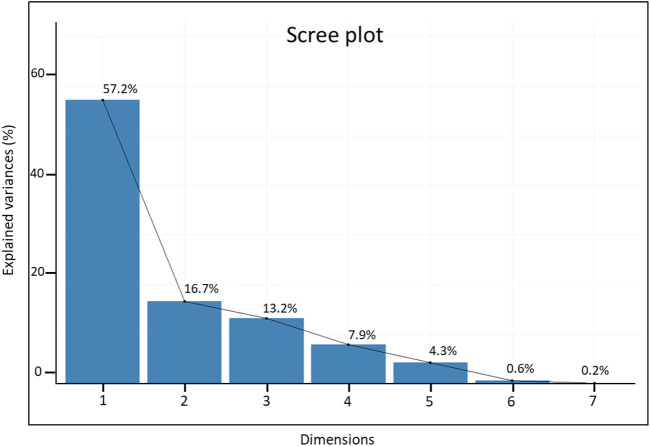
Scree plot showing the contributions of the first seven principal components.

**FIGURE 6:**
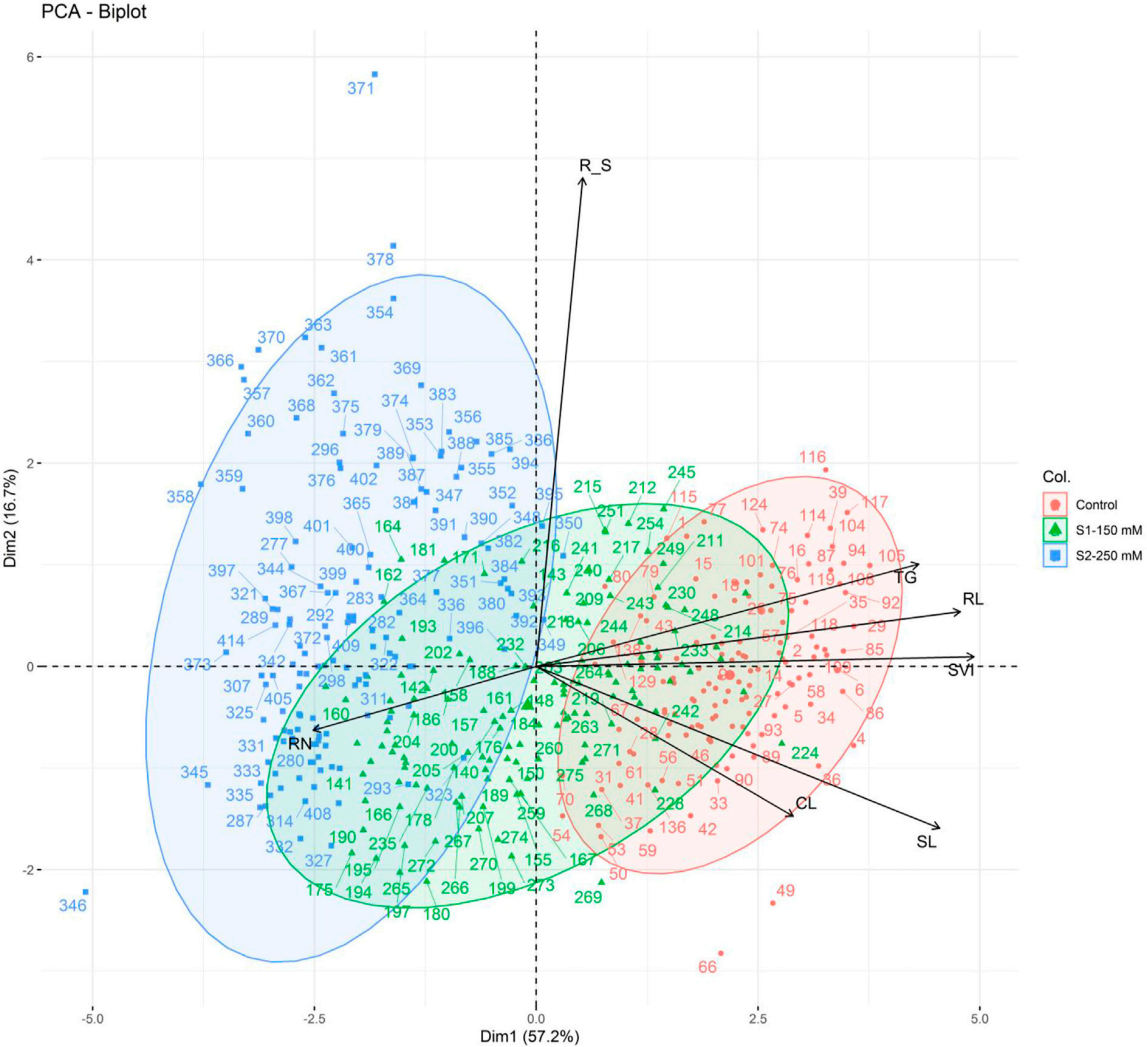
PCA of the morphological traits measured in 138 wheat genotypes in control (red) and salt stress [S1-150 Mm NaCl (blue) and S2-250 Mm NaCl)] conditions based on the first two components. TG, total germination percentage; RN, numbers of roots; CL, coleoptile length; SL, shoot length; RL, root length; R_S, root to shoot length ratio; SVI, seedling vigor index.

The first two principal components explained significant variation (73.9% of the overall variation), with eigenvalues ≥1 (Figure 5), where PC1 accounted for 57.2% of the total variation and was positively correlated with all traits except RN. The second PC explained 16.7% of the total variation and was mainly influenced by R_S, TG, RL, and SVI. All traits were sorted into three groups (I–III) based on their distributions in the PCA biplot quadrants: group I included R_S, TG, RL, and SVI; group II consisted of SL and CL; and group III contained only RN (Figure 6). The bi-plot analysis also highlighted the correlation between the examined traits: The sharp angle between trait vectors indicated a positive correlation, while obtuse and right angles indicated negative and no correlations between the parameters, respectively.

## Discussion

Wheat seedling development has three parts: germination, emergence, and early growth. All three stages are especially sensitive to salinity stress (Jamil et al., 2005). The most crucial stage of plant development is germination (Song et al., 2008). Salinity tolerance at the germination stage may provide the ultimate yield gains in terms of grains produced (Jajarmi, 2009). Salt stress also significantly affected the overall performance of the germplasm in the present study. TG decreased by 24% and 42% in S1 and S2 compared to the control. TG was also highly correlated with RN, CL, SL, RL, and SVI. Highly significant differences between genotypes and treatments were observed for all traits. The same trends were also observed for the relative values of all traits under all treatments. Salinity stress affected all genotypes during S1 and S2 treatments compared to the control. The most adverse effects of salt stress were observed at S2, in which the TG dropped to 68% (TG_S2) from 91% (TG_S0). Other important seedling traits like CL, SL, RL, and SVI also decreased in the presence of elevated salt stress (S2), consistent with the findings reported by El Hehdaey et al. (2011).

Owing to the significance of the germination stage in plant tolerance against salinity stress (Munns and James, 2003; Singh et al., 2012), germination tests are among the most suited approaches for the early-stage screening of germplasm collections for salt tolerance (Munns et al., 2006; Aflaki et al., 2017). In the present study, chromosomes 1B (at 384.13 cM) and 7A (at 145.43) carried MTAs associated with TG under control and salt stress, respectively. Batool et al. (2018) also reported one QTL associated with standard germination on chromosome 1B. A recent report also underscored the importance of loci on chromosome 2B with respect to germination under post-abiotic stress in wheat (Arif and Börner, 2020). Likewise, a QTL related to germination under abiotic stress (experimental aging) has also been reported on chromosome 7A (Arif et al., 2012).

The root is the first plant organ that experiences salt stress; thus, it plays a significant role in sensing the salinity level in the nearby environment (Galvan-Ampudia et al., 2013; Robbins et al., 2014) by signal transduction (Jiang et al., 2012; Choi et al., 2014). During salt stress, the cell cycle activity of the root meristem is reduced, resulting in reduced growth (West et al., 2004). Hence, RN and RL are important criteria in estimating the salinity tolerance in wheat. In our study, RN increased by 13% in both S1 and S2 compared to S0, with significant differences between G, T, and GXT. In contrast to RN, RL showed a significant decrease in S1 (35%) and S2 (65%) compared to the control. The same drop in RL was observed for relative traits, consistent with previous reports (Duan et al., 2013; Julkowska and Testerink, 2015). RL was highly correlated with SVI, whereas RN showed highly positive correlations with CL, SL, RL, and R/S. Previous studies have provided evidence of reduced root length due to elevated salt stress. A total of 58 MTAs linked to RN were detected on all chromosomes except for 1A, 4D, and 7B. Literature related to RN under salinity stress is scarce. However, our findings are consistent with those reported by Li et al. (2011), Salem and Mattar (2014), and Rufo et al. (2020). Likewise, chromosomes 1A, 1D, 3D, 4B, 5D, 7A, 7B, and 7D carried MTAs for RL and RRL under control and salt stress, similar to the results reported by Batool et al. (2018) and Salem and Mattar (2014). Marker M4090 present on 5D at 228.94 cM was linked to RL and RN, which showed a pleiotropic effect by controlling two characters on the same chromosome and location Markers controlling more than one trait are important for the improvement of salt stress tolerance in wheat (Batool et al., 2018).

The coleoptile protects the first leaf of the future wheat plant, which also functions as the driver to propel the leaf outside the soil crust. Coleoptile strength and success is tantamount to the successful establishment and early plant vigor. Stress, may enhance the CL and shorten the SL (Zhang and Wang, 2012). In the present study, a 6% increase in coleoptile length was observed at S1 compared to S0. A 15% decrease was observed at S2 compared to S0. The CL also showed highly significant positive correlations with SL, RL, and R/S. Saboora et al. (2006) also observed the same trends in CL increases and decreases at moderate (75 mM) and higher levels of salt stress (150, 225, 300, and 375 mM). Moud and Maghsoudi (2008) reported that salt stress inhibited CL more than root growth. MTAs linked with CL under various conditions were detected on the chromosomes of group three and chromosomes 1A, 2A, 2B, 4A, and 4B. Li et al. (2011) reported QTLs associated with CL on chromosome 4B and 6B. In addition, two major QTLs of CLs were reported on chromosome 4B and 4D (Sidhu et al., 2019), corroborating our findings. Salem and Mattar (2014) reported QTLs related to CL under salinity stress at NaCl concentrations of 0 (on chromosome 1D), 150 (on 1D and 3BS), and 250 (on 1D, 4B, and 7D) mM; however, we detected no MTA for CL on chromosome 1D.

SL showed highly significant differences among treatments, with 33% and 68% decreases in S1 and S2, respectively. SL showed highly significant positive correlations with RL and R/S and a highly significant negative correlation with SVI. Bilkis et al. (2016) reported a 6%–36% decrease in shoot length under salt treatment. These findings were also similar to those of Datta et al. (2009) and Alom et al. (2016), who reported significantly reduced SL and RL at salinity levels <125 mM NaCl. SL is an important factor in the selection of genotypes against salt stress. The 20 MTAs of SL in various conditions were distributed on 14 different chromosomes, corroborating previous findings (Ghaedrahmati et al., 2014; Batool et al., 2018; Liu et al., 2018). The MTAs on chromosome 4B for SL_S0 (M8833 at 108.27 cM) and SL_S2 (M10038 at 90.17 cM) corresponded to the dwarfing gene *Rht-B1* on chromosome 4BS (Arif et al., 2021; Mo, 2018).

The R/S ratio is also disturbed under salt stress; however, this response is more tied to water stress than to salt stress (Hsiao and Xu, 2000). Increased RL as compared to SL may lead to the preservation of a large proportion of toxic ions in the roots and ameliorate their movement to the shoot, thus helping plant survival under salt stress (Cassaniti et al., 2009; Cassaniti et al., 2012). Çamlıca and Yaldız (2017) reported a decreased root/shoot length ratio with increasing salinity doses, with a greater reduction in root length than shoot length. In other words, the root length was more negatively affected than shoot length by increasing salinity doses. Landjeva et al. (2008) identified two QTLs on chromosome 3DL (*QRSRc.ipk-3D.1* and *QRSRc.ipk-3D.2*) and one QTL on chromosome 6DL (*QRSRp.ipk-6D*) that were associated with the R/S ratio under osmotic stress. We detected two highly significant MTAs (M1987 at 107.75 cM and M1019 at 116.66 cM) in S1 and S2 on chromosome 3D. Two MTAs, M11763 at 90.8 cM and M1188 at 121.22 in S1, were also detected on chromosome 6D, suggesting that both these loci were associated with R/S control under both salt and osmotic stresses.

Damaged plants show decreased viability, as represented by SVI (Copeland and McDonald, 2012). This is the most important trait for screening against salt stress. SVI is the product of many different factors and is related to genetics and environmental influences. The results of the current study showed highly significant differences between G, T, and G×T. SVI dropped by 48% and 91% at S1 and S2, respectively, from S0, implying that S2 was critical and damaging. A similar decrease was reported in *Brassica napus* (Batool et al., 2015) and *Hibiscus* species (Rashmi and Naik, 2014). In contrast (Batool et al., 2015), various QTLs associated with SVI at 150 mM NaCl on chromosomes 2A (*QSVI.2A.SG*), 4A (*QSVI.4A.SG*), 6D (*QSVI.6D.SG*) and 7B (*QSVI.7B.SG*) have been reported. We detected one MTA (M38 at 214.7 cM) on chromosome 7A that was associated with SVI_S0 on chromosome 7A. M38 was also associated with RL. Likewise, two MTAs (M7489 at 258.98 cM on chromosome 1B and M3286 at 113.89 on chromosome 7A) were also detected with RSVI_S2 on chromosome 7A, indicating that a wide variety of loci determine the SVI in wheat and are strongly dependent on RL and SL.

The BLAST search against the highly significant SNP markers identified in the present study revealed candidate genes involved in various stress conditions in plants. One such candidate gene (glycine-rich cell wall structural protein 1-like) was found on chromosome 5D. Glycine-rich proteins (GRP) are reportedly involved in stress responses including salinity, drought, etc., in many plants (Czolpinska and Rurek, 2018). Moreover, two candidate genes on chromosome 1B and 1D are involved in disease resistance. Additionally, multiple SNP markers matched candidate genes; i.e., metacaspase-1-like protein. Metacaspase-1 has a predominant role in the regulation of programmed cell death. The endoplasmic reticulum (ER) regulates protein synthesis. High salinity levels cause ER stress through the accumulation of misfolded proteins, which can lead to unfolded protein response (UPR) as a stress response mechanism. The UPR mechanism reverses misfolded proteins. UPR failure activates programmed cell death (Yusof et al., 2021). Metacaspase genes are key regulators of programmed cell death and might be the cardinal components of the saline stress pathway. Another candidate stress response gene (glyceraldehyde-3-phosphate dehydrogenase GAPA1, chloroplastic-like) located on chromosome 3B was also detected. Plastidial *GAPA1* has an abiotic stress response role in wheat and other plants (Chang et al., 2015; Li et al., 2019) and was associated with the M5307 marker related to RRN in S2 treatment at chromosome 3B. Munoz-Bertomeu et al. (2009) described the role of plastidial *GAPA1* in root development as this gene is involved in the biosynthesis of serine, which is essential for root development. The present study also showed the association of plastidial *GAPA1* in root development. Another gene, sphinganine C4-monooxygenase 1-like, involved in sphingolipid biosynthesis was also identified. Sphingolipids are ubiquitous and present in all types of plants. They comprise parts of plant cell membranes and endo-membranes. They also play roles in plant stress responses (Huby et al., 2020). Furthermore, another important candidate gene, calmodulin-binding protein 60 D-like, aligned to the M11763/RR/S_S1/6D marker. which corresponds to the shoot-to-root length ratio and is also involved in environmental stress responses in plants (Zeng et al., 2015). Calmodulin binding proteins play a significant role in plant growth, which corresponds to the results of the present study. Another stress response gene, subtilisin-like protease SBT1.7, was identified by the analysis in the present study. This gene plays a role in biotic stress response (Meyer et al., 2016). In addition, mRNA-decapping enzyme-like protein and 60S ribosomal protein L22-like were also identified as candidate genes. Kawa and Testerink (2017) and Liu et al. (2019) reported the role of both genes in salt stress response. Moreover, mRNA-decapping contributes to the regulation of ABA signaling (Munoz-Bertomeu et al., 2009). ABA mediates many developmental programs in plants, including seed dormancy or root growth (Finkelstein, 2013). The analysis of the M7775/RN_S2/5D marker in this study also suggested the involvement of the mRNA-decapping enzyme-like protein in root development.

**FIGURE 7:**
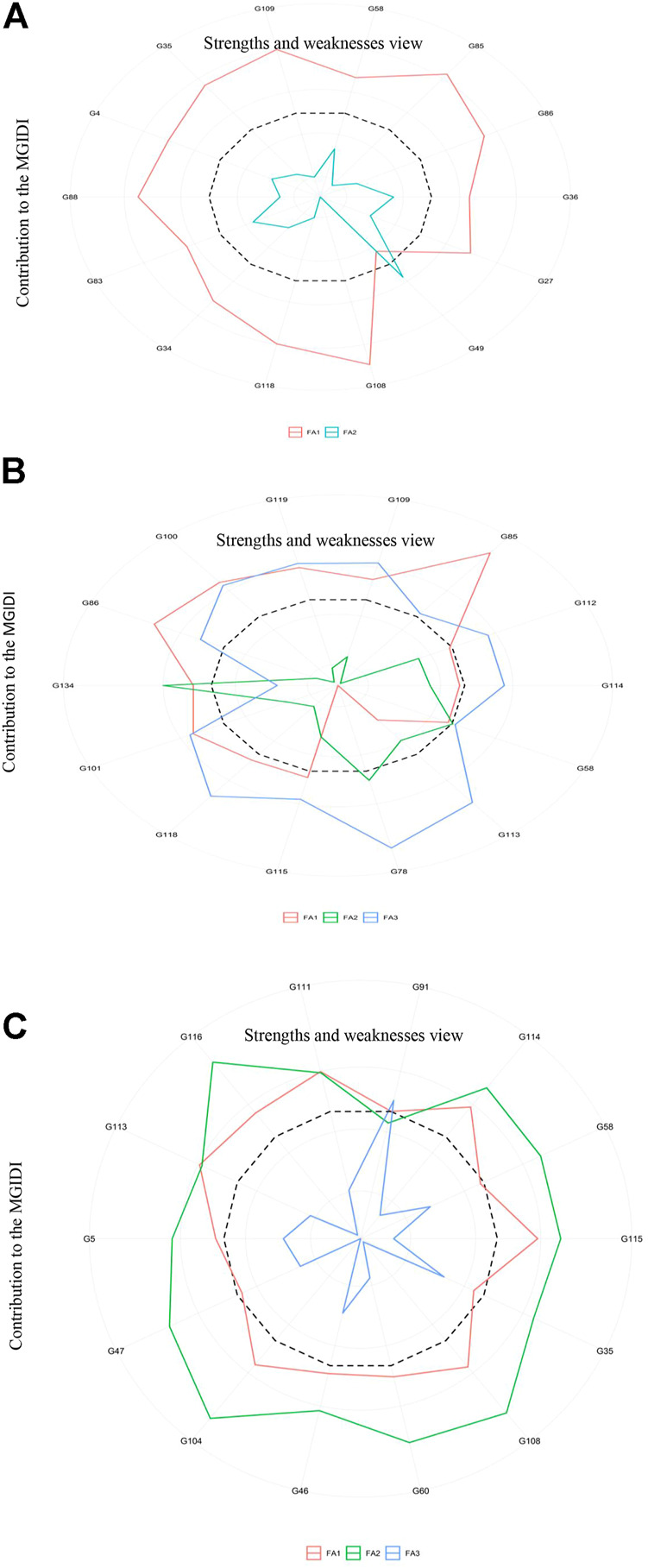
Strengths and weaknesses of the stable genotypes identified in the control **(A),** S1 (150 mM) **(B)**, and S2 (250 mM) **(C)** groups.

PCA analysis disentangles a large data set into a small number of unrelated groups that can easily be plotted along independent linear axes. Closely linked variables in the same group may hint at latent relationships among them. Multiple traits often make it difficult to choose the best-performing genotypes. Therefore, several counter multivariate approaches such as cluster analysis, factor analysis, and PCA including other indices (Hazel, 1943; Williams, 1962) have been devised; however, each has limitations. We applied the MGIDI index for each treatment to identify superior genotypes. The analysis identified ten genotypes for further assessments; among these ten genotypes, only one was common to all three treatments. G58 showed the best ideotype, with positive genetic gains for all traits (Figure 7). MGIDI is a novel way to select genotypes. Other researchers have used this approach in different crops including strawberry, wheat, barley, guar, and soybean (Gabriel et al., 2019; Olivoto et al., 2021; Lima et al.; Farhad et al., 2022; Pour-Aboughadareh et al., 2021; Benakanahalli et al., 2021; Maranna et al., 2021).

**FIGURE 8:**
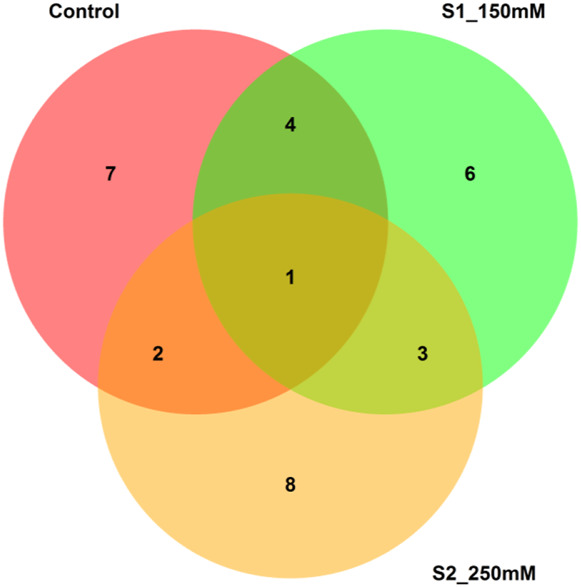
Venn diagram of the common genotypes shared across the treatments.

According to the IMGIDI-based selection of genotypes, we identified the 10 best accessions (58, 85, 86, 108, 118, 35, 109, 113, 115, and 114) for all treatments (Figure 8). We observed the genotypic profile of these genotypes with respect to the highly significant MTAs discussed above. The identified genotypes carried from 37 to 44 positive alleles out of 48 possible positive alleles, with accessions 109 (GID: 7642809) and 115 (GID: 7642901) carrying the maximum numbers (44) of positive alleles. The phenotypic profiles of these accessions under both salinity levels (150 and 250 mM NaCl) indicated a percent increase in all traits except RN_S0, Sl_S0, RL_S0, R/S_S0, and SL_S2. After excluding these traits, all traits showed a mean increase of approximately 9.9% from the population mean (Supplementary Table S6). The use of accessions with more favorable alleles in wheat breeding can aid in improving salinity tolerance traits.

## Conclusion

This study comprehensively dissected the performance of diverse bread wheat germplasm against different levels of salt stress. A total of 138 lines were screened at the seedling stage for seven traits at 0, 150, and 250 mM NaCl. We identified 195 significant SNPs/loci and 63 highly significant loci related to different traits. Most of the associations were present on the A genome, especially on chromosome 2A, and strengthened our findings regarding salinity tolerance. A total of 12 candidate genes were associated with highly significant SNP markers. The chromosomal localization of many of the important candidate genes such as Plastidial GAPA1, Metacaspase-1, etc., and their role in salt stress were also reported previously. These results of the extensive study of salt stress tolerance in *Triticum aestivum* L. could be a valuable reference for future studies. The best-performing lines with desirable allele combinations can be incorporated into wheat breeding programs.

## Data Availability

The original contributions presented in the study are included in the article/Supplementary Material, and further inquiries can be directed to the corresponding authors.

## Author’s note

This experiment/research paper is a part of Saba Akram’s PhD study.
