# Supplementary material for: A GBS-based genome-wide association study reveals the genetic basis of salinity tolerance at the seedling stage in bread wheat (Triticum aestivum L.)
Source: Front Genet. 2022 Sep 27;13:997901. doi: 10.3389/fgene.2022.997901 (PMC9551609; doi:10.3389/fgene.2022.997901)
Supplement: Supplementary file 1 [file Table5.pdf]

Supplementary Table S5 Factors linked to correlated traits, selection differential, heritability, selection gains and objectives.

Variables	Factor	SD	SD%	h^2	SG	SG%	objective
**Control-S0**							
TG	FA1	2.20	2.42	0.98	2.17	2.38	increase
RL	FA1	1.81	19.38	1.00	1.81	19.37	increase
R_S	FA1	0.03	2.83	1.00	0.03	2.83	increase
SVI	FA1	357.22	20.87	0.99	354.98	20.74	increase
RN	FA2	0.17	3.63	0.73	0.12	2.66	increase
CL	FA2	0.27	9.77	0.98	0.26	9.56	increase
SL	FA2	1.62	17.24	1.00	1.62	17.22	increase
**150 mM-S1**							
TG	FA1	14.65	21.28	0.97	14.27	20.73	increase
CL	FA1	0.35	11.94	0.79	0.28	9.42	increase
SL	FA1	0.66	10.58	0.74	0.49	7.81	increase
SVI	FA1	404.42	46.11	0.96	387.95	44.23	increase
RN	FA2	0.18	3.49	0.41	0.07	1.42	increase
RL	FA3	1.86	30.63	0.91	1.70	27.91	increase
R_S	FA3	0.15	15.84	0.88	0.14	13.89	increase
**250 mM-S2**							
TG	FA1	21.70	40.77	0.95	20.59	38.69	increase
RL	FA1	0.98	30.01	0.82	0.80	24.67	increase
R_S	FA1	0.07	6.30	0.81	0.06	5.10	increase
SVI	FA1	262.15	74.32	0.94	245.55	69.61	increase
CL	FA2	0.25	10.52	0.76	0.19	7.99	increase
SL	FA2	0.64	21.06	0.82	0.52	17.21	increase
RN	FA3	0.23	4.48	0.51	0.12	2.28	increase

SD, selection differential; SG, selection gains; h^2, heritability.

TG, Total germination percentage; RN, Numbers of roots; CL, Coleoptile length; SL, Shoot length; RL, Roots length; R/S, Root to shoot length ratio; SVI, Seedling vigor index.
