# Supplementary material for: A GBS-based genome-wide association study reveals the genetic basis of salinity tolerance at the seedling stage in bread wheat (Triticum aestivum L.)
Source: Front Genet. 2022 Sep 27;13:997901. doi: 10.3389/fgene.2022.997901 (PMC9551609; doi:10.3389/fgene.2022.997901)
Supplement: Supplementary file 2 [file Table4.pdf]

Supplementary Table S4. Correlations among the traits measured in So, S1 and S2. Only significant correlations at *p*-value 0.05 (*) and 0.001 (**) are shown

	TG_So	RN_So	CL_So	SL_So	RL_So	R/S_So	SVI_So	TG_S1	RN_S1	CL_S1	SL_S1	RL_S1	R/S_S1	SVI_S1
TG_So	1													
RN_So		1												
CL_So		0.20*	1											
SL_So	-0.22**	0.26**	0.27**	1										
RL_So	0.28**		0.22**	0.21*	1									
R/S_So				-0.19*		1								
SVI_So	0.67**		0.27**	0.36**	0.82**	-0.17*	1							
TG_S1	0.56**				0.21*		0.42**	1						
RN_S1		0.20*							1					
CL_S1	0.18*	0.28**	0.34**		0.19*	0.18*	0.28**	0.38**		1				
SL_S1	0.27**	0.24**	0.18*	0.18*	0.29**		0.40**	0.46**		0.58**	1			
RL_S1	0.40**	0.18*		0.18*	0.50**		0.59**	0.58**		0.54**	0.63**	1		
R/S_S1	0.32**				0.41**		0.46**	0.41**		0.30**		0.83**	1	
SVI_S1	0.54**				0.38**		0.56**	0.91**		0.52**	0.68**	0.83**	0.57**	1

Supplementary Table S4 Conti…

	TG_So	RN_So	CL_So	SL_So	RL_So	R/S_So	SVI_So	TG_S2	RN_S2	CL_S2	SL_S2	RL_S2	R/S_S2	SVI_S2
TG_So	1													
RN_So		1												
CL_So		0.20*	1											
SL_So	-0.22**	0.26**	0.27**	1										
RL_So	0.28**		0.22**	0.21*	1									
R/S_So				-0.19*		1								
SVI_So	0.67**		0.27**	0.36**	0.82**	-0.17*	1							
TG_S2	0.55**				0.25**		0.44**	1						
RN_S2		0.30**							1					
CL_S2				-0.20**				0.28**		1				
SL_S2										0.65**	1			
RL_S2	0.38**				0.38**		0.39**	0.58**		0.60**	0.47**	1		
R/S_S2	0.34**				0.31**		0.37**	0.49**			-0.36**	0.60**	1	
SVI_S2	0.49**				0.33**		0.43**	0.88**		0.57**	0.50**	0.84**	0.43**	1

Supplementary Table S4 Conti…

	TG_S1	RN_S1	CL_S1	SL_S1	RL_S1	R/S_S1	SVI_S1	TG_S2	RN_S2	CL_S2	SL_S2	RL_S2	R/S_S2	SVI_S2
TG_S1	1													
RN_S1		1												
CL_S1	0.38**		1											
SL_S1	0.46**		0.58**	1										
RL_S1	0.58**		0.54**	0.63**	1									
R/S_S1	0.41**		0.30**		0.83**	1								
SVI_S1	0.91**		0.52**	0.68**	0.83**	0.57**	1							

TG_S2	0.89**		0.34**	0.43**	0.62**	0.48**	0.86**	1						
RN_S2		0.24**		0.21*					1					
CL_S2	0.22**							0.28**		1				
SL_S2			-0.26**	-0.21**	-0.20**					0.65**	1			
RL_S2	0.49**		0.19**		0.48**	0.56**	0.51**	0.58**		0.60**	0.47**	1		
R/S_S2	0.49**		0.43**	0.27**	0.68**	0.65**	0.60**	0.49**			-0.36**	0.60**	1	
SVI_S2	0.76**		0.25**	0.28**	0.55**	0.49**	0.73**	0.88**		0.57**	0.50**	0.84**	0.43**	1

Supplementary Table S4 Conti…

	RTG_S1	RRN_S1	RCL_S1	RSL_S1	RRL_S1	RR/S_S1	RSVI_S1	RTG_S2	RRN_S2	RCL_S2	RSL_S2	RRL_S2	RR/S_S2	RSVI_S2
RTG_S1	1													
RRN_S1		1												
RCL_S1	0.39**	-0.20*	1											
RSL_S1	0.38**		0.44**	1										
RRL_S1	0.47**		0.52**		1									
RR/S_S1	0.17*	-0.19*	-0.22*	-0.31**	0.70**	1								
RSVI_S1	0.90**		0.54**	0.62**	0.75**	0.27**	1							

RTG_S2	0.85**		0.34**	0.34**	0.51**	0.24**	0.82**	1						
RRN_S2		0.67**				-0.20*		0	1					
RCL_S2	0.21*		0.29**				0.20*	0.27**	0	1				
RSL_S2		0.24**			-0.23**	-0.33**			0.23**	0.66**	1			
RRL_S2	0.34*		0.22**		0.42**	0.29**	0.41**	0.43**		0.57**	0.53**	1		
RR/S_S2	0.32**		0.35**		0.64**	0.66**	0.42**	0.33**	-0.17*		0.44**	0.46**	1	
RSVI_S2	0.67**		0.29**	0.33**	0.43**		0.68**	0.83**		0.60**	0.55**	0.78**	0.22**	1
