# Supplementary material for: A GBS-based genome-wide association study reveals the genetic basis of salinity tolerance at the seedling stage in bread wheat (Triticum aestivum L.)
Source: Front Genet. 2022 Sep 27;13:997901. doi: 10.3389/fgene.2022.997901 (PMC9551609; doi:10.3389/fgene.2022.997901)
Supplement: Supplementary file 3 [file Table1.pdf]

Supplementary Table S1 Pedigree of germplasm

Sr. No.	Genotype	GID Number	Pedigree	Q Group
1	SEEDLTPP2	7640766	CETA/AE.SQUARROSA (665)//KACHU/3/BAJ #1	Q1
2	SEEDLTPP59	7640823	KE90-282/MILAN//KACHU/3/BAJ #1	
3	SEEDLTPP69	7640833	KE90-282/MILAN//KACHU/3/BAJ #1	Q3
4	SEEDLTPP90	7640854	KE90-282/MILAN//KACHU/3/BAJ #1	Q1
5	SEEDLTPP138	7640902	IG 41485/KACHU//BAJ #1	Q3
6	SEEDLTPP176	7640940	IG 42158/KACHU//BAJ #1	Q1
7	SEEDLTPP2437	7640963	SCOOP_1/AE.SQUARROSA (634)//KACHU/3/BAJ #1	Q1
8	SEEDLTPP2446	7641255	TK SN1081/AE.SQUARROSA (690)//KACHU/3/BAJ #1	Q1
9	SEEDLTPP2450	7641281	GAN/AE.SQUARROSA (897)//KACHU/3/BAJ #1	Q3
10	SEEDLTPP2456	7641318	CROC_1/AE.SQUARROSA (516)//KACHU/3/BAJ #1	Q1
11	SEEDLTPP2457	7641324	CROC_1/AE.SQUARROSA (516)//KACHU/3/BAJ #1	Q3
12	SEEDLTPP2460	7641333	CROC_1/AE.SQUARROSA (516)//KACHU/3/BAJ #1	Q3
13	SEEDLTPP2463	7641340	CROC_1/AE.SQUARROSA (517)//KACHU/3/BAJ #1	Q1
14	SEEDLTPP2464	7641343	CROC_1/AE.SQUARROSA (517)//KACHU/3/BAJ #1	
15	SEEDLTPP2486	7641405	DOY1/AE.SQUARROSA (1024)//KACHU/3/BAJ #1	Q1
16	SEEDLTPP2	76414	DOY1/AE.SQUARROSA (1024)//KACHU/3/BAJ #1	Q1

	SEEDLTPP2 487	7641406		
17	SEEDLTPP2 489	7641408	DOY1/AE.SQUARROSA (1024)//KACHU/3/BAJ #1	Q3
18	SEEDLTPP2 491	7641418	CETA/AE.SQUARROSA (1025)//KACHU/3/BAJ #1	Q1
19	SEEDLTPP2 508	7641461	ARLIN_1/AE.SQUARROSA (1017)//KACHU/3/BAJ #1	Q1
20	SEEDLTPP2 520	7641563	D67.2/PARANA 66.270//AE.SQUARROSA (185)/3/KACHU/4/BAJ #1	Q3
21	SEEDLTPP1 95	7641608	T.DICOCCON PI347230/AE.SQUARROSA (879)//KACHU/3/BAJ #1	Q1
22	SEEDLTPP2 524	7641628	GAN/AE.SQUARROSA (680)//KACHU/3/BAJ #1	Q1
23	SEEDLTPP2 534	7641701	CROC_1/AE.SQUARROSA (298)//KACHU/3/BAJ #1	Q1
24	SEEDLTPP2 539	7641715	CROC_1/AE.SQUARROSA (298)//KACHU/3/BAJ #1	Q1
25	SEEDLTPP2 16	7641741	DVERD_2/T.URARTU (545)//KACHU/3/BAJ #1	Q1
26	SEEDLTPP2 549	7641789	IG 42153/KACHU//BAJ #1	Q1
27	SEEDLTPP2 550	7641791	IG 42153/KACHU//BAJ #1	Q1
28	SEEDLTPP2 42	7641797	H-1442/KACHU//BAJ #1	Q3
29	SEEDLTPP2 57	7641812	H-1442/KACHU//BAJ #1	Q1
30	SEEDLTPP2 58	7641813	H-1442/KACHU//BAJ #1	Q1
31	SEEDLTPP2 59	7641814	H-1442/KACHU//BAJ #1	Q1
32	SEEDLTPP2 70	7641825	H-1442/KACHU//BAJ #1	Q1
33	SEEDLTPP2 77	7641832	H-1442/KACHU//BAJ #1	Q1
34	SEEDLTPP2 79	7641834	H-1442/KACHU//BAJ #1	Q1

35	SEEDLTPP290	7641845	W47/KACHU//BAJ #1	Q1
36	SEEDLTPP303	7641858	W47/KACHU//BAJ #1	Q3
37	SEEDLTPP304	7641859	W47/KACHU//BAJ #1	Q3
38	SEEDLTPP305	7641860	W47/KACHU//BAJ #1	Q3
39	SEEDLTPP362	7641917	CHEN/AE.SQ//2*OPATA/3/BAJ #1/4/SUP152	Q3
40	SEEDLTPP363	7641918	CHEN/AE.SQ//2*OPATA/3/BAJ #1/4/SUP152	Q3
41	SEEDLTPP366	7641921	CHEN/AE.SQ//2*OPATA/3/BAJ #1/4/SUP152	Q5
42	SEEDLTPP371	7641926	CHEN/AE.SQ//2*OPATA/3/BAJ #1/4/SUP152	Q5
43	SEEDLTPP375	7641930	CHEN/AE.SQ//2*OPATA/3/BAJ #1/4/SUP152	Q5
44	SEEDLTPP386	7641941	CHEN/AE.SQ//2*OPATA/3/BAJ #1/4/SUP152	Q5
45	SEEDLTPP404	7641959	CHEN/AE.SQ//2*OPATA/3/BAJ #1/4/SUP152	Q5
46	SEEDLTPP407	7641962	CHEN/AE.SQ//2*OPATA/3/BAJ #1/4/SUP152	Q5
47	SEEDLTPP411	7641966	CHEN/AE.SQ//2*OPATA/3/BAJ #1/4/SUP152	Q5
48	SEEDLTPP412	7641967	CHEN/AE.SQ//2*OPATA/3/BAJ #1/4/SUP152	Q5
49	SEEDLTPP415	7641970	CHEN/AE.SQ//2*OPATA/3/BAJ #1/4/SUP152	
50	SEEDLTPP416	7641971	CHEN/AE.SQ//2*OPATA/3/BAJ #1/4/SUP152	Q5
51	SEEDLTPP418	7641973	CHEN/AE.SQ//2*OPATA/3/BAJ #1/4/SUP152	Q5
52	SEEDLTPP420	7641975	CHEN/AE.SQ//2*OPATA/3/BAJ #1/4/SUP152	Q5
53	SEEDLTPP2	76419	IG 42161/BAJ #1//SUP152	Q5

	551	99		
54	SEEDLTPP2 552	7642000	IG 42161/BAJ #1//SUP152	Q5
55	SEEDLTPP2 553	7642005	IG 42161/BAJ #1//SUP152	Q5
56	SEEDLTPP2 562	7642080	D67.2/PARANA 66.270//AE.SQUARROSA (320)/3/BAJ #1/4/SUP152	Q3
57	SEEDLTPP2 564	7642087	D67.2/PARANA 66.270//AE.SQUARROSA (643)/3/BAJ #1/4/SUP152	Q5
58	SEEDLTPP2 567	7642123	CETA/AE.SQUARROSA (409)//BAJ #1/3/SUP152	Q5
59	SEEDLTPP2 569	7642161	DOY1/AE.SQUARROSA (264)//BAJ #1/3/SUP152	Q5
60	SEEDLTPP2 571	7642168	DOY1/AE.SQUARROSA (264)//BAJ #1/3/SUP152	Q5
61	SEEDLTPP2 572	7642171	DOY1/AE.SQUARROSA (264)//BAJ #1/3/SUP152	Q1
62	SEEDLTPP2 592	7642284	BCN/4/RABI//GS/CRA/3/AE.SQUARROSA (895)/5/SUP152/6/VILLA JUAREZ F2009	Q3
63	SEEDLTPP2 594	7642291	BCN/4/RABI//GS/CRA/3/AE.SQUARROSA (895)/5/SUP152/6/VILLA JUAREZ F2009	Q3
64	SEEDLTPP2 595	7642292	BCN/4/RABI//GS/CRA/3/AE.SQUARROSA (895)/5/SUP152/6/VILLA JUAREZ F2009	Q3
65	SEEDLTPP2 600	7642310	BCN/4/RABI//GS/CRA/3/AE.SQUARROSA (895)/5/SUP152/6/VILLA JUAREZ F2009	
66	SEEDLTPP2 602	7642323	BCN/4/RABI//GS/CRA/3/AE.SQUARROSA (895)/5/SUP152/6/VILLA JUAREZ F2009	Q4
67	SEEDLTPP2 603	7642324	BCN/4/RABI//GS/CRA/3/AE.SQUARROSA (895)/5/SUP152/6/VILLA JUAREZ F2009	Q4
68	SEEDLTPP2 608	7642370	SORA/AE.SQUARROSA (442)//SUP152/3/VILLA JUAREZ F2009	Q4
69	SEEDLTPP2 610	7642372	SORA/AE.SQUARROSA (442)//SUP152/3/VILLA JUAREZ F2009	Q4
70	SEEDLTPP2 611	7642377	SORA/AE.SQUARROSA (442)//SUP152/3/VILLA JUAREZ F2009	Q4
71	SEEDLTPP2 612	7642378	SORA/AE.SQUARROSA (442)//SUP152/3/VILLA JUAREZ F2009	Q4

72	SEEDLTPP2 613	76423 79	SORA/AE.SQUARROSA (442)//SUP152/3/VILLA JUAREZ F2009	Q4
73	SEEDLTPP2 617	76424 10	IG 122628/SUP152//VILLA JUAREZ F2009	Q4
74	SEEDLTPP2 623	76424 36	BCN//SORA/AE.SQUARROSA (323)/3/VILLA JUAREZ F2009/4/WBLL1/KUKUNA//TACUPETO F2001/3/BAJ #1	Q4
75	SEEDLTPP2 624	76424 43	BCN//SORA/AE.SQUARROSA (323)/3/VILLA JUAREZ F2009/4/WBLL1/KUKUNA//TACUPETO F2001/3/BAJ #1	Q4
76	SEEDLTPP4 40	76424 47	BCN//CETA/AE.SEARSII (34D)/3/VILLA JUAREZ F2009/4/WBLL1/KUKUNA//TACUPETO F2001/3/BAJ #1	Q4
77	SEEDLTPP4 49	76424 56	BCN//CETA/AE.SEARSII (34D)/3/VILLA JUAREZ F2009/4/WBLL1/KUKUNA//TACUPETO F2001/3/BAJ #1	Q4
78	SEEDLTPP4 57	76424 64	BCN//CETA/AE.SEARSII (34D)/3/VILLA JUAREZ F2009/4/WBLL1/KUKUNA//TACUPETO F2001/3/BAJ #1	
79	SEEDLTPP4 63	76424 70	BCN//CETA/AE.SEARSII (34D)/3/VILLA JUAREZ F2009/4/WBLL1/KUKUNA//TACUPETO F2001/3/BAJ #1	Q4
80	SEEDLTPP4 69	76424 76	BCN//CETA/AE.SEARSII (34D)/3/VILLA JUAREZ F2009/4/WBLL1/KUKUNA//TACUPETO F2001/3/BAJ #1	Q4
81	SEEDLTPP4 70	76424 77	BCN//CETA/AE.SEARSII (34D)/3/VILLA JUAREZ F2009/4/WBLL1/KUKUNA//TACUPETO F2001/3/BAJ #1	Q4
82	SEEDLTPP2 628	76424 98	CHEN/AE.SQ//WEAVER/3/VILLA JUAREZ F2009/4/WBLL1/KUKUNA//TACUPETO F2001/3/BAJ #1	Q4
83	SEEDLTPP2 637	76425 79	IG 42157/VILLA JUAREZ F2009/4/WBLL1/KUKUNA//TACUPETO F2001/3/BAJ #1	Q4
84	SEEDLTPP2 648	76426 24	IG 122793/VILLA JUAREZ F2009/4/WBLL1/KUKUNA//TACUPETO F2001/3/BAJ #1	Q4
85	SEEDLTPP2 649	76426 27	IG 122793/VILLA JUAREZ F2009/4/WBLL1/KUKUNA//TACUPETO F2001/3/BAJ #1	Q4
86	SEEDLTPP2 652	76426 41	IG 126482/VILLA JUAREZ F2009/4/WBLL1/KUKUNA//TACUPETO F2001/3/BAJ #1	Q4
87	SEEDLTPP4 93	76426 53	BCN//CETA/AE.SEARSII (34D)/4/WBLL1/KUKUNA//TACUPETO F2001/3/BAJ #1/5/SERI.1B//KAUZ/HEVO/3/AMAD*2/4/KIRITATI	Q4
88	SEEDLTPP4 97	76426 57	BCN//CETA/AE.SEARSII (34D)/4/WBLL1/KUKUNA//TACUPETO F2001/3/BAJ #1/5/SERI.1B//KAUZ/HEVO/3/AMAD*2/4/KIRITATI	Q4
89	SEEDLTPP4 98	76426 58	BCN//CETA/AE.SEARSII (34D)/4/WBLL1/KUKUNA//TACUPETO F2001/3/BAJ #1/5/SERI.1B//KAUZ/HEVO/3/AMAD*2/4/KIRITATI	Q4
90	SEEDLTPP4	76426	BCN//CETA/AE.SEARSII (34D)/4/WBLL1/KUKUNA//TACUPETO F2001/3/BAJ #1/5/SERI.1B//KAUZ/HEVO/3/AMAD*2/4/KIRITATI	Q4

	99	59		
91	SEEDLTPP502	7642662	BCN//CETA/AE.SEARSII (34D)/4/WBLL1/KUKUNA//TACUPETO F2001/3/BAJ #1/5/SERI.1B//KAUZ/HEVO/3/AMAD*2/4/KIRITATI	Q4
92	SEEDLTPP536	7642696	BCN//SORA/AE.SQUARROSA (323)/4/WBLL1/KUKUNA//TACUPETO F2001/3/BAJ #1/5/SERI.1B//KAUZ/HEVO/3/AMAD*2/4/KIRITATI	Q2
93	SEEDLTPP542	7642702	BCN//SORA/AE.SQUARROSA (323)/4/WBLL1/KUKUNA//TACUPETO F2001/3/BAJ #1/5/SERI.1B//KAUZ/HEVO/3/AMAD*2/4/KIRITATI	Q2
94	SEEDLTPP544	7642704	BCN//SORA/AE.SQUARROSA (323)/4/WBLL1/KUKUNA//TACUPETO F2001/3/BAJ #1/5/SERI.1B//KAUZ/HEVO/3/AMAD*2/4/KIRITATI	Q2
95	SEEDLTPP545	7642705	BCN//SORA/AE.SQUARROSA (323)/4/WBLL1/KUKUNA//TACUPETO F2001/3/BAJ #1/5/SERI.1B//KAUZ/HEVO/3/AMAD*2/4/KIRITATI	Q2
96	SEEDLTPP549	7642709	BCN//SORA/AE.SQUARROSA (323)/4/WBLL1/KUKUNA//TACUPETO F2001/3/BAJ #1/5/SERI.1B//KAUZ/HEVO/3/AMAD*2/4/KIRITATI	Q2
97	SEEDLTPP550	7642710	BCN//SORA/AE.SQUARROSA (323)/4/WBLL1/KUKUNA//TACUPETO F2001/3/BAJ #1/5/SERI.1B//KAUZ/HEVO/3/AMAD*2/4/KIRITATI	Q2
98	SEEDLTPP553	7642713	BCN//SORA/AE.SQUARROSA (323)/4/WBLL1/KUKUNA//TACUPETO F2001/3/BAJ #1/5/SERI.1B//KAUZ/HEVO/3/AMAD*2/4/KIRITATI	Q2
99	SEEDLTPP561	7642721	BCN//SORA/AE.SQUARROSA (323)/4/WBLL1/KUKUNA//TACUPETO F2001/3/BAJ #1/5/SERI.1B//KAUZ/HEVO/3/AMAD*2/4/KIRITATI	Q2
100	SEEDLTPP563	7642723	BCN//SORA/AE.SQUARROSA (323)/4/WBLL1/KUKUNA//TACUPETO F2001/3/BAJ #1/5/SERI.1B//KAUZ/HEVO/3/AMAD*2/4/KIRITATI	Q2
101	SEEDLTPP564	7642724	BCN//SORA/AE.SQUARROSA (323)/4/WBLL1/KUKUNA//TACUPETO F2001/3/BAJ #1/5/SERI.1B//KAUZ/HEVO/3/AMAD*2/4/KIRITATI	Q2
102	SEEDLTPP573	7642733	CHEN/AE.SQ//2*OPATA/4/WBLL1/KUKUNA//TACUPETO F2001/3/BAJ #1/5/SERI.1B//KAUZ/HEVO/3/AMAD*2/4/KIRITATI	Q2
103	SEEDLTPP588	7642748	CHEN/AE.SQ//2*OPATA/4/WBLL1/KUKUNA//TACUPETO F2001/3/BAJ #1/5/SERI.1B//KAUZ/HEVO/3/AMAD*2/4/KIRITATI	Q2
104	SEEDLTPP594	7642754	CHEN/AE.SQ//2*OPATA/4/WBLL1/KUKUNA//TACUPETO F2001/3/BAJ #1/5/SERI.1B//KAUZ/HEVO/3/AMAD*2/4/KIRITATI	Q2
105	SEEDLTPP611	7642771	ARVAND 1/4/WBLL1/KUKUNA//TACUPETO F2001/3/BAJ #1/5/SERI.1B//KAUZ/HEVO/3/AMAD*2/4/KIRITATI	Q2
106	SEEDLTPP617	7642777	ARVAND 1/4/WBLL1/KUKUNA//TACUPETO F2001/3/BAJ #1/5/SERI.1B//KAUZ/HEVO/3/AMAD*2/4/KIRITATI	Q2
107	SEEDLTPP621	7642781	ARVAND 1/4/WBLL1/KUKUNA//TACUPETO F2001/3/BAJ #1/5/SERI.1B//KAUZ/HEVO/3/AMAD*2/4/KIRITATI	Q2
108	SEEDLTPP626	7642786	ARVAND 1/4/WBLL1/KUKUNA//TACUPETO F2001/3/BAJ #1/5/SERI.1B//KAUZ/HEVO/3/AMAD*2/4/KIRITATI	Q2

109	SEEDLTPP2 655	7642809	ARLIN_1/AE.SQUARROSA (536)/4/WBLL1/KUKUNA//TACUPETO F2001/3/BAJ #1/5/SERI.1B//KAUZ/HEVO/3/AMAD*2/4/KIRITATI	Q2
110	SEEDLTPP2 656	7642821	ARLIN_1/AE.SQUARROSA (536)/4/WBLL1/KUKUNA//TACUPETO F2001/3/BAJ #1/5/SERI.1B//KAUZ/HEVO/3/AMAD*2/4/KIRITATI	Q2
111	SEEDLTPP2 659	7642850	CETA/AE.SQUARROSA (1036)/4/WBLL1/KUKUNA//TACUPETO F2001/3/BAJ #1/5/SERI.1B//KAUZ/HEVO/3/AMAD*2/4/KIRITATI	Q2
112	SEEDLTPP2 660	7642860	CETA/AE.SQUARROSA (1036)/4/WBLL1/KUKUNA//TACUPETO F2001/3/BAJ #1/5/SERI.1B//KAUZ/HEVO/3/AMAD*2/4/KIRITATI	Q2
113	SEEDLTPP2 664	7642888	CPI8/GEDIZ/3/GOO//ALB/CRA/4/AE.SQUARROSA (1038)/5/WBLL1/KUKUNA//TACUPETO F2001/3/BAJ #1/6/SERI.1B//KAUZ/HEVO/3/AMAD*2/4/KIRITATI	Q2
114	SEEDLTPP2 665	7642891	CPI8/GEDIZ/3/GOO//ALB/CRA/4/AE.SQUARROSA (1038)/5/WBLL1/KUKUNA//TACUPETO F2001/3/BAJ #1/6/SERI.1B//KAUZ/HEVO/3/AMAD*2/4/KIRITATI	
115	SEEDLTPP2 666	7642901	D67.2/PARANA 66.270//AE.SQUARROSA (828)/4/WBLL1/KUKUNA//TACUPETO F2001/3/BAJ #1/5/SERI.1B//KAUZ/HEVO/3/AMAD*2/4/KIRITATI	Q2
116	SEEDLTPP2 667	7642903	D67.2/PARANA 66.270//AE.SQUARROSA (828)/4/WBLL1/KUKUNA//TACUPETO F2001/3/BAJ #1/5/SERI.1B//KAUZ/HEVO/3/AMAD*2/4/KIRITATI	Q2
117	SEEDLTPP2 670	7642906	D67.2/PARANA 66.270//AE.SQUARROSA (828)/4/WBLL1/KUKUNA//TACUPETO F2001/3/BAJ #1/5/SERI.1B//KAUZ/HEVO/3/AMAD*2/4/KIRITATI	Q2
118	SEEDLTPP2 671	7642907	D67.2/PARANA 66.270//AE.SQUARROSA (828)/4/WBLL1/KUKUNA//TACUPETO F2001/3/BAJ #1/5/SERI.1B//KAUZ/HEVO/3/AMAD*2/4/KIRITATI	Q2
119	SEEDLTPP2 674	7642911	D67.2/PARANA 66.270//AE.SQUARROSA (828)/4/WBLL1/KUKUNA//TACUPETO F2001/3/BAJ #1/5/SERI.1B//KAUZ/HEVO/3/AMAD*2/4/KIRITATI	Q2
120	SEEDLTPP2 675	7642917	D67.2/PARANA 66.270//AE.SQUARROSA (828)/4/WBLL1/KUKUNA//TACUPETO F2001/3/BAJ #1/5/SERI.1B//KAUZ/HEVO/3/AMAD*2/4/KIRITATI	Q2
121	SEEDLTPP2 680	7642934	GAN/AE.SQUARROSA (741)/4/WBLL1/KUKUNA//TACUPETO F2001/3/BAJ #1/5/SERI.1B//KAUZ/HEVO/3/AMAD*2/4/KIRITATI	Q2
122	SEEDLTPP2 684	7642941	GAN/AE.SQUARROSA (741)/4/WBLL1/KUKUNA//TACUPETO F2001/3/BAJ #1/5/SERI.1B//KAUZ/HEVO/3/AMAD*2/4/KIRITATI	Q2
123	SEEDLTPP2 689	7642976	68.111/RGB-U//WARD RESEL/3/STIL/4/AE.SQUARROSA (188)/5/WBLL1/KUKUNA//TACUPETO F2001/3/BAJ #1/6/SERI.1B//KAUZ/HEVO/3/AMAD*2/4/KIRITATI	Q2
124	SEEDLTPP2 690	7642977	68.111/RGB-U//WARD RESEL/3/STIL/4/AE.SQUARROSA (188)/5/WBLL1/KUKUNA//TACUPETO F2001/3/BAJ #1/6/SERI.1B//KAUZ/HEVO/3/AMAD*2/4/KIRITATI	Q2
125	SEEDLTPP2 715	7643065	IWA8611400/4/WBLL1/KUKUNA//TACUPETO F2001/3/BAJ #1/5/SERI.1B//KAUZ/HEVO/3/AMAD*2/4/KIRITATI	Q2
126	SEEDLTPP2 717	7643067	IWA8611400/4/WBLL1/KUKUNA//TACUPETO F2001/3/BAJ #1/5/SERI.1B//KAUZ/HEVO/3/AMAD*2/4/KIRITATI	Q2
127	SEEDLTPP2	76430	CHEN/AE.SQ//2*OPATA/5/SERI.1B//KAUZ/HEVO/3/AMAD*2/4/KIRITATI/6/FRET2*2/4/SNI/TRAP#1/3/KAUZ*2/TRAP//KAUZ/5/KACHU	Q2

	718	71		
128	SEEDLTPP2 719	76430 72	CHEN/AE.SQ//2*OPATA/5/SERI.1B//KAUZ/HEVO/3/AMAD*2/4/KIRITATI/6/FRET2*2/4/SNI/TRAP#1/3/KAUZ*2/TRAP//KAUZ/5/KACHU	Q2
129	SEEDLTPP2 720	76430 73	CHEN/AE.SQ//2*OPATA/5/SERI.1B//KAUZ/HEVO/3/AMAD*2/4/KIRITATI/6/FRET2*2/4/SNI/TRAP#1/3/KAUZ*2/TRAP//KAUZ/5/KACHU	Q3
130	SEEDLTPP2 721	76430 74	CHEN/AE.SQ//2*OPATA/5/SERI.1B//KAUZ/HEVO/3/AMAD*2/4/KIRITATI/6/FRET2*2/4/SNI/TRAP#1/3/KAUZ*2/TRAP//KAUZ/5/KACHU	Q2
131	SEEDLTPP2 722	76430 76	CHEN/AE.SQ//2*OPATA/5/SERI.1B//KAUZ/HEVO/3/AMAD*2/4/KIRITATI/6/FRET2*2/4/SNI/TRAP#1/3/KAUZ*2/TRAP//KAUZ/5/KACHU	Q2
132	SEEDLTPP2 724	76430 80	CHEN/AE.SQ//2*OPATA/5/SERI.1B//KAUZ/HEVO/3/AMAD*2/4/KIRITATI/6/FRET2*2/4/SNI/TRAP#1/3/KAUZ*2/TRAP//KAUZ/5/KACHU	Q2
133	SEEDLTPP2 725	76430 81	CHEN/AE.SQ//2*OPATA/5/SERI.1B//KAUZ/HEVO/3/AMAD*2/4/KIRITATI/6/FRET2*2/4/SNI/TRAP#1/3/KAUZ*2/TRAP//KAUZ/5/KACHU	Q3
134	SEEDLTPP2 727	76430 84	CHEN/AE.SQ//2*OPATA/5/SERI.1B//KAUZ/HEVO/3/AMAD*2/4/KIRITATI/6/FRET2*2/4/SNI/TRAP#1/3/KAUZ*2/TRAP//KAUZ/5/KACHU	Q3
135	SEEDLTPP2 730	76430 90	CHEN/AE.SQ//2*OPATA/5/SERI.1B//KAUZ/HEVO/3/AMAD*2/4/KIRITATI/6/FRET2*2/4/SNI/TRAP#1/3/KAUZ*2/TRAP//KAUZ/5/KACHU	Q3
136	SEEDLTPP2 740	76431 03	CHEN/AE.SQ//2*OPATA/5/SERI.1B//KAUZ/HEVO/3/AMAD*2/4/KIRITATI/6/FRET2*2/4/SNI/TRAP#1/3/KAUZ*2/TRAP//KAUZ/5/KACHU	Q3
137	SEEDLTPP2 749	76431 21	CHEN/AE.SQ//WEAVER/5/SERI.1B//KAUZ/HEVO/3/AMAD*2/4/KIRITATI/6/FRET2*2/4/SNI/TRAP#1/3/KAUZ*2/TRAP//KAUZ/5/KACHU	Q1
138	SEEDLTPP2 752	76431 26	CHEN/AE.SQ//WEAVER/5/SERI.1B//KAUZ/HEVO/3/AMAD*2/4/KIRITATI/6/FRET2*2/4/SNI/TRAP#1/3/KAUZ*2/TRAP//KAUZ/5/KACHU	Q3

All pedigree/crosses detail has already published and available at (https://seedsofdiscovery.org/wp-content/uploads/sites/52/2017/10/7_MasAgro_Trigo_2.pdf) (Wanyera and Owuoche, 2017; Singh et al., 2018; Großkinsky et al., 2020)
