# Supplementary material for: A GBS-based genome-wide association study reveals the genetic basis of salinity tolerance at the seedling stage in bread wheat (Triticum aestivum L.)
Source: Front Genet. 2022 Sep 27;13:997901. doi: 10.3389/fgene.2022.997901 (PMC9551609; doi:10.3389/fgene.2022.997901)
Supplement: Supplementary file 4 [file Table2.pdf]

Supplementary Table S2. Range, mean±SD (standard deviation) and ANOVA of traits under control and salinity stress treatments

**Trait**	**So (Control)**		**S1 (150mM NaCl)**		**S2 (250mM NaCl)**		**ANOVA (*p*-value)**
	Range	**Mean±SD**	Range	**Mean±SD**	Range	**Mean±SD**	
TG	60-100	91.10±9.63	32-99	68.84±20.19	18-91	53.22±20.95	P_G = ***, P_T = ***, P_{G*T} = **
RN	3-6	4.52±0.44	4-6	5.13±0.31	4-8	5.12±0.45	P_G = ***, P = ***, P_{G*T} = ***
CL	2-3.8	2.76±0.27	2-4.3	2.94±0.48	1.2-3.5	2.34±0.45	P_G = ***, P_T = ***, P_{G*T} = ***
SL	6.2-17	9.38±1.28	4.1-8.8	6.24±0.95	1.4-6	3.04±0.79	P_G = *, P_T= ***
RL	4.2-13.9	9.35±1.81	2.8-10	6.08±1.79	1-6.1	3.25±1.14	P_G = ***, P_T = ***, P_{G*T} = ***
R/S	0.8-1.3	1.14±0.04	0.5-1.6	0.98±0.23	0.4-2.5	1.11±0.38	P_G = ***, P $_T$= ***, P_{G*T} = ***
SVI	1000-2327	1708±300	310-1820	877±380	64-813	153±200.76	P_G = ***, P_T = ***, P_{G*T} = ***

TG = Total germination percentage, RN =Number of roots, CL = Coleoptile length (cm), SL = Shoot length(cm), RL = Root length (cm),R/S = Root to shoot ratio and SVI = Seedling vigor index, P_G = *p* value of genotypes effect, P_T = *p* value of treatments effect, P_{G*T} = *p* value of genotypes and treatments interaction. *, ** and *** indicate significant differences at *p*-values of 0.05, 0.01 and 0.001, respectively.
