# Supplementary material for: A GBS-based genome-wide association study reveals the genetic basis of salinity tolerance at the seedling stage in bread wheat (Triticum aestivum L.)
Source: Front Genet. 2022 Sep 27;13:997901. doi: 10.3389/fgene.2022.997901 (PMC9551609; doi:10.3389/fgene.2022.997901)
Supplement: Supplementary file 5 [file Table6.pdf]

Supplementary Table S6 Allelic and phenotypic profiles of the eleven best lines performed under salt stress. Underlined and bold A, G, C and T indicate positive alleles.

rs#	alleles	Chr.	Position (Cm)	Trait	G_35	G_58	G_85	G_86	G_108	G_109	G_113	G_114	G_115	G_118
M406	C/A	1A	155.92	RL_S2	N	**C**	**C**	**C**	**C**	A	M	A	**C**	N
M1426	T/C	1B	37.65	RR/S_S1	**T**	**T**	**T**	**T**	**T**	**T**	**T**	**T**	**T**	**T**
M11993	G/A	1B	61.58	SL_S0	**G**	**G**	A	**G**	**G**	**G**	**G**	**G**	**G**	**G**
M11428	A/T	1B	384.13	SL_S0	**A**	**A**	**A**	**A**	**A**	**A**	**A**	**A**	**A**	**A**
M9978	G/T	1B	492.15	RN_S1	**G**	**G**	**G**	**G**	T	**G**	**G**	**G**	**G**	**G**
M10810	T/C	1D	22.56	RRN_S2	**T**	N	**T**	**T**	**T**	**T**	**T**	**T**	**T**	**T**
M10295	G/A	1D	90.1	SL_S0	**G**	**G**	**G**	**G**	**G**	**G**	**G**	**G**	A	**G**
M10566	T/C	1D	93.71	RR/S_S1	**T**	**T**	**T**	**T**	**T**	**T**	**T**	**T**	**T**	**T**
M8113	T/C	1D	167.7	SL_S0	**T**	T	N	N	T	T	T	T	N	**T**
M4431	A/G	2A	125.28	SL_S0, RR/S_S1	**A**	N	**A**	N	**A**	**A**	N	N	**A**	**A**
M10796	G/C	2A	159.56	RRN_S2	**G**	**G**	**G**	**G**	**G**	**G**	**G**	**G**	**G**	**G**
M765	C/A	2A	221.1	R/S_S2	**C**	A	**C**	N	**C**	**C**	**C**	**C**	A	**C**
M9176	G/A	2A	231.79	RN_S2	**G**	**G**	**G**	**G**	**G**	**G**	**G**	**G**	**G**	N

M2717	T/C	2B	**147.53**	**RR/S_S1**	T	T	T	T	T	T	T	T	T	T
M627	G/A	2D	**289.68**	**RRN_S2**	G	G	G	R	G	G	G	G	G	R
M1930	A/G	3A	**75.97**	**RN_S2**	A	G	A	A	A	A	G	G	A	A
M5307	G/A	3B	**68.2**	**RRN_S2**	G	G	G	G	G	G	G	R	G	G
M7873	T/C	3B	**103.59**	**RR/S_S1, SL_S0**	N	T	T	T	C	C	C	C	T	T
M8515	G/A	3B	**162.58**	**SL_S0,RSL_S1, RR/S_S1**	G	G	G	G	G	G	G	G	G	G
M9138	G/T	3B	**222.96**	**RR/S_S1**	G	G	T	N	G	G	G	N	G	G
M11925	T/C	3B	**227.41**	**RN_S2**	T	T	T	T	T	T	T	T	T	T
M2025	A/C	3B	**297.56**	**RN_S2**	A	A	A	C	A	A	A	A	A	A
M1987	G/A	3D	**107.75**	**RR/S_S1**	G	G	G	G	G	G	G	G	G	G
M1398	G/A	4A	**49.56**	**SL_S0**	G	G	G	G	G	G	G	G	G	G
M11711	A/G	4A	**180.05**	**SL_S0, RL_S1, RR/S_S1**	G	A	A	A	A	N	A	A	A	A
M5589	G/A	4A	**215.47**	**RN_S1**	R	G	G	A	G	G	G	G	G	G
M4103	T/C	4D	**34.78**	**RRN_S2**	T	T	T	T	T	T	T	T	T	T
M3343	T/C	4D	**50.31**	**RRN_S2**	T	T	T	T	T	T	T	T	T	T
M1974	C/T	4D	**157.4**	**RR/S_S1**	C	C	C	C	C	C	N	N	C	C
M8885	G/T	5A	**113.15**	**RR/S_S1, SL_S0**	G	T	T	G	G	G	G	G	G	N

M11486	G/A	5A	**161.09**	**RRN_S1**	A	A	G	G	G	G	G	G	G	G
M4314	A/G	5A	**209.83**	**RN_S2**	A	A	A	A	A	A	A	A	A	A
M3034	A/G	5A	**229.39**	**RN_S2**	N	A	A	A	A	A	A	A	A	A
M2139	A/G	5B	**56.97**	**RN_S2**	A	A	A	N	A	A	A	A	A	A
M4710	T/C	5B	**56.97**	**RN_S2**	T	T	T	T	T	T	T	T	T	T
M1450	T/C	5B	**125.96**	**RN_S1**	T	T	T	T	C	T	T	T	T	T
M11102	T/A	5D	**195.73**	**RN_S2**	T	T	T	N	T	T	T	T	T	T
M7775	C/G	5D	**210.31**	**RN_S2**	C	G	C	N	C	C	C	C	C	C
M337	T/C	5D	**232.48**	**RRN_S2**	T	T	T	T	T	T	T	N	T	T
M5347	C/G	6A	**65.28**	**RN_S2**	C	C	C	C	C	C	C	C	C	C
M530	C/T	6A	**73.17**	**RN_S2**	C	C	C	C	C	C	C	C	C	C
M4362	A/T	6B	**69.05**	**RR/S_S1**	A	A	A	A	A	A	A	A	A	A
M11763	T/C	6D	**90.8**	**RR/S_S1**	T	T	T	T	T	T	C	T	T	N
M1188	G/C	6D	**121.22**	**SL_S0**	G	G	S	G	G	G	S	G	S	G
M10047	T/C	7A	**45.99**	**RR/S_S1**	T	T	T	T	T	T	T	T	T	T
M9550	G/T	7A	**170.91**	**RRN_S2**	G	K	G	G	G	G	G	G	G	G
M38	G/A	7A	**214.7**	**RL_S0**	G	G	N	G	N	G	N	G	G	R
M9660	T/C	7A	**276.04**	**RN_S1**	Y	T	T	Y	C	Y	T	T	T	T
		Numbers of Positive alleles			**41**	**40**	**42**	**37**	**43**	**44**	**38**	**38**	**44**	**42**

Supplementary Table S6 Conti…..

**Trait**	G_35	G_58	G_85	G_86	G_108	G_109	G_113	G_114	G_115	G_118	sample Mean	Population mean	% increased
TG_S0	95	80	88	92	92	100	100	88	96	96	92.7±5.83	91.1±9.63	1.723115
RN_S0	4	4.5	5	4	5	4	4	4	4	4	4.25±0.40	4.52±0.44	-6.29339
CL_S0	2.5	2.9	3.1	2.9	3	2	3	2.6	2.7	2.9	2.76±0.31	2.76±0.27	-0.02951
SL_S0	7.6	8.8	10.4	9	9.2	6.9	9.1	8.9	8.3	7.6	8.58±0.96	9.38±1.28	-9.3339
RL_S0	4.2	7.5	8.9	8.2	9.5	8.1	11.9	9.1	9.6	10.3	8.73±1.92	9.35±1.81	-7.09891
R_S_S0	0.6	0.9	0.9	0.9	1	1.2	1.3	1	1.2	1.4	1.04±0.22	1.14±0.04	-9.75573
SVI_S0	1127	1305	1701	1586	1719	1503	2103	1586	1724	1718	1607.2±250.34	1708±300	-6.30285
TG_S1	46	63	68	85	86	87	97	84	48	85	74.9±16.71	68.84±20.19	8.089149
RN_S1	6	5	5	5	6	5	5	5	5	5	5.2±0.4	5.13±0.31	1.389903
CL_S1	2.5	3	3.9	3.3	3.1	2.9	3.6	3.3	3.3	3.1	3.2±0.36	2.94±0.48	8.25457
SL_S1	6.4	6.6	8.6	7.1	5.9	6.6	6.8	7.4	6.8	5.7	6.79±0.77	6.24±0.95	8.137132

RL_S1	3.9	4.7	8	7.7	8.7	6.8	7.6	7.7	5.8	5.8	6.67±1.48	6.08±1.79	8.878241
R_S_S1	0.6	0.7	0.9	1.1	1.5	1	1.1	1	0.9	1	0.98±0.23	0.98±0.23	0.520459
SVI_S1	478	711	1121	1248	1246	1167	1397	1275	608	978	1022.9±300.45	877±380	14.24965
RTG_S1	48	78	77	92	93	87	97	95	50	89	80.6±17.00	75.06±18.59	6.870025
RRN_S1	151	118	100	114	109	129	132	117	108	111	118.9±14.031749712705	114.2±12.69	3.951872
RCL_S1	99	102	125	115	104	146	120	127	121	106	116.5±13.68	106.78±16.77	8.342755
RSL_S1	85	76	83	78	64	96	74	84	82	76	79.8±7.98	67.3±11.28	15.658
RRL_S1	93	63	89	94	92	84	64	85	61	56	78.1±14.41	65.67±17.17	15.92159
RR_S_S1	111	84	108	124	146	87	86	101	74	73	99.4±22.14	99.5±28.5	-0.10097
RSVI_S1	42	54	66	79	72	78	66	80	35	57	62.9±14.87	50.77±18.27	19.28686
TG_S2	31	44	44	77	72	80	79	63	29	61	58±18.65	53.22±20.95	8.235596
RN_S2	5	5	5	7	6	5	5	5	5	5	5.3±0.64	5.12±0.45	3.31989
CL_S2	2.1	2.7	1.4	2.4	2.6	2.8	3.5	3.4	2	2.5	2.54±0.60	2.34±0.45	7.914589
SL_S2	3.3	3.2	1.5	2.6	3.1	3.7	3.6	4.7	2	2.6	3.03±0.8	3.04±0.79	-0.40069
RL_S2	2.6	3.2	2.5	3.3	4.8	5.1	5.4	5.6	2.3	3.3	3.81±1.21	3.25±1.14	14.62152

R_S_S2	0.8	1	1.7	1.3	1.5	1.4	1.5	1.2	1.1	1.3	1.28±0.25	1.10±0.38	13.22579
SVI_S2	192	271	175	455	569	704	709	645	125	362	420.7±215.13	353±200.95	16.15238
RTG_S2	33	55	50	84	78	80	79	71	30	64	62.4±18.69	57.71±20.04	7.516492
RRN_S2	121	106	106	151	107	130	118	113	108	122	118.2±13.39	114.27±12.92	3.325642
RCL_S2	85	93	46	83	88	138	116	129	74	87	93.9±25.76	85.62±18.84	8.817932
RSL_S2	43	36	14	29	34	53	39	52	24	34	35.8±11.34	33.01±9.38	7.761206
RRL_S2	62	42	28	40	50	63	46	61	24	32	44.8±13.51	35.36±12.11	21.06223
RR_S_S2	136	113	203	138	149	119	116	117	98	94	128.3±29.76	112.64±41	12.20372
RSVI_S2	17	21	10	29	33	47	34	41	7	21	26±12.39	20.38±10.55	21.60841
