# Supplementary figures and images for: A GBS-based genome-wide association study reveals the genetic basis of salinity tolerance at the seedling stage in bread wheat (Triticum aestivum L.)
Source: Front Genet. 2022 Sep 27;13:997901. doi: 10.3389/fgene.2022.997901 (PMC9551609; doi:10.3389/fgene.2022.997901)

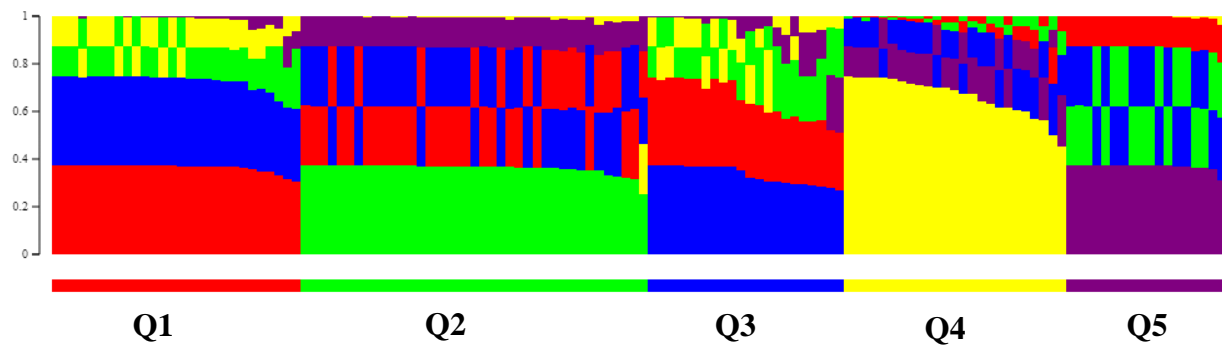

**Supplementary Figure S2** structure analysis dividing the population into five sub-group

Supplement: Supplementary file 6 [file Image2.pdf]
