# Supplementary material for: A GBS-based genome-wide association study reveals the genetic basis of salinity tolerance at the seedling stage in bread wheat (Triticum aestivum L.)
Source: Front Genet. 2022 Sep 27;13:997901. doi: 10.3389/fgene.2022.997901 (PMC9551609; doi:10.3389/fgene.2022.997901)
Supplement: Supplementary file 7 [file Image3.pdf]

A

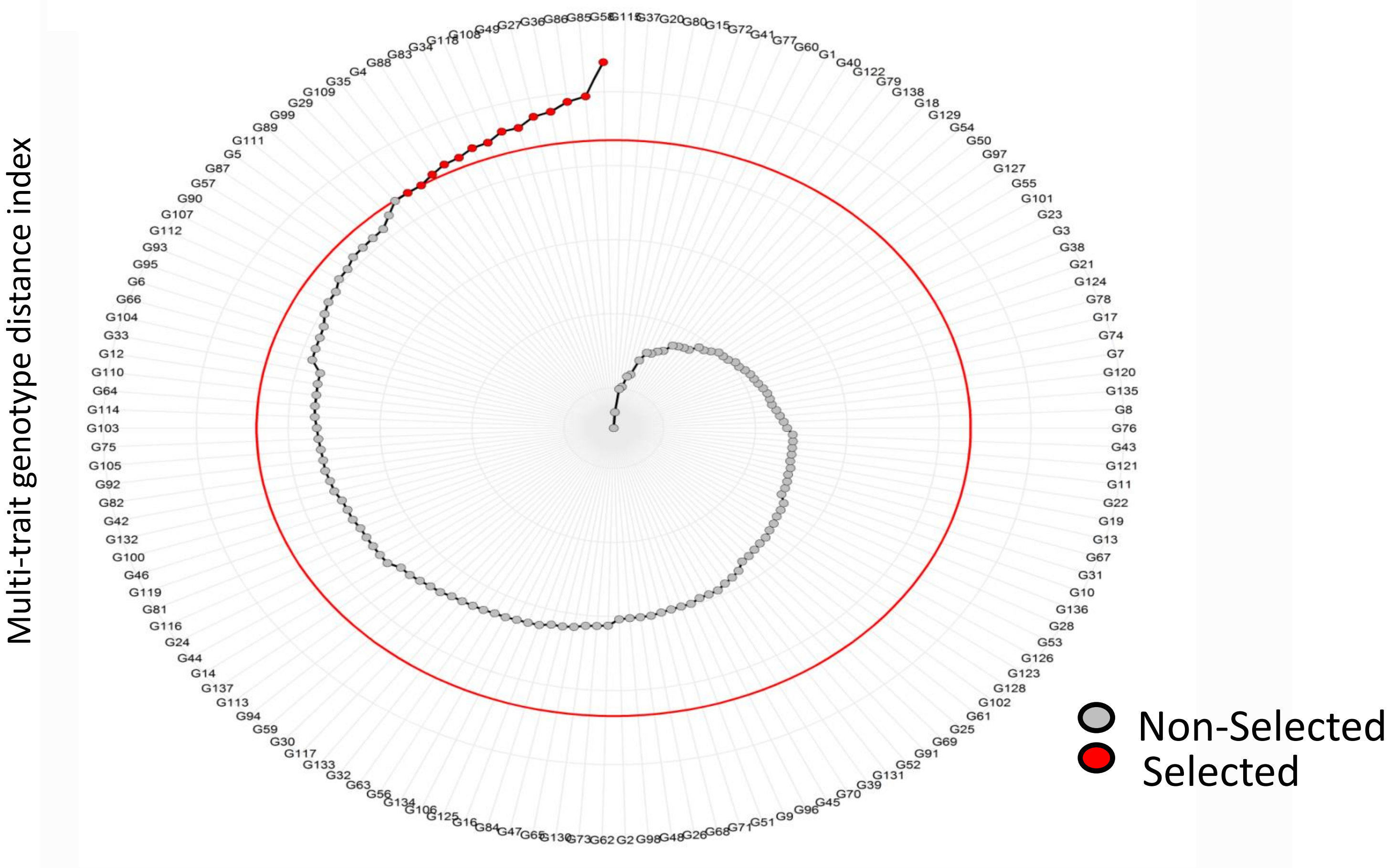
Multi-trait genotype distance index
Non-Selected
Selected

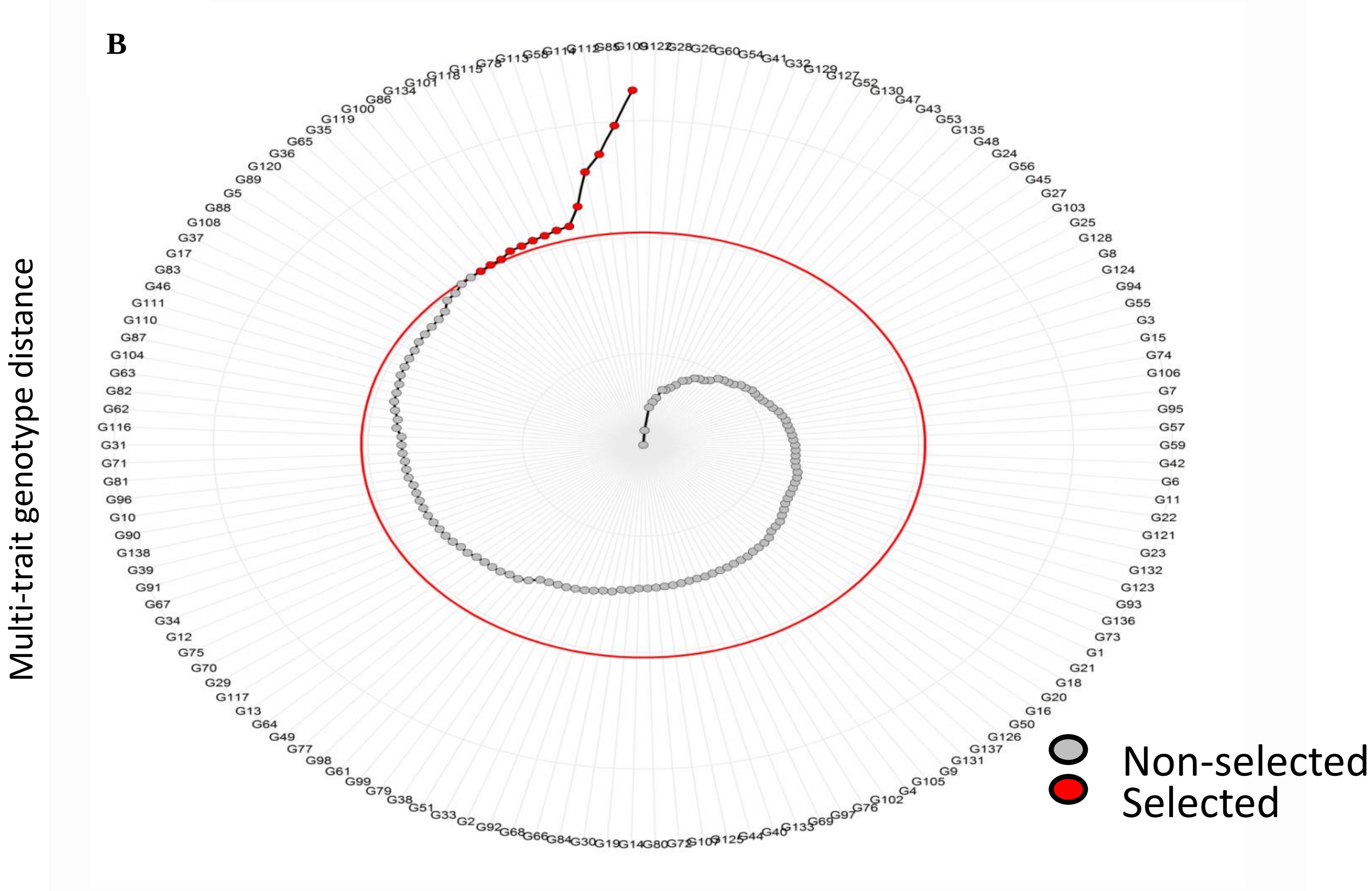
B
Multi-trait genotype distance
Non-selected
Selected

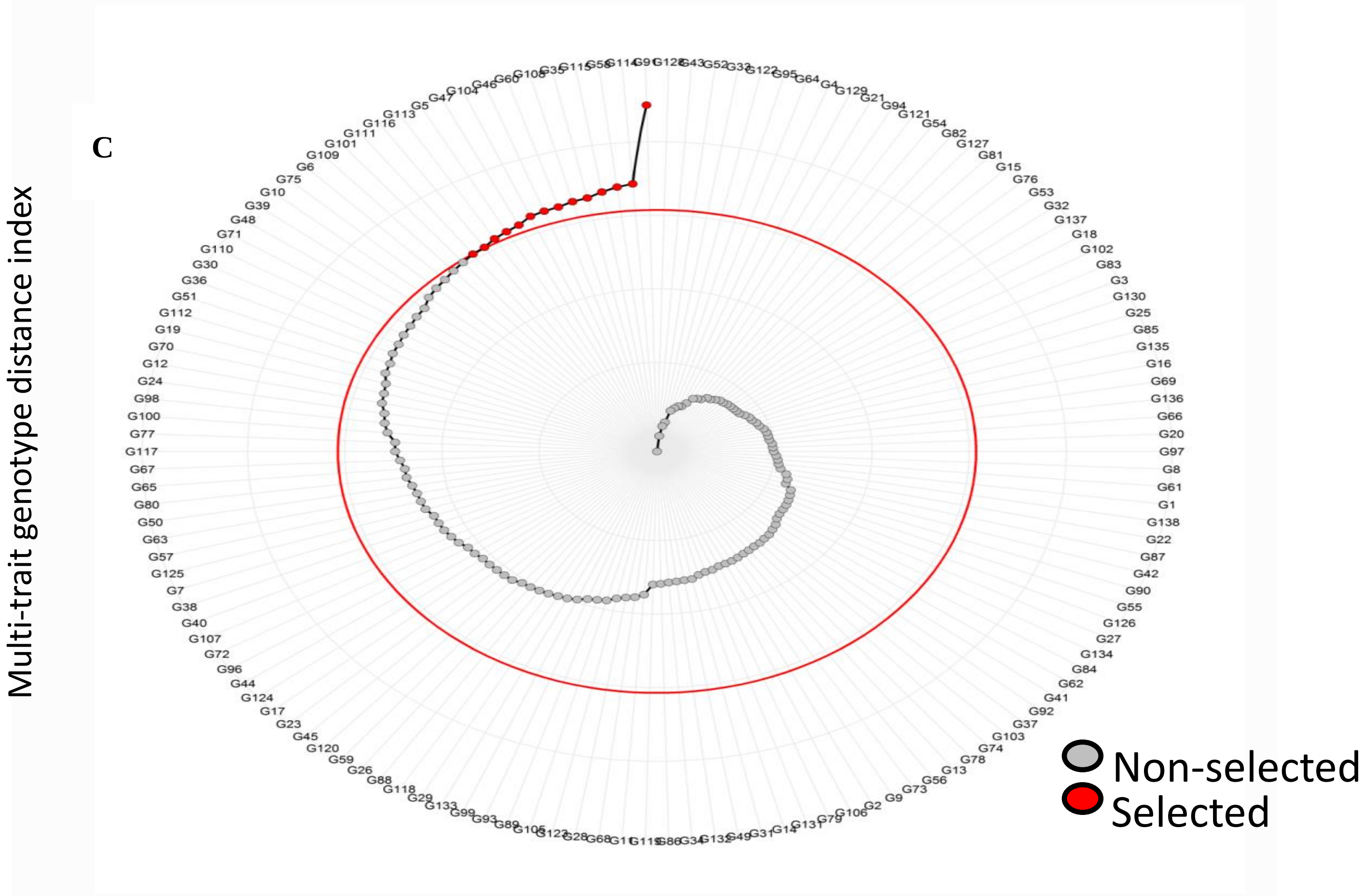

Supplementary Figure S3 Ranking of genotypes based on MGIDI index and *Strengths* and *weakness* view of the stable genotypes identified in Control-0 mM (A) S1-150 mM (B) S2-250 mM . In this figure, the red circle indicates the cut point according to the selection pressure (SI = 10%). The MGIDI index identified 14 genotypes as more desirable accessions than others for each treatment.
