# Supplementary material for: A GBS-based genome-wide association study reveals the genetic basis of salinity tolerance at the seedling stage in bread wheat (Triticum aestivum L.)
Source: Front Genet. 2022 Sep 27;13:997901. doi: 10.3389/fgene.2022.997901 (PMC9551609; doi:10.3389/fgene.2022.997901)
Supplement: Supplementary file 8 [file Table2.DOCX]

Supplementary Material


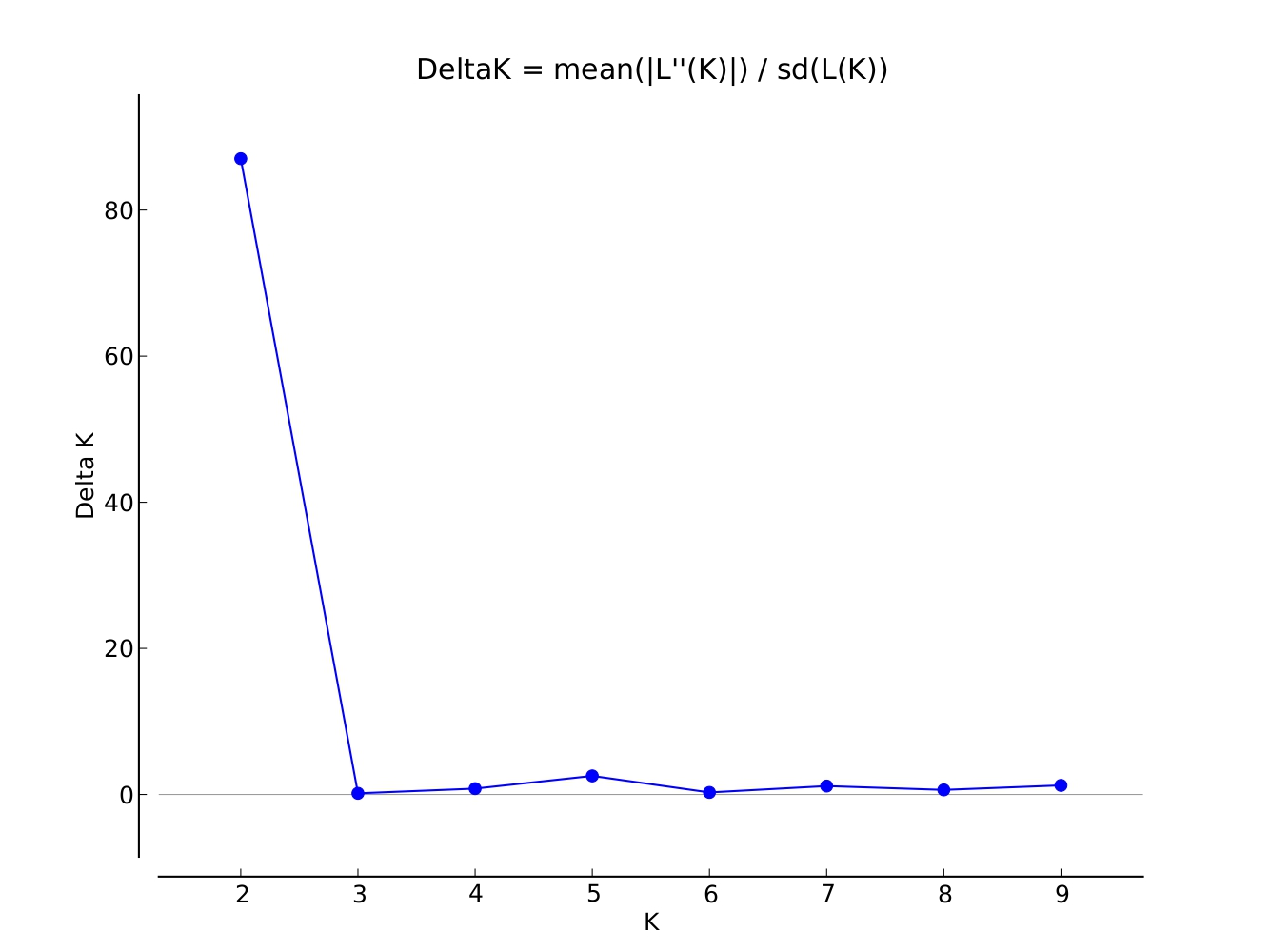


**Supplementary Figure 1** ΔK versus sub-groups K plot with arrow pointing to the number of sub-populations in the germplasm


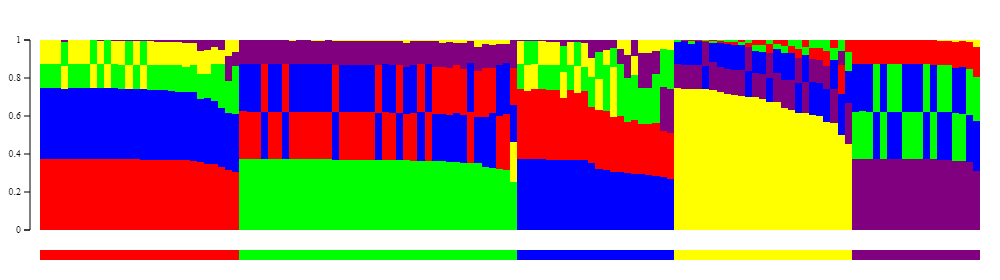


**Q5**

**Q4**

**Q3**

**Q2**

**Q1**

**Supplementary Figure 2** structure analysis dividing the population into five sub-group


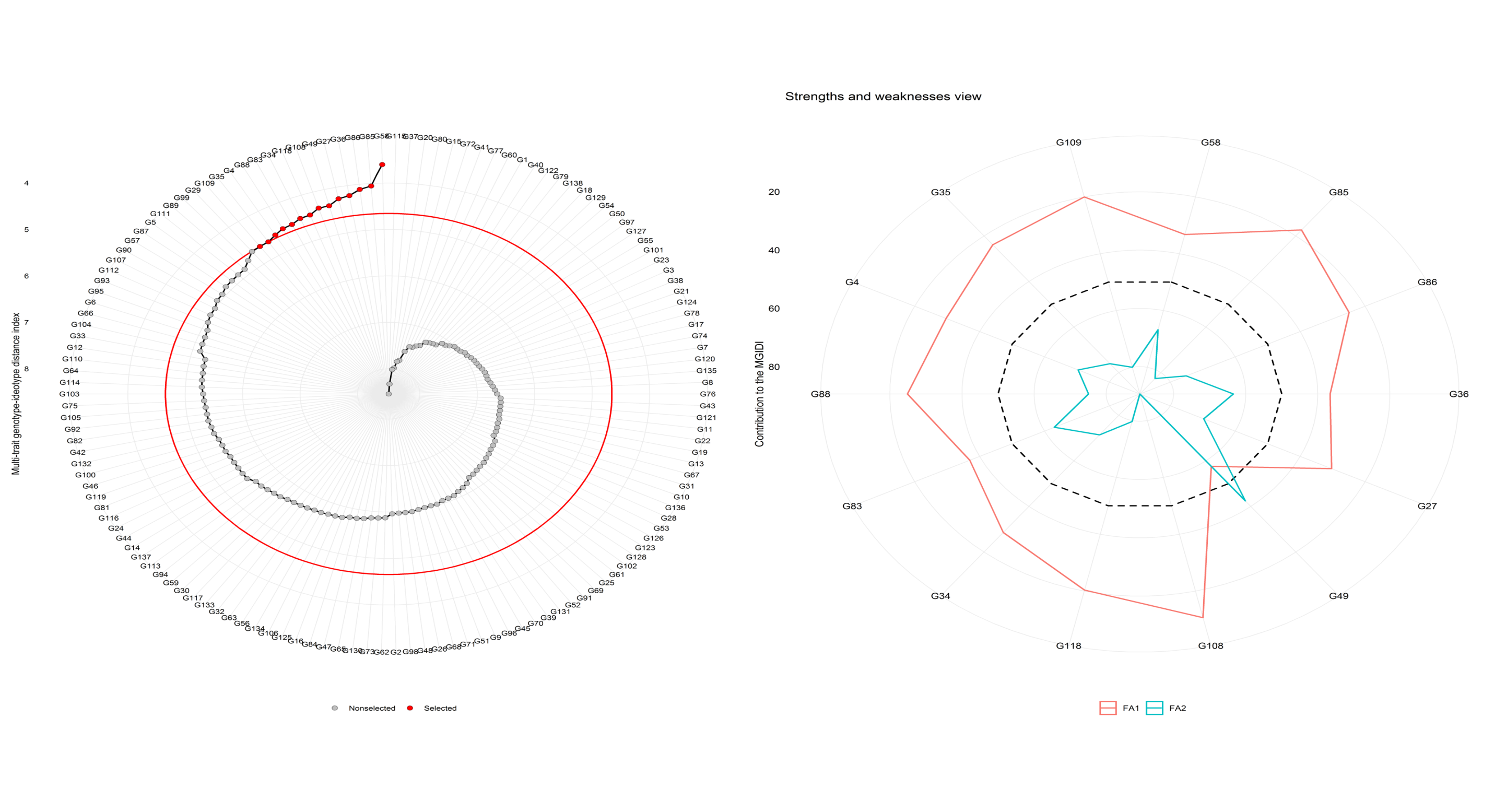


Selected

Non-Selected

Multi-trait genotype distance index

**A**


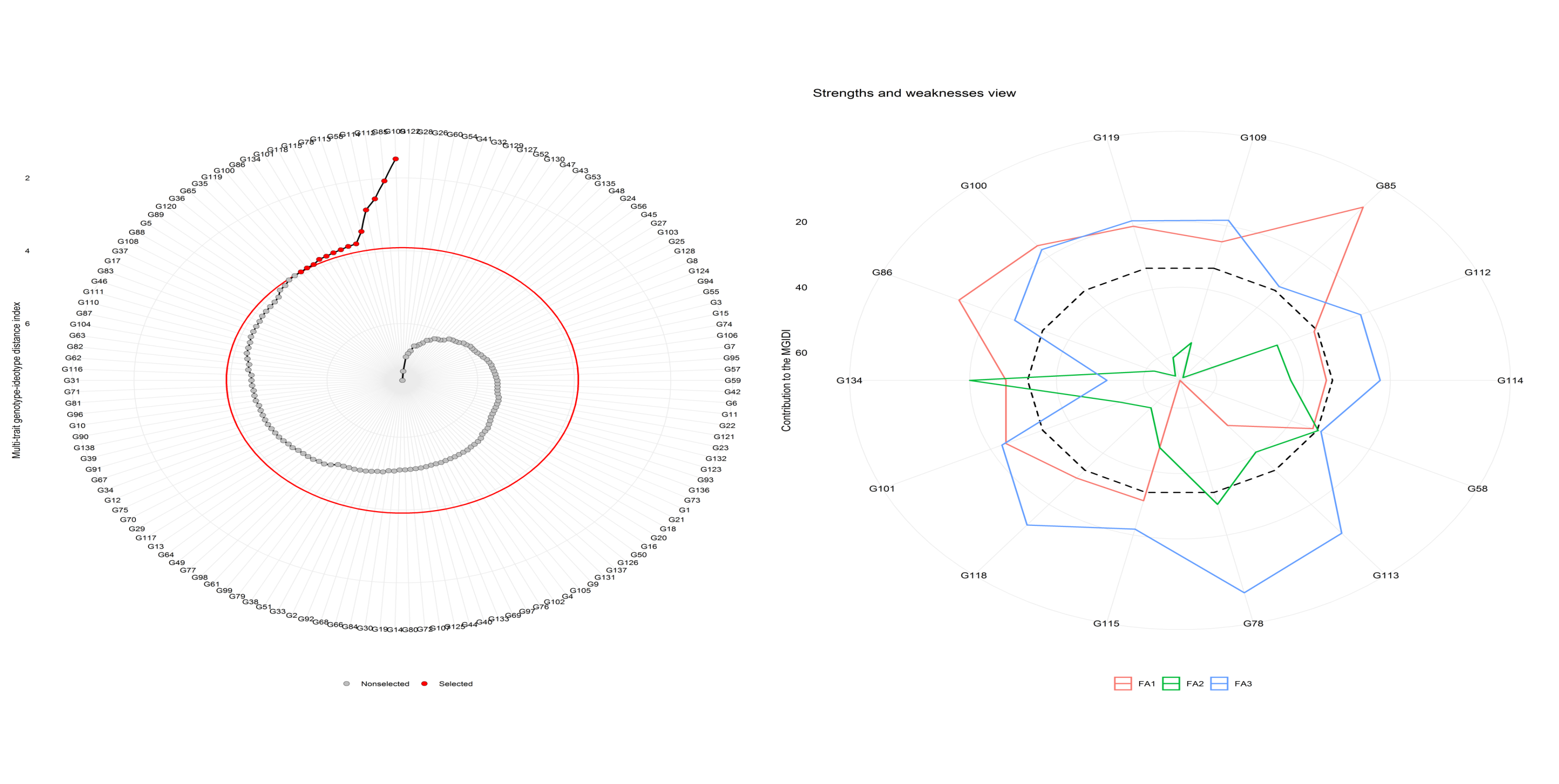


Multi-trait genotype distance index

Non-selected

Selected

**B**


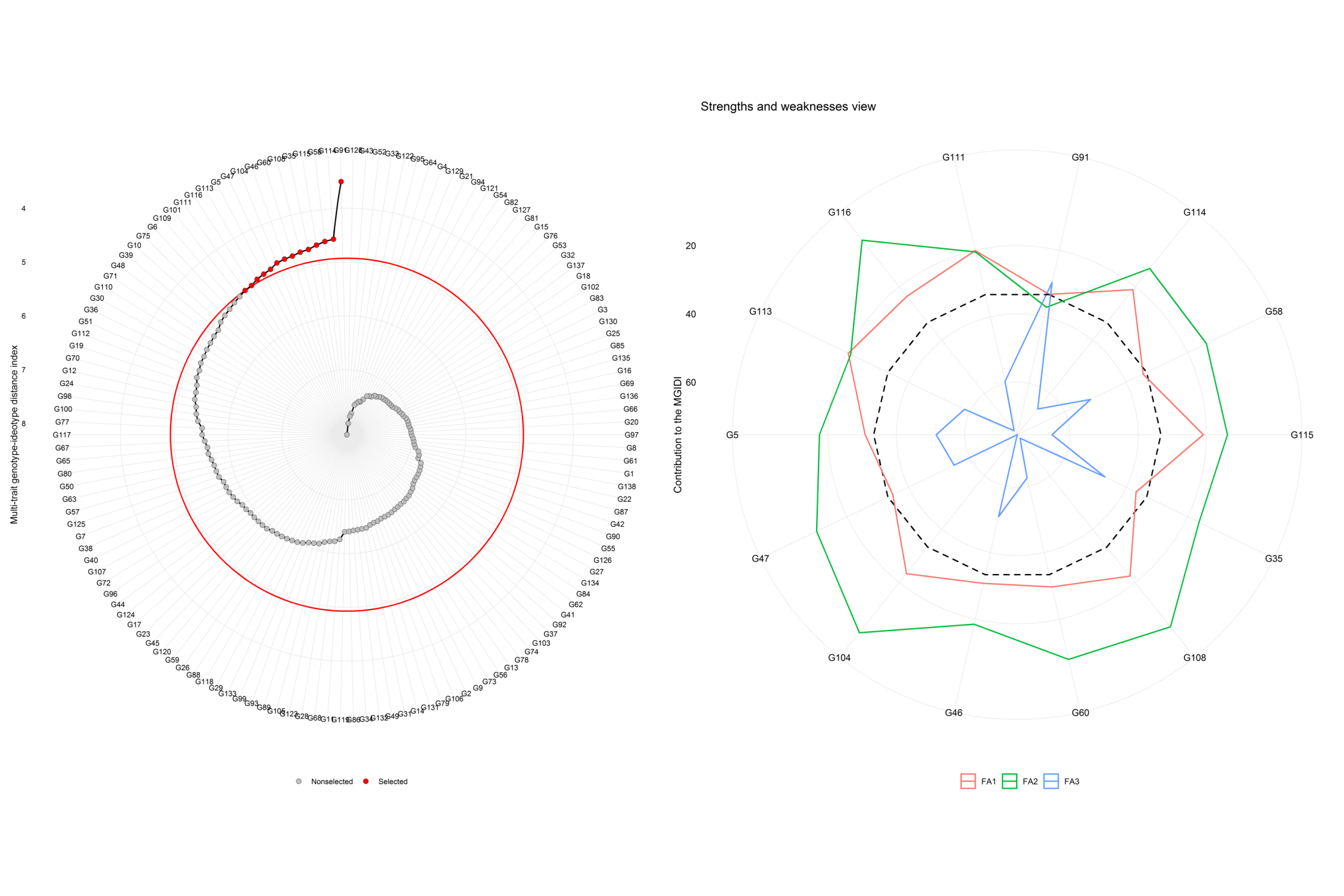


Selected

Multi-trait genotype distance index

Non-selected

**C**

**Supplementary Figure 3** Ranking of genotypes based on MGIDI index and *Strengths* and *weakness* view of the stable genotypes identified in Control-0 mM (A) S1-150 mM (B) S2-250 mM . In this figure, the red circle indicates the cut point according to the selection pressure (SI = 10%). The MGIDI index identified 14 genotypes as more desirable accessions than others for each treatment.

**Supplementary Table 1 Pedigree of germplasm**

| **Sr. No.** | **Genotype** | **GID Number** | **Pedigree** | **Q Group** |
| --- | --- | --- | --- | --- |
| 1 | SEEDLTPP2 | 7640766 | CETA/AE.SQUARROSA (665)//KACHU/3/BAJ #1 | Q1 |
| 2 | SEEDLTPP59 | 7640823 | KE90-282/MILAN//KACHU/3/BAJ #1 |  |
| 3 | SEEDLTPP69 | 7640833 | KE90-282/MILAN//KACHU/3/BAJ #1 | Q3 |
| 4 | SEEDLTPP90 | 7640854 | KE90-282/MILAN//KACHU/3/BAJ #1 | Q1 |
| 5 | SEEDLTPP138 | 7640902 | IG 41485/KACHU//BAJ #1 | Q3 |
| 6 | SEEDLTPP176 | 7640940 | IG 42158/KACHU//BAJ #1 | Q1 |
| 7 | SEEDLTPP2437 | 7640963 | SCOOP_1/AE.SQUARROSA (634)//KACHU/3/BAJ #1 | Q1 |
| 8 | SEEDLTPP2446 | 7641255 | TK SN1081/AE.SQUARROSA (690)//KACHU/3/BAJ #1 | Q1 |
| 9 | SEEDLTPP2450 | 7641281 | GAN/AE.SQUARROSA (897)//KACHU/3/BAJ #1 | Q3 |
| 10 | SEEDLTPP2456 | 7641318 | CROC_1/AE.SQUARROSA (516)//KACHU/3/BAJ #1 | Q1 |
| 11 | SEEDLTPP2457 | 7641324 | CROC_1/AE.SQUARROSA (516)//KACHU/3/BAJ #1 | Q3 |
| 12 | SEEDLTPP2460 | 7641333 | CROC_1/AE.SQUARROSA (516)//KACHU/3/BAJ #1 | Q3 |
| 13 | SEEDLTPP2463 | 7641340 | CROC_1/AE.SQUARROSA (517)//KACHU/3/BAJ #1 | Q1 |
| 14 | SEEDLTPP2464 | 7641343 | CROC_1/AE.SQUARROSA (517)//KACHU/3/BAJ #1 |  |
| 15 | SEEDLTPP2486 | 7641405 | DOY1/AE.SQUARROSA (1024)//KACHU/3/BAJ #1 | Q1 |
| 16 | SEEDLTPP2487 | 7641406 | DOY1/AE.SQUARROSA (1024)//KACHU/3/BAJ #1 | Q1 |
| 17 | SEEDLTPP2489 | 7641408 | DOY1/AE.SQUARROSA (1024)//KACHU/3/BAJ #1 | Q3 |
| 18 | SEEDLTPP2491 | 7641418 | CETA/AE.SQUARROSA (1025)//KACHU/3/BAJ #1 | Q1 |
| 19 | SEEDLTPP2508 | 7641461 | ARLIN_1/AE.SQUARROSA (1017)//KACHU/3/BAJ #1 | Q1 |
| 20 | SEEDLTPP2520 | 7641563 | D67.2/PARANA 66.270//AE.SQUARROSA (185)/3/KACHU/4/BAJ #1 | Q3 |
| 21 | SEEDLTPP195 | 7641608 | T.DICOCCON PI347230/AE.SQUARROSA (879)//KACHU/3/BAJ #1 | Q1 |
| 22 | SEEDLTPP2524 | 7641628 | GAN/AE.SQUARROSA (680)//KACHU/3/BAJ #1 | Q1 |
| 23 | SEEDLTPP2534 | 7641701 | CROC_1/AE.SQUARROSA (298)//KACHU/3/BAJ #1 | Q1 |
| 24 | SEEDLTPP2539 | 7641715 | CROC_1/AE.SQUARROSA (298)//KACHU/3/BAJ #1 | Q1 |
| 25 | SEEDLTPP216 | 7641741 | DVERD_2/T.URARTU (545)//KACHU/3/BAJ #1 | Q1 |
| 26 | SEEDLTPP2549 | 7641789 | IG 42153/KACHU//BAJ #1 | Q1 |
| 27 | SEEDLTPP2550 | 7641791 | IG 42153/KACHU//BAJ #1 | Q1 |
| 28 | SEEDLTPP242 | 7641797 | H-1442/KACHU//BAJ #1 | Q3 |
| 29 | SEEDLTPP257 | 7641812 | H-1442/KACHU//BAJ #1 | Q1 |
| 30 | SEEDLTPP258 | 7641813 | H-1442/KACHU//BAJ #1 | Q1 |
| 31 | SEEDLTPP259 | 7641814 | H-1442/KACHU//BAJ #1 | Q1 |
| 32 | SEEDLTPP270 | 7641825 | H-1442/KACHU//BAJ #1 | Q1 |
| 33 | SEEDLTPP277 | 7641832 | H-1442/KACHU//BAJ #1 | Q1 |
| 34 | SEEDLTPP279 | 7641834 | H-1442/KACHU//BAJ #1 | Q1 |
| 35 | SEEDLTPP290 | 7641845 | W47/KACHU//BAJ #1 | Q1 |
| 36 | SEEDLTPP303 | 7641858 | W47/KACHU//BAJ #1 | Q3 |
| 37 | SEEDLTPP304 | 7641859 | W47/KACHU//BAJ #1 | Q3 |
| 38 | SEEDLTPP305 | 7641860 | W47/KACHU//BAJ #1 | Q3 |
| 39 | SEEDLTPP362 | 7641917 | CHEN/AE.SQ//2*OPATA/3/BAJ #1/4/SUP152 | Q3 |
| 40 | SEEDLTPP363 | 7641918 | CHEN/AE.SQ//2*OPATA/3/BAJ #1/4/SUP152 | Q3 |
| 41 | SEEDLTPP366 | 7641921 | CHEN/AE.SQ//2*OPATA/3/BAJ #1/4/SUP152 | Q5 |
| 42 | SEEDLTPP371 | 7641926 | CHEN/AE.SQ//2*OPATA/3/BAJ #1/4/SUP152 | Q5 |
| 43 | SEEDLTPP375 | 7641930 | CHEN/AE.SQ//2*OPATA/3/BAJ #1/4/SUP152 | Q5 |
| 44 | SEEDLTPP386 | 7641941 | CHEN/AE.SQ//2*OPATA/3/BAJ #1/4/SUP152 | Q5 |
| 45 | SEEDLTPP404 | 7641959 | CHEN/AE.SQ//2*OPATA/3/BAJ #1/4/SUP152 | Q5 |
| 46 | SEEDLTPP407 | 7641962 | CHEN/AE.SQ//2*OPATA/3/BAJ #1/4/SUP152 | Q5 |
| 47 | SEEDLTPP411 | 7641966 | CHEN/AE.SQ//2*OPATA/3/BAJ #1/4/SUP152 | Q5 |
| 48 | SEEDLTPP412 | 7641967 | CHEN/AE.SQ//2*OPATA/3/BAJ #1/4/SUP152 | Q5 |
| 49 | SEEDLTPP415 | 7641970 | CHEN/AE.SQ//2*OPATA/3/BAJ #1/4/SUP152 |  |
| 50 | SEEDLTPP416 | 7641971 | CHEN/AE.SQ//2*OPATA/3/BAJ #1/4/SUP152 | Q5 |
| 51 | SEEDLTPP418 | 7641973 | CHEN/AE.SQ//2*OPATA/3/BAJ #1/4/SUP152 | Q5 |
| 52 | SEEDLTPP420 | 7641975 | CHEN/AE.SQ//2*OPATA/3/BAJ #1/4/SUP152 | Q5 |
| 53 | SEEDLTPP2551 | 7641999 | IG 42161/BAJ #1//SUP152 | Q5 |
| 54 | SEEDLTPP2552 | 7642000 | IG 42161/BAJ #1//SUP152 | Q5 |
| 55 | SEEDLTPP2553 | 7642005 | IG 42161/BAJ #1//SUP152 | Q5 |
| 56 | SEEDLTPP2562 | 7642080 | D67.2/PARANA 66.270//AE.SQUARROSA (320)/3/BAJ #1/4/SUP152 | Q3 |
| 57 | SEEDLTPP2564 | 7642087 | D67.2/PARANA 66.270//AE.SQUARROSA (643)/3/BAJ #1/4/SUP152 | Q5 |
| 58 | SEEDLTPP2567 | 7642123 | CETA/AE.SQUARROSA (409)//BAJ #1/3/SUP152 | Q5 |
| 59 | SEEDLTPP2569 | 7642161 | DOY1/AE.SQUARROSA (264)//BAJ #1/3/SUP152 | Q5 |
| 60 | SEEDLTPP2571 | 7642168 | DOY1/AE.SQUARROSA (264)//BAJ #1/3/SUP152 | Q5 |
| 61 | SEEDLTPP2572 | 7642171 | DOY1/AE.SQUARROSA (264)//BAJ #1/3/SUP152 | Q1 |
| 62 | SEEDLTPP2592 | 7642284 | BCN/4/RABI//GS/CRA/3/AE.SQUARROSA (895)/5/SUP152/6/VILLA JUAREZ F2009 | Q3 |
| 63 | SEEDLTPP2594 | 7642291 | BCN/4/RABI//GS/CRA/3/AE.SQUARROSA (895)/5/SUP152/6/VILLA JUAREZ F2009 | Q3 |
| 64 | SEEDLTPP2595 | 7642292 | BCN/4/RABI//GS/CRA/3/AE.SQUARROSA (895)/5/SUP152/6/VILLA JUAREZ F2009 | Q3 |
| 65 | SEEDLTPP2600 | 7642310 | BCN/4/RABI//GS/CRA/3/AE.SQUARROSA (895)/5/SUP152/6/VILLA JUAREZ F2009 |  |
| 66 | SEEDLTPP2602 | 7642323 | BCN/4/RABI//GS/CRA/3/AE.SQUARROSA (895)/5/SUP152/6/VILLA JUAREZ F2009 | Q4 |
| 67 | SEEDLTPP2603 | 7642324 | BCN/4/RABI//GS/CRA/3/AE.SQUARROSA (895)/5/SUP152/6/VILLA JUAREZ F2009 | Q4 |
| 68 | SEEDLTPP2608 | 7642370 | SORA/AE.SQUARROSA (442)//SUP152/3/VILLA JUAREZ F2009 | Q4 |
| 69 | SEEDLTPP2610 | 7642372 | SORA/AE.SQUARROSA (442)//SUP152/3/VILLA JUAREZ F2009 | Q4 |
| 70 | SEEDLTPP2611 | 7642377 | SORA/AE.SQUARROSA (442)//SUP152/3/VILLA JUAREZ F2009 | Q4 |
| 71 | SEEDLTPP2612 | 7642378 | SORA/AE.SQUARROSA (442)//SUP152/3/VILLA JUAREZ F2009 | Q4 |
| 72 | SEEDLTPP2613 | 7642379 | SORA/AE.SQUARROSA (442)//SUP152/3/VILLA JUAREZ F2009 | Q4 |
| 73 | SEEDLTPP2617 | 7642410 | IG 122628/SUP152//VILLA JUAREZ F2009 | Q4 |
| 74 | SEEDLTPP2623 | 7642436 | BCN//SORA/AE.SQUARROSA (323)/3/VILLA JUAREZ F2009/4/WBLL1/KUKUNA//TACUPETO F2001/3/BAJ #1 | Q4 |
| 75 | SEEDLTPP2624 | 7642443 | BCN//SORA/AE.SQUARROSA (323)/3/VILLA JUAREZ F2009/4/WBLL1/KUKUNA//TACUPETO F2001/3/BAJ #1 | Q4 |
| 76 | SEEDLTPP440 | 7642447 | BCN//CETA/AE.SEARSII (34D)/3/VILLA JUAREZ F2009/4/WBLL1/KUKUNA//TACUPETO F2001/3/BAJ #1 | Q4 |
| 77 | SEEDLTPP449 | 7642456 | BCN//CETA/AE.SEARSII (34D)/3/VILLA JUAREZ F2009/4/WBLL1/KUKUNA//TACUPETO F2001/3/BAJ #1 | Q4 |
| 78 | SEEDLTPP457 | 7642464 | BCN//CETA/AE.SEARSII (34D)/3/VILLA JUAREZ F2009/4/WBLL1/KUKUNA//TACUPETO F2001/3/BAJ #1 |  |
| 79 | SEEDLTPP463 | 7642470 | BCN//CETA/AE.SEARSII (34D)/3/VILLA JUAREZ F2009/4/WBLL1/KUKUNA//TACUPETO F2001/3/BAJ #1 | Q4 |
| 80 | SEEDLTPP469 | 7642476 | BCN//CETA/AE.SEARSII (34D)/3/VILLA JUAREZ F2009/4/WBLL1/KUKUNA//TACUPETO F2001/3/BAJ #1 | Q4 |
| 81 | SEEDLTPP470 | 7642477 | BCN//CETA/AE.SEARSII (34D)/3/VILLA JUAREZ F2009/4/WBLL1/KUKUNA//TACUPETO F2001/3/BAJ #1 | Q4 |
| 82 | SEEDLTPP2628 | 7642498 | CHEN/AE.SQ//WEAVER/3/VILLA JUAREZ F2009/4/WBLL1/KUKUNA//TACUPETO F2001/3/BAJ #1 | Q4 |
| 83 | SEEDLTPP2637 | 7642579 | IG 42157/VILLA JUAREZ F2009/4/WBLL1/KUKUNA//TACUPETO F2001/3/BAJ #1 | Q4 |
| 84 | SEEDLTPP2648 | 7642624 | IG 122793/VILLA JUAREZ F2009/4/WBLL1/KUKUNA//TACUPETO F2001/3/BAJ #1 | Q4 |
| 85 | SEEDLTPP2649 | 7642627 | IG 122793/VILLA JUAREZ F2009/4/WBLL1/KUKUNA//TACUPETO F2001/3/BAJ #1 | Q4 |
| 86 | SEEDLTPP2652 | 7642641 | IG 126482/VILLA JUAREZ F2009/4/WBLL1/KUKUNA//TACUPETO F2001/3/BAJ #1 | Q4 |
| 87 | SEEDLTPP493 | 7642653 | BCN//CETA/AE.SEARSII (34D)/4/WBLL1/KUKUNA//TACUPETO F2001/3/BAJ #1/5/SERI.1B//KAUZ/HEVO/3/AMAD*2/4/KIRITATI | Q4 |
| 88 | SEEDLTPP497 | 7642657 | BCN//CETA/AE.SEARSII (34D)/4/WBLL1/KUKUNA//TACUPETO F2001/3/BAJ #1/5/SERI.1B//KAUZ/HEVO/3/AMAD*2/4/KIRITATI | Q4 |
| 89 | SEEDLTPP498 | 7642658 | BCN//CETA/AE.SEARSII (34D)/4/WBLL1/KUKUNA//TACUPETO F2001/3/BAJ #1/5/SERI.1B//KAUZ/HEVO/3/AMAD*2/4/KIRITATI | Q4 |
| 90 | SEEDLTPP499 | 7642659 | BCN//CETA/AE.SEARSII (34D)/4/WBLL1/KUKUNA//TACUPETO F2001/3/BAJ #1/5/SERI.1B//KAUZ/HEVO/3/AMAD*2/4/KIRITATI | Q4 |
| 91 | SEEDLTPP502 | 7642662 | BCN//CETA/AE.SEARSII (34D)/4/WBLL1/KUKUNA//TACUPETO F2001/3/BAJ #1/5/SERI.1B//KAUZ/HEVO/3/AMAD*2/4/KIRITATI | Q4 |
| 92 | SEEDLTPP536 | 7642696 | BCN//SORA/AE.SQUARROSA (323)/4/WBLL1/KUKUNA//TACUPETO F2001/3/BAJ #1/5/SERI.1B//KAUZ/HEVO/3/AMAD*2/4/KIRITATI | Q2 |
| 93 | SEEDLTPP542 | 7642702 | BCN//SORA/AE.SQUARROSA (323)/4/WBLL1/KUKUNA//TACUPETO F2001/3/BAJ #1/5/SERI.1B//KAUZ/HEVO/3/AMAD*2/4/KIRITATI | Q2 |
| 94 | SEEDLTPP544 | 7642704 | BCN//SORA/AE.SQUARROSA (323)/4/WBLL1/KUKUNA//TACUPETO F2001/3/BAJ #1/5/SERI.1B//KAUZ/HEVO/3/AMAD*2/4/KIRITATI | Q2 |
| 95 | SEEDLTPP545 | 7642705 | BCN//SORA/AE.SQUARROSA (323)/4/WBLL1/KUKUNA//TACUPETO F2001/3/BAJ #1/5/SERI.1B//KAUZ/HEVO/3/AMAD*2/4/KIRITATI | Q2 |
| 96 | SEEDLTPP549 | 7642709 | BCN//SORA/AE.SQUARROSA (323)/4/WBLL1/KUKUNA//TACUPETO F2001/3/BAJ #1/5/SERI.1B//KAUZ/HEVO/3/AMAD*2/4/KIRITATI | Q2 |
| 97 | SEEDLTPP550 | 7642710 | BCN//SORA/AE.SQUARROSA (323)/4/WBLL1/KUKUNA//TACUPETO F2001/3/BAJ #1/5/SERI.1B//KAUZ/HEVO/3/AMAD*2/4/KIRITATI | Q2 |
| 98 | SEEDLTPP553 | 7642713 | BCN//SORA/AE.SQUARROSA (323)/4/WBLL1/KUKUNA//TACUPETO F2001/3/BAJ #1/5/SERI.1B//KAUZ/HEVO/3/AMAD*2/4/KIRITATI | Q2 |
| 99 | SEEDLTPP561 | 7642721 | BCN//SORA/AE.SQUARROSA (323)/4/WBLL1/KUKUNA//TACUPETO F2001/3/BAJ #1/5/SERI.1B//KAUZ/HEVO/3/AMAD*2/4/KIRITATI | Q2 |
| 100 | SEEDLTPP563 | 7642723 | BCN//SORA/AE.SQUARROSA (323)/4/WBLL1/KUKUNA//TACUPETO F2001/3/BAJ #1/5/SERI.1B//KAUZ/HEVO/3/AMAD*2/4/KIRITATI | Q2 |
| 101 | SEEDLTPP564 | 7642724 | BCN//SORA/AE.SQUARROSA (323)/4/WBLL1/KUKUNA//TACUPETO F2001/3/BAJ #1/5/SERI.1B//KAUZ/HEVO/3/AMAD*2/4/KIRITATI | Q2 |
| 102 | SEEDLTPP573 | 7642733 | CHEN/AE.SQ//2*OPATA/4/WBLL1/KUKUNA//TACUPETO F2001/3/BAJ #1/5/SERI.1B//KAUZ/HEVO/3/AMAD*2/4/KIRITATI | Q2 |
| 103 | SEEDLTPP588 | 7642748 | CHEN/AE.SQ//2*OPATA/4/WBLL1/KUKUNA//TACUPETO F2001/3/BAJ #1/5/SERI.1B//KAUZ/HEVO/3/AMAD*2/4/KIRITATI | Q2 |
| 104 | SEEDLTPP594 | 7642754 | CHEN/AE.SQ//2*OPATA/4/WBLL1/KUKUNA//TACUPETO F2001/3/BAJ #1/5/SERI.1B//KAUZ/HEVO/3/AMAD*2/4/KIRITATI | Q2 |
| 105 | SEEDLTPP611 | 7642771 | ARVAND 1/4/WBLL1/KUKUNA//TACUPETO F2001/3/BAJ #1/5/SERI.1B//KAUZ/HEVO/3/AMAD*2/4/KIRITATI | Q2 |
| 106 | SEEDLTPP617 | 7642777 | ARVAND 1/4/WBLL1/KUKUNA//TACUPETO F2001/3/BAJ #1/5/SERI.1B//KAUZ/HEVO/3/AMAD*2/4/KIRITATI | Q2 |
| 107 | SEEDLTPP621 | 7642781 | ARVAND 1/4/WBLL1/KUKUNA//TACUPETO F2001/3/BAJ #1/5/SERI.1B//KAUZ/HEVO/3/AMAD*2/4/KIRITATI | Q2 |
| 108 | SEEDLTPP626 | 7642786 | ARVAND 1/4/WBLL1/KUKUNA//TACUPETO F2001/3/BAJ #1/5/SERI.1B//KAUZ/HEVO/3/AMAD*2/4/KIRITATI | Q2 |
| 109 | SEEDLTPP2655 | 7642809 | ARLIN_1/AE.SQUARROSA (536)/4/WBLL1/KUKUNA//TACUPETO F2001/3/BAJ #1/5/SERI.1B//KAUZ/HEVO/3/AMAD*2/4/KIRITATI | Q2 |
| 110 | SEEDLTPP2656 | 7642821 | ARLIN_1/AE.SQUARROSA (536)/4/WBLL1/KUKUNA//TACUPETO F2001/3/BAJ #1/5/SERI.1B//KAUZ/HEVO/3/AMAD*2/4/KIRITATI | Q2 |
| 111 | SEEDLTPP2659 | 7642850 | CETA/AE.SQUARROSA (1036)/4/WBLL1/KUKUNA//TACUPETO F2001/3/BAJ #1/5/SERI.1B//KAUZ/HEVO/3/AMAD*2/4/KIRITATI | Q2 |
| 112 | SEEDLTPP2660 | 7642860 | CETA/AE.SQUARROSA (1036)/4/WBLL1/KUKUNA//TACUPETO F2001/3/BAJ #1/5/SERI.1B//KAUZ/HEVO/3/AMAD*2/4/KIRITATI | Q2 |
| 113 | SEEDLTPP2664 | 7642888 | CPI8/GEDIZ/3/GOO//ALB/CRA/4/AE.SQUARROSA (1038)/5/WBLL1/KUKUNA//TACUPETO F2001/3/BAJ #1/6/SERI.1B//KAUZ/HEVO/3/AMAD*2/4/KIRITATI | Q2 |
| 114 | SEEDLTPP2665 | 7642891 | CPI8/GEDIZ/3/GOO//ALB/CRA/4/AE.SQUARROSA (1038)/5/WBLL1/KUKUNA//TACUPETO F2001/3/BAJ #1/6/SERI.1B//KAUZ/HEVO/3/AMAD*2/4/KIRITATI |  |
| 115 | SEEDLTPP2666 | 7642901 | D67.2/PARANA 66.270//AE.SQUARROSA (828)/4/WBLL1/KUKUNA//TACUPETO F2001/3/BAJ #1/5/SERI.1B//KAUZ/HEVO/3/AMAD*2/4/KIRITATI | Q2 |
| 116 | SEEDLTPP2667 | 7642903 | D67.2/PARANA 66.270//AE.SQUARROSA (828)/4/WBLL1/KUKUNA//TACUPETO F2001/3/BAJ #1/5/SERI.1B//KAUZ/HEVO/3/AMAD*2/4/KIRITATI | Q2 |
| 117 | SEEDLTPP2670 | 7642906 | D67.2/PARANA 66.270//AE.SQUARROSA (828)/4/WBLL1/KUKUNA//TACUPETO F2001/3/BAJ #1/5/SERI.1B//KAUZ/HEVO/3/AMAD*2/4/KIRITATI | Q2 |
| 118 | SEEDLTPP2671 | 7642907 | D67.2/PARANA 66.270//AE.SQUARROSA (828)/4/WBLL1/KUKUNA//TACUPETO F2001/3/BAJ #1/5/SERI.1B//KAUZ/HEVO/3/AMAD*2/4/KIRITATI | Q2 |
| 119 | SEEDLTPP2674 | 7642911 | D67.2/PARANA 66.270//AE.SQUARROSA (828)/4/WBLL1/KUKUNA//TACUPETO F2001/3/BAJ #1/5/SERI.1B//KAUZ/HEVO/3/AMAD*2/4/KIRITATI | Q2 |
| 120 | SEEDLTPP2675 | 7642917 | D67.2/PARANA 66.270//AE.SQUARROSA (828)/4/WBLL1/KUKUNA//TACUPETO F2001/3/BAJ #1/5/SERI.1B//KAUZ/HEVO/3/AMAD*2/4/KIRITATI | Q2 |
| 121 | SEEDLTPP2680 | 7642934 | GAN/AE.SQUARROSA (741)/4/WBLL1/KUKUNA//TACUPETO F2001/3/BAJ #1/5/SERI.1B//KAUZ/HEVO/3/AMAD*2/4/KIRITATI | Q2 |
| 122 | SEEDLTPP2684 | 7642941 | GAN/AE.SQUARROSA (741)/4/WBLL1/KUKUNA//TACUPETO F2001/3/BAJ #1/5/SERI.1B//KAUZ/HEVO/3/AMAD*2/4/KIRITATI | Q2 |
| 123 | SEEDLTPP2689 | 7642976 | 68.111/RGB-U//WARD RESEL/3/STIL/4/AE.SQUARROSA (188)/5/WBLL1/KUKUNA//TACUPETO F2001/3/BAJ #1/6/SERI.1B//KAUZ/HEVO/3/AMAD*2/4/KIRITATI | Q2 |
| 124 | SEEDLTPP2690 | 7642977 | 68.111/RGB-U//WARD RESEL/3/STIL/4/AE.SQUARROSA (188)/5/WBLL1/KUKUNA//TACUPETO F2001/3/BAJ #1/6/SERI.1B//KAUZ/HEVO/3/AMAD*2/4/KIRITATI | Q2 |
| 125 | SEEDLTPP2715 | 7643065 | IWA8611400/4/WBLL1/KUKUNA//TACUPETO F2001/3/BAJ #1/5/SERI.1B//KAUZ/HEVO/3/AMAD*2/4/KIRITATI | Q2 |
| 126 | SEEDLTPP2717 | 7643067 | IWA8611400/4/WBLL1/KUKUNA//TACUPETO F2001/3/BAJ #1/5/SERI.1B//KAUZ/HEVO/3/AMAD*2/4/KIRITATI | Q2 |
| 127 | SEEDLTPP2718 | 7643071 | CHEN/AE.SQ//2*OPATA/5/SERI.1B//KAUZ/HEVO/3/AMAD*2/4/KIRITATI/6/FRET2*2/4/SNI/TRAP#1/3/KAUZ*2/TRAP//KAUZ/5/KACHU | Q2 |
| 128 | SEEDLTPP2719 | 7643072 | CHEN/AE.SQ//2*OPATA/5/SERI.1B//KAUZ/HEVO/3/AMAD*2/4/KIRITATI/6/FRET2*2/4/SNI/TRAP#1/3/KAUZ*2/TRAP//KAUZ/5/KACHU | Q2 |
| 129 | SEEDLTPP2720 | 7643073 | CHEN/AE.SQ//2*OPATA/5/SERI.1B//KAUZ/HEVO/3/AMAD*2/4/KIRITATI/6/FRET2*2/4/SNI/TRAP#1/3/KAUZ*2/TRAP//KAUZ/5/KACHU | Q3 |
| 130 | SEEDLTPP2721 | 7643074 | CHEN/AE.SQ//2*OPATA/5/SERI.1B//KAUZ/HEVO/3/AMAD*2/4/KIRITATI/6/FRET2*2/4/SNI/TRAP#1/3/KAUZ*2/TRAP//KAUZ/5/KACHU | Q2 |
| 131 | SEEDLTPP2722 | 7643076 | CHEN/AE.SQ//2*OPATA/5/SERI.1B//KAUZ/HEVO/3/AMAD*2/4/KIRITATI/6/FRET2*2/4/SNI/TRAP#1/3/KAUZ*2/TRAP//KAUZ/5/KACHU | Q2 |
| 132 | SEEDLTPP2724 | 7643080 | CHEN/AE.SQ//2*OPATA/5/SERI.1B//KAUZ/HEVO/3/AMAD*2/4/KIRITATI/6/FRET2*2/4/SNI/TRAP#1/3/KAUZ*2/TRAP//KAUZ/5/KACHU | Q2 |
| 133 | SEEDLTPP2725 | 7643081 | CHEN/AE.SQ//2*OPATA/5/SERI.1B//KAUZ/HEVO/3/AMAD*2/4/KIRITATI/6/FRET2*2/4/SNI/TRAP#1/3/KAUZ*2/TRAP//KAUZ/5/KACHU | Q3 |
| 134 | SEEDLTPP2727 | 7643084 | CHEN/AE.SQ//2*OPATA/5/SERI.1B//KAUZ/HEVO/3/AMAD*2/4/KIRITATI/6/FRET2*2/4/SNI/TRAP#1/3/KAUZ*2/TRAP//KAUZ/5/KACHU | Q3 |
| 135 | SEEDLTPP2730 | 7643090 | CHEN/AE.SQ//2*OPATA/5/SERI.1B//KAUZ/HEVO/3/AMAD*2/4/KIRITATI/6/FRET2*2/4/SNI/TRAP#1/3/KAUZ*2/TRAP//KAUZ/5/KACHU | Q3 |
| 136 | SEEDLTPP2740 | 7643103 | CHEN/AE.SQ//2*OPATA/5/SERI.1B//KAUZ/HEVO/3/AMAD*2/4/KIRITATI/6/FRET2*2/4/SNI/TRAP#1/3/KAUZ*2/TRAP//KAUZ/5/KACHU | Q3 |
| 137 | SEEDLTPP2749 | 7643121 | CHEN/AE.SQ//WEAVER/5/SERI.1B//KAUZ/HEVO/3/AMAD*2/4/KIRITATI/6/FRET2*2/4/SNI/TRAP#1/3/KAUZ*2/TRAP//KAUZ/5/KACHU | Q1 |
| 138 | SEEDLTPP2752 | 7643126 | CHEN/AE.SQ//WEAVER/5/SERI.1B//KAUZ/HEVO/3/AMAD*2/4/KIRITATI/6/FRET2*2/4/SNI/TRAP#1/3/KAUZ*2/TRAP//KAUZ/5/KACHU | Q3 |

All pedigree/crosses detail has already published and available at (<https://seedsofdiscovery.org/wp-content/uploads/sites/52/2017/10/7_MasAgro_Trigo_2.pdf>) ([Wanyera and Owuoche, 2017](#_ENREF_121); [Singh et al., 2018](#_ENREF_114); [Großkinsky et al., 2020](#_ENREF_48))

**Supplementary Table 2.** Range, mean±SD (standard deviation) and ANOVA of traits under control and salinity stress treatments

| **Trait** | **So (Control)** | | **S1 (150mM NaCl)** | | **S2 (250mM NaCl)** | | **ANOVA *(p*-value)** |
| --- | --- | --- | --- | --- | --- | --- | --- |
|  | Range | **Mean±SD** | Range | **Mean±SD** | Range | **Mean±SD** |  |
| TG | 60-100 | 91.10±9.63 | 32-99 | 68.84±20.19 | 18-91 | 53.22±20.95 | P_G_ = ***, P_T_ = ***, P_G*T_ = ** |
| RN | 3-6 | 4.52±0.44 | 4-6 | 5.13±0.31 | 4-8 | 5.12±0.45 | P_G_ = ***, P = ***, P_G*T_ = *** |
| CL | 2-3.8 | 2.76±0.27 | 2-4.3 | 2.94±0.48 | 1.2-3.5 | 2.34±0.45 | P_G_ = ***, P_T_ = ***, P_G*T_ = *** |
| SL | 6.2-17 | 9.38±1.28 | 4.1-8.8 | 6.24±0.95 | 1.4-6 | 3.04±0.79 | P_G_ = *, P_T_= *** |
| RL | 4.2-13.9 | 9.35±1.81 | 2.8-10 | 6.08±1.79 | 1-6.1 | 3.25±1.14 | P_G_ = ***, P_T_ = ***, P_G*T_ = *** |
| R/S | 0.8-1.3 | 1.14±0.04 | 0.5-1.6 | 0.98±0.23 | 0.4-2.5 | 1.11±0.38 | P_G_ = ***, P _T_= ***, P_G*T_ = *** |
| SVI | 1000-2327 | 1708±300 | 310-1820 | 877±380 | 64-813 | 153±200.76 | P_G_ = ***, P_T_ = ***, P_G*T_ = *** |

TG = Total germination percentage, RN =Number of roots, CL = Coleoptile length (cm), SL = Shoot length(cm), RL = Root length (cm),R/S = Root to shoot ratio and SVI = Seedling vigor index, P_G_ = *p* value of genotypes effect, P_T_ = *p* value of treatments effect, P_G*T_ = *p* value of genotypes and treatments interaction. *, ** and *** indicate significant differences at *p*-values of 0.05, 0.01 and 0.001, respectively.

**Supplementary Table 3.** Range, mean±SD (standard deviation) and ANOVA of relative traits under salinity stress treatments

|  | **Relative at (150Mm) NaCl** | | **Relative at (250Mm) NaCl** | | **ANOVA *(p*-value)** |
| --- | --- | --- | --- | --- | --- |
| **Trait** | Range | **Mean±SD** | Range | **Mean±SD** |  |
| RTG | 35-99 | 75.06±18.59 | 23-91 | 57.71±20.04 | P_G_ = ***, P_T_ = ***, P_G*T_ = *** |
| RRN | 80-151 | 114±12.69 | 90-169 | 114.27±12.92 | P_G_ = ***, P_T_ = ***, P_G*T_ = *** |
| RCL | 63-154 | 106.78±16.77 | 37-138 | 85.62±18.84 | P_G_ = ***, P_T_ = ***, P_G*T_ = *** |
| RSL | 31-96 | 67.30±11.28 | 13-64 | 33.02±9.38 | P_G_ = ***, P_T_ = ***, P_G*T_ = *** |
| RRL | 33-111 | 65.67±17.17 | 12-78 | 35.36±12.11 | P_G_ = ***, P_T_ = ***, P_G*T_ = *** |
| RR/S | 53-236 | 99.50±28.50 | 42-293 | 112.64±41.00 | P_G_ = ***, P_T_ = ***, P_G*T_ = *** |
| RSVI | 19-81 | 50.77±18.27 | 4-47 | 20.38±10.54 | P_G_ = ***, P_T_ = ***, P_G*T_ = *** |

RTG = Relative total germination percentage, RRN =Relative number of roots, RCL = Relative coleoptile length (cm), RSL = Relative shoot length (cm), RRL = Relative root length (cm),RR/S = Relative root to shoot ratio and SRVI = Relative seedling vigor index, P_G_ = *p* value of genotypes effect, P_T_ = *p* value of treatments effect, P_G*T_ = *p* value of genotypes and treatments interaction. *, ** and *** indicate significant differences at *p*-values of 0.05, 0.01 and 0.001, respectively.

Supplementary Table 4. Correlations among the traits measured in So, S1 and S2. Only significant correlations at *p-*value 0.05 (*) and 0.001 (**) are shown

|  | TG_So | RN_So | CL_So | SL_So | RL_So | R/S_So | SVI_So | TG_S1 | RN_S1 | CL_S1 | SL_S1 | RL_S1 | R/S_S1 | SVI_S1 |
| --- | --- | --- | --- | --- | --- | --- | --- | --- | --- | --- | --- | --- | --- | --- |
| TG_So | 1 |  |  |  |  |  |  |  |  |  |  |  |  |  |
| RN_So |  | 1 |  |  |  |  |  |  |  |  |  |  |  |  |
| CL_So |  | 0.20^*^ | 1 |  |  |  |  |  |  |  |  |  |  |  |
| SL_So | -0.22^**^ | 0.26^**^ | 0.27^**^ | 1 |  |  |  |  |  |  |  |  |  |  |
| RL_So | 0.28^**^ |  | 0.22^**^ | 0.21^*^ | 1 |  |  |  |  |  |  |  |  |  |
| R/S_So |  |  |  | -0.19^*^ |  | 1 |  |  |  |  |  |  |  |  |
| SVI_So | 0.67^**^ |  | 0.27^**^ | 0.36^**^ | 0.82^**^ | -0.17^*^ | 1 |  |  |  |  |  |  |  |
| TG_S1 | 0.56^**^ |  |  |  | 0.21^*^ |  | 0.42^**^ | 1 |  |  |  |  |  |  |
| RN_S1 |  | 0.20^*^ |  |  |  |  |  |  | 1 |  |  |  |  |  |
| CL_S1 | 0.18^*^ | 0.28^**^ | 0.34^**^ |  | 0.19^*^ | 0.18^*^ | 0.28^**^ | 0.38^**^ |  | 1 |  |  |  |  |
| SL_S1 | 0.27^**^ | 0.24^**^ | 0.18^*^ | 0.18^*^ | 0.29^**^ |  | 0.40^**^ | 0.46^**^ |  | 0.58^**^ | 1 |  |  |  |
| RL_S1 | 0.40^**^ | 0.18^*^ |  | 0.18^*^ | 0.50^**^ |  | 0.59^**^ | 0.58^**^ |  | 0.54^**^ | 0.63^**^ | 1 |  |  |
| R/S_S1 | 0.32^**^ |  |  |  | 0.41^**^ |  | 0.46^**^ | 0.41^**^ |  | 0.30^**^ |  | 0.83^**^ | 1 |  |
| SVI_S1 | 0.54^**^ |  |  |  | 0.38^**^ |  | 0.56^**^ | 0.91^**^ |  | 0.52^**^ | 0.68^**^ | 0.83^**^ | 0.57^**^ | 1 |

**Supplementary Table 4** Conti…

|  | TG_So | RN_So | CL_So | SL_So | RL_So | R/S_So | SVI_So | TG_S2 | RN_S2 | CL_S2 | SL_S2 | RL_S2 | R/S_S2 | SVI_S2 |
| --- | --- | --- | --- | --- | --- | --- | --- | --- | --- | --- | --- | --- | --- | --- |
| TG_So | 1 |  |  |  |  |  |  |  |  |  |  |  |  |  |
| RN_So |  | 1 |  |  |  |  |  |  |  |  |  |  |  |  |
| CL_So |  | 0.20^*^ | 1 |  |  |  |  |  |  |  |  |  |  |  |
| SL_So | -0.22^**^ | 0.26^**^ | 0.27^**^ | 1 |  |  |  |  |  |  |  |  |  |  |
| RL_So | 0.28^**^ |  | 0.22^**^ | 0.21^*^ | 1 |  |  |  |  |  |  |  |  |  |
| R/S_So |  |  |  | -0.19^*^ |  | 1 |  |  |  |  |  |  |  |  |
| SVI_So | 0.67^**^ |  | 0.27^**^ | 0.36^**^ | 0.82^**^ | -0.17^*^ | 1 |  |  |  |  |  |  |  |
| TG_S2 | 0.55^**^ |  |  |  | 0.25^**^ |  | 0.44^**^ | 1 |  |  |  |  |  |  |
| RN_S2 |  | 0.30^**^ |  |  |  |  |  |  | 1 |  |  |  |  |  |
| CL_S2 |  |  |  | -0.20^**^ |  |  |  | 0.28^**^ |  | 1 |  |  |  |  |
| SL_S2 |  |  |  |  |  |  |  |  |  | 0.65^**^ | 1 |  |  |  |
| RL_S2 | 0.38^**^ |  |  |  | 0.38^**^ |  | 0.39^**^ | 0.58^**^ |  | 0.60^**^ | 0.47^**^ | 1 |  |  |
| R/S_S2 | 0.34^**^ |  |  |  | 0.31^**^ |  | 0.37^**^ | 0.49^**^ |  |  | -0.36^**^ | 0.60^**^ | 1 |  |
| SVI_S2 | 0.49^**^ |  |  |  | 0.33^**^ |  | 0.43^**^ | 0.88^**^ |  | 0.57^**^ | 0.50^**^ | 0.84^**^ | 0.43^**^ | 1 |

**Supplementary Table 4** Conti…

|  | TG_S1 | RN_S1 | CL_S1 | SL_S1 | RL_S1 | R/S_S1 | SVI_S1 | TG_S2 | RN_S2 | CL_S2 | SL_S2 | RL_S2 | R/S_S2 | SVI_S2 |
| --- | --- | --- | --- | --- | --- | --- | --- | --- | --- | --- | --- | --- | --- | --- |
| TG_S1 | 1 |  |  |  |  |  |  |  |  |  |  |  |  |  |
| RN_S1 |  | 1 |  |  |  |  |  |  |  |  |  |  |  |  |
| CL_S1 | 0.38^**^ |  | 1 |  |  |  |  |  |  |  |  |  |  |  |
| SL_S1 | 0.46^**^ |  | 0.58^**^ | 1 |  |  |  |  |  |  |  |  |  |  |
| RL_S1 | 0.58^**^ |  | 0.54^**^ | 0.63^**^ | 1 |  |  |  |  |  |  |  |  |  |
| R/S_S1 | 0.41^**^ |  | 0.30^**^ |  | 0.83^**^ | 1 |  |  |  |  |  |  |  |  |
| SVI_S1 | 0.91^**^ |  | 0.52^**^ | 0.68^**^ | 0.83^**^ | 0.57^**^ | 1 |  |  |  |  |  |  |  |
| TG_S2 | 0.89^**^ |  | 0.34^**^ | 0.43^**^ | 0.62^**^ | 0.48*^*^ | 0.86^**^ | 1 |  |  |  |  |  |  |
| RN_S2 |  | 0.24^**^ |  | 0.21^*^ |  |  |  |  | 1 |  |  |  |  |  |
| CL_S2 | 0.22^**^ |  |  |  |  |  |  | 0.28^**^ |  | 1 |  |  |  |  |
| SL_S2 |  |  | -0.26^**^ | -0.21^**^ | -0.20^**^ |  |  |  |  | 0.65^**^ | 1 |  |  |  |
| RL_S2 | 0.49^**^ |  | 0.19^**^ |  | 0.48^**^ | 0.56^**^ | 0.51^**^ | 0.58^**^ |  | 0.60^**^ | 0.47^**^ | 1 |  |  |
| R/S_S2 | 0.49^**^ |  | 0.43^**^ | 0.27^**^ | 0.68^**^ | 0.65^**^ | 0.60^**^ | 0.49^**^ |  |  | -0.36^**^ | 0.60^**^ | 1 |  |
| SVI_S2 | 0.76^**^ |  | 0.25^**^ | 0.28^**^ | 0.55^**^ | 0.49^**^ | 0.73^**^ | 0.88^**^ |  | 0.57^**^ | 0.50^**^ | 0.84^**^ | 0.43^**^ | 1 |

**Supplementary Table 4** Conti…

|  | RTG_S1 | RRN_S1 | RCL_S1 | RSL_S1 | RRL_S1 | RR/S_S1 | RSVI_S1 | RTG_S2 | RRN_S2 | RCL_S2 | RSL_S2 | RRL_S2 | RR/S_S2 | RSVI_S2 |
| --- | --- | --- | --- | --- | --- | --- | --- | --- | --- | --- | --- | --- | --- | --- |
| RTG_  S1 | 1 |  |  |  |  |  |  |  |  |  |  |  |  |  |
| RRN_  S1 |  | 1 |  |  |  |  |  |  |  |  |  |  |  |  |
| RCL_  S1 | 0.39^**^ | -0.20^*^ | 1 |  |  |  |  |  |  |  |  |  |  |  |
| RSL_  S1 | 0.38^**^ |  | 0.44^**^ | 1 |  |  |  |  |  |  |  |  |  |  |
| RRL_  S1 | 0.47^**^ |  | 0.52^**^ |  | 1 |  |  |  |  |  |  |  |  |  |
| RR/S_S1 | 0.17^*^ | -0.19^*^ | -0.22^*^ | -0.31^**^ | 0.70^**^ | 1 |  |  |  |  |  |  |  |  |
| RSVI_S1 | 0.90^**^ |  | 0.54^**^ | 0.62^**^ | 0.75^**^ | 0.27^**^ | 1 |  |  |  |  |  |  |  |
| RTG_  S2 | 0.85^**^ |  | 0.34^**^ | 0.34^**^ | 0.51^**^ | 0.24^**^ | 0.82^**^ | 1 |  |  |  |  |  |  |
| RRN_  S2 |  | 0.67^**^ |  |  |  | -0.20^*^ |  | 0 | 1 |  |  |  |  |  |
| RCL_  S2 | 0.21^*^ |  | 0.29^**^ |  |  |  | 0.20^*^ | 0.27^**^ | 0 | 1 |  |  |  |  |
| RSL_  S2 |  | 0.24^**^ |  |  | -0.23^**^ | -0.33^**^ |  |  | 0.23^**^ | 0.66^**^ | 1 |  |  |  |
| RRL_  S2 | 0.34^*^ |  | 0.22^**^ |  | 0.42^**^ | 0.29^**^ | 0.41^**^ | 0.43^**^ |  | 0.57^**^ | 0.53^**^ | 1 |  |  |
| RR/S_S2 | 0.32^**^ |  | 0.35^**^ |  | 0.64^**^ | 0.66^**^ | 0.42^**^ | 0.33^**^ | -0.17^*^ |  | 0.44^**^ | 0.46^**^ | 1 |  |
| RSVI_S2 | 0.67^**^ |  | 0.29^**^ | 0.33^**^ | 0.43^**^ |  | 0.68^**^ | 0.83^**^ |  | 0.60^**^ | 0.55^**^ | 0.78*^*^ | 0.22^**^ | 1 |

**Supplementary Table 5** Factors linked to correlated traits, selection differential, heritability, selection gains and objectives.

| **Variables** | **Factor** | **SD** | **SD%** | **h^2^** | **SG** | **SG%** | **objective** |
| --- | --- | --- | --- | --- | --- | --- | --- |
| **Control-S0** | | | | | | | |
| TG | FA1 | 2.20 | 2.42 | 0.98 | 2.17 | 2.38 | increase |
| RL | FA1 | 1.81 | 19.38 | 1.00 | 1.81 | 19.37 | increase |
| R_S | FA1 | 0.03 | 2.83 | 1.00 | 0.03 | 2.83 | increase |
| SVI | FA1 | 357.22 | 20.87 | 0.99 | 354.98 | 20.74 | increase |
| RN | FA2 | 0.17 | 3.63 | 0.73 | 0.12 | 2.66 | increase |
| CL | FA2 | 0.27 | 9.77 | 0.98 | 0.26 | 9.56 | increase |
| SL | FA2 | 1.62 | 17.24 | 1.00 | 1.62 | 17.22 | increase |
| **150 mM-S1** | | | | | | | |
| TG | FA1 | 14.65 | 21.28 | 0.97 | 14.27 | 20.73 | increase |
| CL | FA1 | 0.35 | 11.94 | 0.79 | 0.28 | 9.42 | increase |
| SL | FA1 | 0.66 | 10.58 | 0.74 | 0.49 | 7.81 | increase |
| SVI | FA1 | 404.42 | 46.11 | 0.96 | 387.95 | 44.23 | increase |
| RN | FA2 | 0.18 | 3.49 | 0.41 | 0.07 | 1.42 | increase |
| RL | FA3 | 1.86 | 30.63 | 0.91 | 1.70 | 27.91 | increase |
| R_S | FA3 | 0.15 | 15.84 | 0.88 | 0.14 | 13.89 | increase |
| **250 mM-S2** | | | | | | | |
| TG | FA1 | 21.70 | 40.77 | 0.95 | 20.59 | 38.69 | increase |
| RL | FA1 | 0.98 | 30.01 | 0.82 | 0.80 | 24.67 | increase |
| R_S | FA1 | 0.07 | 6.30 | 0.81 | 0.06 | 5.10 | increase |
| SVI | FA1 | 262.15 | 74.32 | 0.94 | 245.55 | 69.61 | increase |
| CL | FA2 | 0.25 | 10.52 | 0.76 | 0.19 | 7.99 | increase |
| SL | FA2 | 0.64 | 21.06 | 0.82 | 0.52 | 17.21 | increase |
| RN | FA3 | 0.23 | 4.48 | 0.51 | 0.12 | 2.28 | increase |

SD, selection differential; SG, selection gains; h^2^, heritability.

TG, Total germination percentage; RN, Numbers of roots; CL, Coleoptile length; SL, Shoot length; RL, Roots length; R/S, Root to shoot length ratio; SVI, Seedling vigor index.

**Supplementary Table 6** Allelic and phenotypic profiles of the eleven best lines performed under salt stress. Underlined and bold A, G, C and T indicate positive alleles.

| **rs#** | **alleles** | **Chr.** | **Position (Cm)** | **Trait** | **G_35** | **G_58** | **G_85** | **G_86** | **G_108** | **G_109** | **G_113** | **G_114** | **G_115** | **G_118** |
| --- | --- | --- | --- | --- | --- | --- | --- | --- | --- | --- | --- | --- | --- | --- |
| M406 | C/A | 1A | **155.92** | **RL_S2** | N | **C** | **C** | **C** | **C** | A | M | A | **C** | N |
| M1426 | T/C | 1B | **37.65** | **RR/S_S1** | **T** | **T** | **T** | **T** | **T** | **T** | **T** | **T** | **T** | **T** |
| M11993 | G/A | 1B | **61.58** | **SL_S0** | **G** | **G** | A | **G** | **G** | **G** | **G** | **G** | **G** | **G** |
| M11428 | A/T | 1B | **384.13** | **SL_S0** | **A** | **A** | **A** | **A** | **A** | **A** | **A** | **A** | **A** | **A** |
| M9978 | G/T | 1B | **492.15** | **RN_S1** | **G** | **G** | **G** | **G** | T | **G** | **G** | **G** | **G** | **G** |
| M10810 | T/C | 1D | **22.56** | **RRN_S2** | **T** | N | **T** | **T** | **T** | **T** | **T** | **T** | **T** | **T** |
| M10295 | G/A | 1D | **90.1** | **SL_S0** | **G** | **G** | **G** | **G** | **G** | **G** | **G** | **G** | A | **G** |
| M10566 | T/C | 1D | **93.71** | **RR/S_S1** | **T** | **T** | **T** | **T** | **T** | **T** | **T** | **T** | **T** | **T** |
| M8113 | T/C | 1D | **167.7** | **SL_S0** | **T** | T | N | N | T | T | T | T | N | **T** |
| M4431 | A/G | 2A | **125.28** | **SL_S0, RR/S_S1** | **A** | N | **A** | N | **A** | **A** | N | N | **A** | **A** |
| M10796 | G/C | 2A | **159.56** | **RRN_S2** | **G** | **G** | **G** | **G** | **G** | **G** | **G** | **G** | **G** | **G** |
| M765 | C/A | 2A | **221.1** | **R/S_S2** | **C** | A | **C** | N | **C** | **C** | **C** | **C** | A | **C** |
| M9176 | G/A | 2A | **231.79** | **RN_S2** | **G** | **G** | **G** | **G** | **G** | **G** | **G** | **G** | **G** | N |
| M2717 | T/C | 2B | **147.53** | **RR/S_S1** | **T** | **T** | **T** | **T** | **T** | **T** | **T** | **T** | **T** | **T** |
| M627 | G/A | 2D | **289.68** | **RRN_S2** | **G** | **G** | **G** | R | **G** | **G** | **G** | **G** | **G** | R |
| M1930 | A/G | 3A | **75.97** | **RN_S2** | **A** | G | **A** | **A** | **A** | **A** | G | G | **A** | **A** |
| M5307 | G/A | 3B | **68.2** | **RRN_S2** | **G** | **G** | **G** | **G** | **G** | **G** | **G** | R | **G** | **G** |
| M7873 | T/C | 3B | **103.59** | **RR/S_S1, SL_S0** | N | **T** | **T** | **T** | C | C | C | C | **T** | **T** |
| M8515 | G/A | 3B | **162.58** | **SL_S0,RSL_S1, RR/S_S1** | **G** | **G** | **G** | **G** | **G** | **G** | **G** | **G** | **G** | **G** |
| M9138 | G/T | 3B | **222.96** | **RR/S_S1** | **G** | **G** | T | N | **G** | **G** | **G** | N | **G** | **G** |
| M11925 | T/C | 3B | **227.41** | **RN_S2** | **T** | **T** | **T** | **T** | **T** | **T** | **T** | **T** | **T** | **T** |
| M2025 | A/C | 3B | **297.56** | **RN_S2** | **A** | **A** | **A** | C | **A** | **A** | **A** | **A** | **A** | **A** |
| M1987 | G/A | 3D | **107.75** | **RR/S_S1** | **G** | **G** | **G** | **G** | **G** | **G** | **G** | **G** | **G** | **G** |
| M1398 | G/A | 4A | **49.56** | **SL_S0** | **G** | **G** | **G** | **G** | **G** | **G** | **G** | **G** | **G** | **G** |
| M11711 | A/G | 4A | **180.05** | **SL_S0, RL_S1, RR/S_S1** | G | **A** | **A** | **A** | **A** | N | **A** | **A** | **A** | **A** |
| M5589 | G/A | 4A | **215.47** | **RN_S1** | R | **G** | **G** | A | **G** | **G** | **G** | **G** | **G** | **G** |
| M4103 | T/C | 4D | **34.78** | **RRN_S2** | **T** | **T** | **T** | **T** | **T** | **T** | **T** | **T** | **T** | **T** |
| M3343 | T/C | 4D | **50.31** | **RRN_S2** | **T** | **T** | **T** | **T** | **T** | **T** | **T** | **T** | **T** | **T** |
| M1974 | C/T | 4D | **157.4** | **RR/S_S1** | **C** | **C** | **C** | **C** | **C** | **C** | N | N | **C** | **C** |
| M8885 | G/T | 5A | **113.15** | **RR/S_S1, SL_S0** | **G** | T | T | **G** | **G** | **G** | **G** | **G** | **G** | N |
| M11486 | G/A | 5A | **161.09** | **RRN_S1** | A | A | **G** | **G** | **G** | **G** | **G** | **G** | **G** | **G** |
| M4314 | A/G | 5A | **209.83** | **RN_S2** | **A** | **A** | **A** | **A** | **A** | **A** | **A** | **A** | **A** | **A** |
| M3034 | A/G | 5A | **229.39** | **RN_S2** | N | **A** | **A** | **A** | **A** | **A** | **A** | **A** | **A** | **A** |
| M2139 | A/G | 5B | **56.97** | **RN_S2** | **A** | **A** | **A** | N | **A** | **A** | **A** | **A** | **A** | **A** |
| M4710 | T/C | 5B | **56.97** | **RN_S2** | **T** | **T** | **T** | **T** | **T** | **T** | **T** | **T** | **T** | **T** |
| M1450 | T/C | 5B | **125.96** | **RN_S1** | **T** | **T** | **T** | **T** | C | **T** | **T** | **T** | **T** | **T** |
| M11102 | T/A | 5D | **195.73** | **RN_S2** | **T** | **T** | **T** | N | **T** | **T** | **T** | **T** | **T** | **T** |
| M7775 | C/G | 5D | **210.31** | **RN_S2** | **C** | G | **C** | N | **C** | **C** | **C** | **C** | **C** | **C** |
| M337 | T/C | 5D | **232.48** | **RRN_S2** | **T** | **T** | **T** | **T** | **T** | **T** | **T** | N | **T** | **T** |
| M5347 | C/G | 6A | **65.28** | **RN_S2** | **C** | **C** | **C** | **C** | **C** | **C** | **C** | **C** | **C** | **C** |
| M530 | C/T | 6A | **73.17** | **RN_S2** | **C** | **C** | **C** | **C** | **C** | **C** | **C** | **C** | **C** | **C** |
| M4362 | A/T | 6B | **69.05** | **RR/S_S1** | **A** | **A** | **A** | **A** | **A** | **A** | **A** | **A** | **A** | **A** |
| M11763 | T/C | 6D | **90.8** | **RR/S_S1** | **T** | **T** | **T** | **T** | **T** | **T** | C | **T** | **T** | N |
| M1188 | G/C | 6D | **121.22** | **SL_S0** | **G** | **G** | S | **G** | **G** | **G** | S | **G** | S | **G** |
| M10047 | T/C | 7A | **45.99** | **RR/S_S1** | **T** | **T** | **T** | **T** | **T** | **T** | **T** | **T** | **T** | **T** |
| M9550 | G/T | 7A | **170.91** | **RRN_S2** | **G** | K | **G** | **G** | **G** | **G** | **G** | **G** | **G** | **G** |
| M38 | G/A | 7A | **214.7** | **RL_S0** | **G** | **G** | N | **G** | N | **G** | N | **G** | **G** | R |
| M9660 | T/C | 7A | **276.04** | **RN_S1** | Y | **T** | **T** | Y | C | Y | **T** | **T** | **T** | **T** |
|  |  | Numbers of Positive alleles | | | **41** | **40** | **42** | **37** | **43** | **44** | **38** | **38** | **44** | **42** |

**Supplementary Table 6** Conti…..

| **Trait** | G_35 | G_58 | G_85 | G_86 | G_108 | G_109 | G_113 | G_114 | G_115 | G_118 | sample Mean | Population mean | % increased |
| --- | --- | --- | --- | --- | --- | --- | --- | --- | --- | --- | --- | --- | --- |
| TG_S0 | 95 | 80 | 88 | 92 | 92 | 100 | 100 | 88 | 96 | 96 | 92.7±5.83 | 91.1±9.63 | 1.723115 |
| RN_S0 | 4 | 4.5 | 5 | 4 | 5 | 4 | 4 | 4 | 4 | 4 | 4.25±0.40 | 4.52±0.44 | -6.29339 |
| CL_S0 | 2.5 | 2.9 | 3.1 | 2.9 | 3 | 2 | 3 | 2.6 | 2.7 | 2.9 | 2.76±0.31 | 2.76±0.27 | -0.02951 |
| SL_S0 | 7.6 | 8.8 | 10.4 | 9 | 9.2 | 6.9 | 9.1 | 8.9 | 8.3 | 7.6 | 8.58±0.96 | 9.38±1.28 | -9.3339 |
| RL_S0 | 4.2 | 7.5 | 8.9 | 8.2 | 9.5 | 8.1 | 11.9 | 9.1 | 9.6 | 10.3 | 8.73±1.92 | 9.35±1.81 | -7.09891 |
| R_S_S0 | 0.6 | 0.9 | 0.9 | 0.9 | 1 | 1.2 | 1.3 | 1 | 1.2 | 1.4 | 1.04±0.22 | 1.14±0.04 | -9.75573 |
| SVI_S0 | 1127 | 1305 | 1701 | 1586 | 1719 | 1503 | 2103 | 1586 | 1724 | 1718 | 1607.2±250.34 | 1708±300 | -6.30285 |
| TG_S1 | 46 | 63 | 68 | 85 | 86 | 87 | 97 | 84 | 48 | 85 | 74.9±16.71 | 68.84±20.19 | 8.089149 |
| RN_S1 | 6 | 5 | 5 | 5 | 6 | 5 | 5 | 5 | 5 | 5 | 5.2±0.4 | 5.13±0.31 | 1.389903 |
| CL_S1 | 2.5 | 3 | 3.9 | 3.3 | 3.1 | 2.9 | 3.6 | 3.3 | 3.3 | 3.1 | 3.2±0.36 | 2.94±0.48 | 8.25457 |
| SL_S1 | 6.4 | 6.6 | 8.6 | 7.1 | 5.9 | 6.6 | 6.8 | 7.4 | 6.8 | 5.7 | 6.79±0.77 | 6.24±0.95 | 8.137132 |
| RL_S1 | 3.9 | 4.7 | 8 | 7.7 | 8.7 | 6.8 | 7.6 | 7.7 | 5.8 | 5.8 | 6.67±1.48 | 6.08±1.79 | 8.878241 |
| R_S_S1 | 0.6 | 0.7 | 0.9 | 1.1 | 1.5 | 1 | 1.1 | 1 | 0.9 | 1 | 0.98±0.23 | 0.98±0.23 | 0.520459 |
| SVI_S1 | 478 | 711 | 1121 | 1248 | 1246 | 1167 | 1397 | 1275 | 608 | 978 | 1022.9±300.45 | 877±380 | 14.24965 |
| RTG_S1 | 48 | 78 | 77 | 92 | 93 | 87 | 97 | 95 | 50 | 89 | 80.6±17.00 | 75.06±18.59 | 6.870025 |
| RRN_S1 | 151 | 118 | 100 | 114 | 109 | 129 | 132 | 117 | 108 | 111 | 118.9±14.031749712705 | 114.2±12.69 | 3.951872 |
| RCL_S1 | 99 | 102 | 125 | 115 | 104 | 146 | 120 | 127 | 121 | 106 | 116.5±13.68 | 106.78±16.77 | 8.342755 |
| RSL_S1 | 85 | 76 | 83 | 78 | 64 | 96 | 74 | 84 | 82 | 76 | 79.8±7.98 | 67.3±11.28 | 15.658 |
| RRL_S1 | 93 | 63 | 89 | 94 | 92 | 84 | 64 | 85 | 61 | 56 | 78.1±14.41 | 65.67±17.17 | 15.92159 |
| RR_S_S1 | 111 | 84 | 108 | 124 | 146 | 87 | 86 | 101 | 74 | 73 | 99.4±22.14 | 99.5±28.5 | -0.10097 |
| RSVI_S1 | 42 | 54 | 66 | 79 | 72 | 78 | 66 | 80 | 35 | 57 | 62.9±14.87 | 50.77±18.27 | 19.28686 |
| TG_S2 | 31 | 44 | 44 | 77 | 72 | 80 | 79 | 63 | 29 | 61 | 58±18.65 | 53.22±20.95 | 8.235596 |
| RN_S2 | 5 | 5 | 5 | 7 | 6 | 5 | 5 | 5 | 5 | 5 | 5.3±0.64 | 5.12±0.45 | 3.31989 |
| CL_S2 | 2.1 | 2.7 | 1.4 | 2.4 | 2.6 | 2.8 | 3.5 | 3.4 | 2 | 2.5 | 2.54±0.60 | 2.34±0.45 | 7.914589 |
| SL_S2 | 3.3 | 3.2 | 1.5 | 2.6 | 3.1 | 3.7 | 3.6 | 4.7 | 2 | 2.6 | 3.03±0.8 | 3.04±0.79 | -0.40069 |
| RL_S2 | 2.6 | 3.2 | 2.5 | 3.3 | 4.8 | 5.1 | 5.4 | 5.6 | 2.3 | 3.3 | 3.81±1.21 | 3.25±1.14 | 14.62152 |
| R_S_S2 | 0.8 | 1 | 1.7 | 1.3 | 1.5 | 1.4 | 1.5 | 1.2 | 1.1 | 1.3 | 1.28±0.25 | 1.10±0.38 | 13.22579 |
| SVI_S2 | 192 | 271 | 175 | 455 | 569 | 704 | 709 | 645 | 125 | 362 | 420.7±215.13 | 353±200.95 | 16.15238 |
| RTG_S2 | 33 | 55 | 50 | 84 | 78 | 80 | 79 | 71 | 30 | 64 | 62.4±18.69 | 57.71±20.04 | 7.516492 |
| RRN_S2 | 121 | 106 | 106 | 151 | 107 | 130 | 118 | 113 | 108 | 122 | 118.2±13.39 | 114.27±12.92 | 3.325642 |
| RCL_S2 | 85 | 93 | 46 | 83 | 88 | 138 | 116 | 129 | 74 | 87 | 93.9±25.76 | 85.62±18.84 | 8.817932 |
| RSL_S2 | 43 | 36 | 14 | 29 | 34 | 53 | 39 | 52 | 24 | 34 | 35.8±11.34 | 33.01±9.38 | 7.761206 |
| RRL_S2 | 62 | 42 | 28 | 40 | 50 | 63 | 46 | 61 | 24 | 32 | 44.8±13.51 | 35.36±12.11 | 21.06223 |
| RR_S_S2 | 136 | 113 | 203 | 138 | 149 | 119 | 116 | 117 | 98 | 94 | 128.3±29.76 | 112.64±41 | 12.20372 |
| RSVI_S2 | 17 | 21 | 10 | 29 | 33 | 47 | 34 | 41 | 7 | 21 | 26±12.39 | 20.38±10.55 | 21.60841 |
