# Supplementary material for: A GBS-based genome-wide association study reveals the genetic basis of salinity tolerance at the seedling stage in bread wheat (Triticum aestivum L.)
Source: Front Genet. 2022 Sep 27;13:997901. doi: 10.3389/fgene.2022.997901 (PMC9551609; doi:10.3389/fgene.2022.997901)
Supplement: Supplementary file 9 [file Table3.pdf]

Supplementary Table S3. Range, mean±SD (standard deviation) and ANOVA of relative traits under salinity stress treatments

	**Relative at (150Mm) NaCl**		**Relative at (250Mm) NaCl**		**ANOVA *(p*-value)**
**Trait**	Range	**Mean±SD**	Range	**Mean±SD**	
RTG	35-99	75.06±18.59	23-91	57.71±20.04	P_G = ***, P_T = ***, P_{G*T} = ***
RRN	80-151	114±12.69	90-169	114.27±12.92	P_G = ***, P_T = ***, P_{G*T} = ***
RCL	63-154	106.78±16.77	37-138	85.62±18.84	P_G = ***, P_T = ***, P_{G*T} = ***
RSL	31-96	67.30±11.28	13-64	33.02±9.38	P_G = ***, P_T = ***, P_{G*T} = ***
RRL	33-111	65.67±17.17	12-78	35.36±12.11	P_G = ***, P_T = ***, P_{G*T} = ***
RR/S	53-236	99.50±28.50	42-293	112.64±41.00	P_G = ***, P_T = ***, P_{G*T} = ***
RSVI	19-81	50.77±18.27	4-47	20.38±10.54	P_G = ***, P_T = ***, P_{G*T} = ***

RTG = Relative total germination percentage, RRN =Relative number of roots, RCL = Relative coleoptile length (cm), RSL = Relative shoot length (cm), RRL = Relative root length (cm),RR/S = Relative root to shoot ratio and SRVI = Relative seedling vigor index, P_G = *p* value of genotypes effect, P_T = *p* value of treatments effect, P_{G*T} = *p* value of genotypes and treatments interaction. *, ** and *** indicate significant differences at *p*-values of 0.05, 0.01 and 0.001, respectively.
