# Supplementary material for: A GBS-based genome-wide association study reveals the genetic basis of salinity tolerance at the seedling stage in bread wheat (Triticum aestivum L.)
Source: Front Genet. 2022 Sep 27;13:997901. doi: 10.3389/fgene.2022.997901 (PMC9551609; doi:10.3389/fgene.2022.997901)
Supplement: Supplementary file 10 [file Image1.pdf]

Supplementary Material

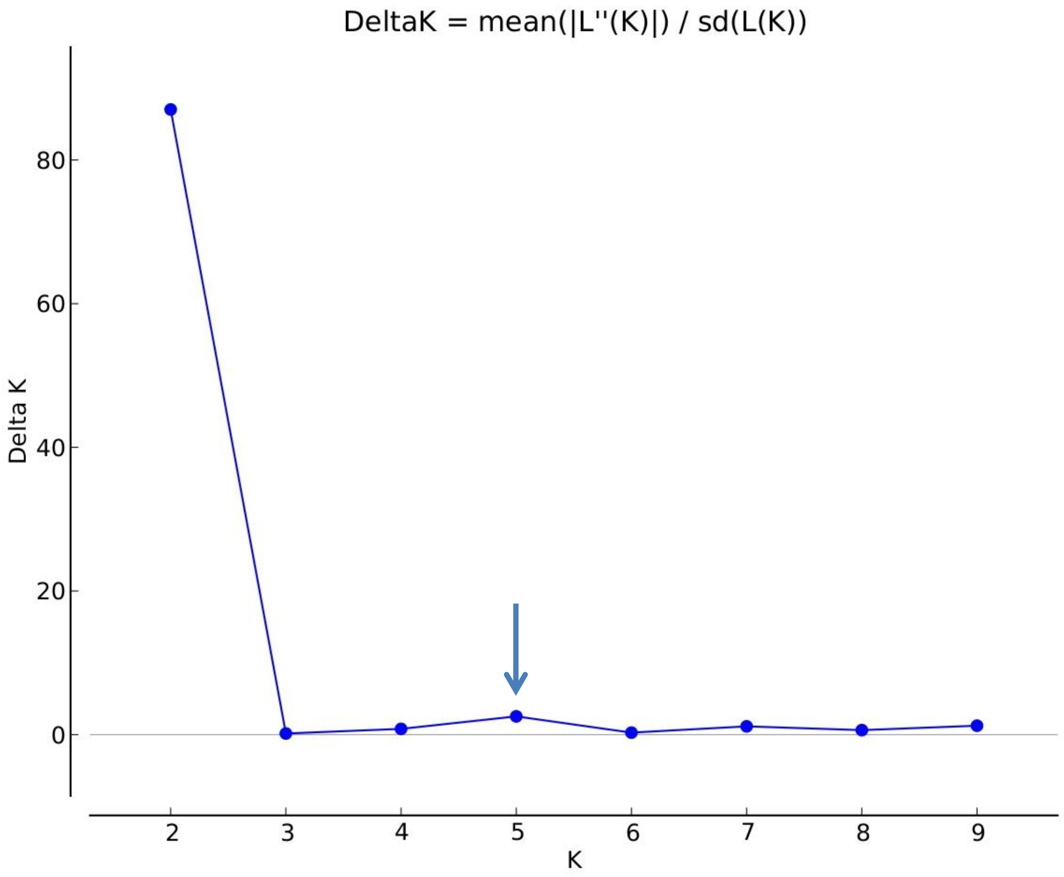

Supplementary Figure S1 ΔK versus sub-groups K plot with arrow pointing to the number of sub-populations in the germplasm
